# Evaluation of the Early Adolescent Skills for Emotions (EASE) intervention in Lebanon: A randomized controlled trial

**DOI:** 10.1016/j.comppsych.2023.152424

**Published:** 2023-09-16

**Authors:** Mark J.D. Jordans, Felicity L. Brown, Jeremy Kane, Karine Taha, Frederik Steen, Rayane Ali, Joseph Elias, Bassel Meksassi, May Aoun, Claire M. Greene, Aiysha Malik, Aemal Akhtar, Mark van Ommeren, Marit Sijbrandij, Richard Bryant

**Affiliations:** a War Child, Research and Development Department, Amsterdam, the Netherlands; b Amsterdam Institute of Social Science Research, University of Amsterdam, Amsterdam, the Netherlands; c Department of Epidemiology, Columbia University, New York, USA; d War Child, Lebanon Country Office, Beirut, Lebanon; e Program on Forced Migration and Health, Heilbrunn Department of Population and Family Health, Columbia University Mailman School of Public Health, USA; f World Health Organization, Geneva, Switzerland; g Department of Clinical Neuroscience, Division of Insurance Medicine, Karolinska Institutet, Sweden; h School of Psychology, University of New South Wales, Australia; i VU University, Department of Clinical, Neuro- and Developmental Psychology, WHO Collaborating Center for Research and Dissemination of Psychological Interventions, Amsterdam, the Netherlands

**Keywords:** Randomized controlled trial, Psychological intervention, Refugee mental health, Adolescents, Lebanon

## Abstract

**Background:**

There is a need for scalable evidence-based psychological interventions for young adolescents experiencing high levels of psychological distress in humanitarian settings and low- and middle-income countries. Poor mental health during adolescence presents a serious public health concern as it is a known predictor of persistent mental disorders in adulthood. This study evaluates the effectiveness of a new group-based intervention developed by the World Health Organization (Early Adolescent Skills for Emotions; EASE), implemented by non-specialists, to reduce young adolescents’ psychological distress among mostly Syrian refugees in Lebanon.

**Methods:**

We conducted a two-arm, single-blind, individually randomized group treatment trial. Adolescents aged 10 to 14 years who screened positive for psychological distress using the Pediatric Symptom Checklist (PSC) were randomly allocated to EASE or enhanced treatment as usual (ETAU) (1:1.6). ETAU consisted of a single scripted psycho-education home-visit session with the adolescent and their caregivers. EASE consists of seven group sessions with adolescents and three sessions with caregivers. The primary outcome was adolescent-reported psychological distress as measured with the PSC (internalizing, externalizing, and attentional symptoms). Secondary outcomes included depression, posttraumatic stress, well-being, functioning, and caregivers’ parenting and distress. All outcomes were assessed at baseline, endline, and 3 months (primary time point) and 12 months follow-up.

**Results:**

Due to the COVID-19 pandemic and other adversities in Lebanon at the time of this research, the study was prematurely terminated, resulting in an under-powered trial sample (*n* = 198 enrolled compared to *n* = 445 targeted). We screened 604 children for eligibility. The 198 enrolled adolescents were assigned to EASE (*n* = 80) and ETAU (*n* = 118), with retention rates between 76.1 and 88.4% across all timepoints. Intent-to-treat analyses demonstrated no between-group differences on any of the outcome measures between the EASE and ETAU. We did observe a significant improvement on the primary outcome equally in the EASE and ETAU groups (−0.90, 95% CI: −3.6, 1.8; *p* = .52), – a trend that was sustained at three months follow-up. Sub-group analyses, for those with higher depression symptoms at baseline, showed ETAU outperformed EASE on reducing depression symptoms (difference in mean change = 2.7, 95% CI: 0.1, 5.3; *p* = .04; *d* = 0.59) and internalizing problems (difference in mean change 1.0, 95% CI: 0.08, 1.9; *p* = .03; *d* = 0.56) .

**Conclusion:**

No conclusions can be drawn about the comparative effectiveness of the intervention given that the sample was underpowered as a result of early termination. Both EASE and single session psycho-education home visits resulted in meaningful improvements in reducing psychological distress. We did not identify any indications in the data suggesting that EASE was more effective than a single session family intervention in the context of the COVID-19 pandemic and other crises in Lebanon. Fully powered research is needed to evaluate the effectiveness of EASE.

## 1. Introduction

Millions of children and adolescents are forcibly displaced, with armed conflict a major driver and children and adolescents (aged 0–17) account for 40% of all forcibly displaced people [1]. Child and adolescent refugees and asylum seekers have high rates of mental disorders [2] and high rates of exposure to potentially traumatic events, as well as ongoing daily stressors such as poverty, restricted access to livelihoods, barriers to accessing basic services including education and health care, structural and community discrimination, and increases in community and family violence. These present significant risks to healthy development and positive wellbeing [3]. The majority of refugees are hosted in low- and middle-income countries (LMICs), where there are limited resources, with drastically under-funded and under-resourced health systems, and too few mental health professionals to provide services to people with mental health care needs [4].

In order to respond to the increased mental health needs, and contribute to closing the treatment gap, evidence-based and scalable psychological interventions for children and adolescents that can be delivered by non-specialists are needed. The current evidence-base concerning psychological interventions for children and adolescents in humanitarian and LMIC settings is limited [5,6]. A recent individual patient data meta-analysis of psychological support for children in low resource humanitarian settings, from 3143 children (aged 7–18) recruited to 11 randomized controlled trials, found beneficial effects for functioning, coping, hope, social support, and PTSD [7]. However, no effects were found for depression and anxiety and when looking at subgroups, effects were greater for non-displaced and older adolescents (15–18 years). Similarly, a meta-analysis of psychological interventions for refugees and asylum seekers [8] and an umbrella review of psychological interventions in LMICs [6] have found limited and mixed evidence for interventions with children and young adolescents.

Recognizing the need for effective treatment options for young adolescent distress, the World Health Organization (WHO) developed the Early Adolescent Skills for Emotions (EASE) intervention to reduce psychological distress for adolescents aged 10–14 years living in adversity and experiencing internalizing symptoms (e.g. anxiety or depression) [9]. EASE is designed to be applicable across common mental disorders (i.e., transdiagnostic), brief, and feasible for delivery by non-specialists, reducing the reliance on diagnostic specialist services which may not be available. It consists of manualized evidence-based techniques found to be common in effective psychological interventions for this age group globally, and deemed safe and feasible for delivery by trained non-specialists. In order to ensure effectiveness and applicability across a range of settings, a series of pilot and definitive trials were planned in Jordan, Lebanon, Pakistan, and Tanzania [10,11], with a systematic cultural and contextual adaptation conducted in each site.

The research on EASE in Lebanon consisted of: (i) conducting a comprehensive contextual and cultural adaptation of EASE, grounded in findings from a desk review, qualitative research, and cognitive interviewing workshops [12]; (ii) conducting a pilot RCT and process evaluation to determine feasibility of the intervention and research procedures (Brown et al., under review); and (iii) a fully powered RCT, outlined in this paper. This work was conducted as part of the STRENGTHS project, which aims to evaluate the effectiveness and scale-up of psychological interventions for Syrian refugees [13]. The planned aim of this study was to evaluate the effectiveness of EASE for Syrian refugee young adolescents living in Lebanon. The primary hypothesis was that participating in EASE would result in significantly larger reductions in psychological distress when compared to enhanced treatment as usual (ETAU). Due to the COVID-19 pandemic and other adversities (i.e. extreme economic crisis and hardship, followed by nation-wide protests) in Lebanon during the study period, the decision was made to prematurely terminate the trial. Having enrolled only 44% of the intended sample in the trial, the revised aim of the study is exploratory in nature.

## 2. Methods

### 2.1. Design

This two-arm, single-blind individually randomized group treatment trial was conducted in Lebanon to test our hypothesis that participation in EASE results in better outcomes compared to ETAU control condition. Outcomes were assessed at baseline (T0), post-intervention (T1), 3-months follow-up (T2) and 12-months follow-up (T3), with 3-months post intervention as the primary time point for testing the aforementioned hypothesis. We selected the 3 months follow-up as the primary outcome time point because we were primarily interested to investigate whether any treatment benefits would sustain after termination of the intervention. The study was implemented by War Child, an international humanitarian organization that operates in 15 countries to improve the resilience and wellbeing of children affected by armed conflict, and has been responding to the Syrian crisis in Lebanon since 2012. The research was prospectively registered on 11 March 2019 (ISRCTN75375136). The trial protocol has been previously published [10].

### 2.2. Setting

The study was implemented in the north of Lebanon (Akkar and North governorates), an area where large numbers of Syrian and Palestinian refugees reside. Lebanon has experienced prolonged conflict, political instability, and economic crises. Especially in the north of Lebanon this has resulted in high levels of poverty and scarcely available services. >1.5 million Syrian refugees reside in Lebanon, many since the onset of the Syria crisis in 2011, and experience significant adversity as a result of the Syrian conflict, forced migration, and ongoing daily stressors. Syrian refugees living in Lebanon have restricted work rights, tightened in recent years, and do not have permanent status [14]. This state of protracted displacement leads to additional risk factors for their mental health and wellbeing including lack of adequate housing, inconsistent livelihoods, limited access to health and education services.

### 2.3. Changed situation

As our trial was underway from June 2019 onwards, Lebanon experienced an unprecedented financial crisis from which it has yet to emerge. The local currency was severely devalued, resulting in exacerbated economic hardship for Syrian and low-income Lebanese families, and difficulty meeting daily needs. Following the economic hardship and political instability, protests erupted across the country (October 2019), resulting in severe restrictions in movement and an increasingly volatile situation – also in the study area. Just before mid-way through the study, the COVID-19 pandemic struck Lebanon, leading to a national lockdown (21 March 2020). All in-person activities conducted by War Child stopped following nationwide restrictions, which lasted for over 12 months. At that point in time 44% of the intended sample had completed the intervention and post-intervention assessments (T1). For the remainder of the sample recruitment, screening and baseline assessments were being conducted, but the intervention could not be offered due to the lockdown, except administering (phone-based) follow-up interviews (T2, T3) for the 44% of the sample [15]. Due to logistical and financial reasons, restarting the study was not possible after approximately 12 months of COVID-19-restrictions, and changing the intervention to online format was deemed infeasible and introduced too much risk for study participants due to lack of privacy. As a result, the study was terminated, and the results of the 198 adolescents for whom assessments were completed are reported (see details below).

### 2.4. Sample

Participants were recruited into the study through several engagement activities, which included in-person community awareness sessions, communicating about the study through social media channels and through informing participants of NGO and United Nations programs, using scripted information to minimize risk of bias in recruitment. Participants were enrolled in the trial if they met inclusion criteria: (a) aged 10 to 14 years; (b) resided with a related caregiver who could legally provide consent; (c) willing to participate in weekly EASE or ETAU sessions; and (d) demonstrating elevated levels of psychological distress measured with the Pediatric Symptom Checklist (PSC-17) [16]. An optimal clinical cut-off point of 12 was established following a validation against a semi-structured interview by a mental health professional identifying the presence of any psychological disorder requiring treatment (Brown et al., under review). For all eligible young adolescents, one of their caregivers was also invited, and required to consent, to participate in the study. Exclusion criteria were: (a) unaccompanied minor; (b) minor with an unrelated caregiver; (c) significant developmental, cognitive, or neurological impairments as determined by 4 items from an adapted version of the 10 Questions instrument [17]; (d) currently married, and; (d) imminent risk of suicide measured using a 3-item questionnaire based on WHO’s mhGAP Intervention Guide [18]. Any potential participants reporting imminent risk of suicide were referred to relevant services. In case of multiple siblings meeting eligibility criteria, all were included in the study for ethical considerations, but randomized as a single unit to avoid children from the same family being allocated to different treatment conditions.

### 2.5. Randomization and masking

Following baseline assessments, participants were randomly allocated to receive either EASE or ETAU (following a 1:1.6 ratio). Randomization was done by volunteers in the Lebanon office, using computer generated sequences, and putting allocations in numbered opaque envelopes. Stratification was applied for gender and sibling pairs. Block sizes of 13 were used to maintain the randomization ratio at all stages. Research assistants and lead investigators were masked to treatment condition allocation. Interaction between research assistant and intervention facilitators was avoided, and before each round of assessments research assistants instructed participants to not inform them about their allocation. Following each assessment research assistants guessed the allocation of the participant, to assess to what degree masking was maintained.

### 2.6. Interventions

EASE consists of seven sessions for young adolescents and three sessions for their caregivers, and was adapted to the cultural context (Brown et al., 2020). The sessions for adolescents involved; psychoeducation about the effects of stressful events, and identifications of emotions (session 1); reducing arousal using relaxation and stress management techniques (i.e. slow breathing) (session 2); behavioral activation strategies (sessions 3 and 4); problem solving strategies, including seeking social support (session 5 and 6); relapse prevention to manage future stressors (session 7). The caregiver sessions involved; psychoeducation and skills to help their child cope with distress (session 1); positive parenting skills, including praise, reducing harsh punishment and promoting communication skills (session 2); strategies to manage caregivers’ own stress (e.g. advice about sleep, nutrition, stress reduction exercises, and utilization of social support) (session 3). During caregiver sessions, another staff member was available to provide childcare as necessary. Adolescent and caregiver sessions were meant for groups of 6–10 people, and lasted 1.5–2 h each, provided on a weekly basis by two EASE facilitators. EASE facilitators were non-mental health professionals (55% Bachelor degree holders) recruited from the communities where the study took place. The group of trained facilitators (91% women; 45% Syrian, 45% Lebanese, 10% Palestinian) had on average 6.1 years of experience in working with children. Facilitators received an eight-day training in the delivery of the interventions, as well as basic counselling and communication skills, group facilitation, child protection, security, and self-care. Following the training, all facilitators completed a supervised practice cycle of EASE ahead of the study implementation. During the study, weekly group-based supervision was offered.

The ETAU condition consisted of a single-session psycho-education conducted during a home visit. This psycho-education session was offered because treatment as usual in the study settings mostly entailed no treatment at all, given the limited availability of mental health services. Both the caregiver and adolescent were invited to participate in the session, which lasted 30–45 min. The scripted session consisted of: (i) sharing the screening results to the family; (ii) discussing some common and simple to use self-care strategies to manage high levels of distress, and; (iii) information about the few available mental health and psychosocial support services provided by international NGOs. ETAU facilitators, recruited following the same criteria and process as the EASE facilitators, received a 3-day training in the delivery of the psycho-education session; otherwise the training was content-matched between both groups. Only one supervision session was offered during the implementation period.

### 2.7. Instruments

#### 2.7.1. Primary outcome

The primary outcome is psychological distress at three-month follow up, measured using the Pediatric Symptom Checklist (PSC-35), a 35-item adolescent self-report instrument scored on a 3-point response scale (0 = never, 2 = often; range of total score 0–70), with a higher total score indicating more severe problems [16]. The instrument has been validated for use in Lebanon, demonstrating good internal consistency (α = 0.80) (Brown et al., under review). As 17 items of the instrument were used during the screening (see above), the remaining 18 items were asked during the baseline interviews which were conducted within 14 days of the screening interview. The PSC-35 has three sub-scales for internalizing- (5 items, range 0–10; α = 0.43), externalizing- (7 items, range 0–14; α = 0.25), and attention- (5 items, range 0–10; α = 0.23) problems (the internalizing sub-scale total score was used as a secondary outcome). The timeframe for the questions in this instrument, as well as for the other instruments described below, was two weeks.

#### 2.7.2. Secondary outcome

Several secondary outcomes were also completed by adolescents. Symptoms of depression in the last week were assessed using the Patient Health Questionnaire, adolescent version (PHQ-A) [19], a nine-item measure scored on a four-point scale (0 = not at all, 3 = nearly every day), with a total score ranging between 0 and 36 (α = 0.83). Higher total scores reflect more severe symptoms of depression. Symptoms of traumatic stress were assessed using the Children’s Revised Impact of Events Scale (CRIES), a 13-item instrument with items scored on a five-point scale (0 = not at all, 4 = often), with a higher total score (0–65) indicating more severe symptoms (α = 0.88) [20]. The CRIES has three subscales, intrusion (four items), avoidance (four items), and arousal (five items). The Daily Functioning instrument has been developed in Lebanon, specifically for this study, replicating a procedure developed by Bolton and colleagues [21]. The nine-item instrument aims to assess impairment in daily activities, which adolescents had identified and rated as most relevant for them as part of the tool development process (α = 0.82). Scoring is done one a 4-item response scale (0 = not at all, 3 = very much), with a higher total score (0–27) indicating higher perceived impairment in daily functioning. The 14-item Warwick Edinburgh Mental Wellbeing Scale (WEMWBS) assesses wellbeing [22], with respondents asked to indicate which score best describes their thoughts or feelings over the past week on a scale from 1 (none of the time) to 5 (all of the time) (α = 0.85). A higher total score (14–70) indicates greater positive mental wellbeing.

Several additional secondary outcomes were completed by the caregivers. The caregiver version of the PSC-35 to assess their perceptions of the psychological distress of their child is identical to the child version described above (α = 0.83). We used the Kessler Psychological Distress Scale (K6), a six-item measure of distress symptoms experienced in the past week [23]. Scoring is done on a five-point scale (1 = all of the time, 5 = none of the time), with a higher total score (6–30) indicating more distress (α = 0.73). The Alabama Parenting Questionnaire (APQ-42) was used to assess parenting behaviors [24]. The 42-item instrument measures 5 major parenting constructs: (a) parental involvement (10 items) (α = 0.69); (b) poor supervision and monitoring (10 items) (α = 0.65); (c) positive parenting (6 items) (α = 0.65); (d) inconsistent discipline (6 items) (α = 0.39); and (e) corporal punishment (3 items) (α = 0.53). Scoring follows a five-point scale (1 = never, 5 = always).

#### 2.7.3. Other measures

To assess adolescents’ exposure to potentially traumatic events, caregivers completed a 26-item traumatic events checklist, which was adapted from existing trauma exposure checklists [25,26]. Items were scored dichotomously. Both adolescents and caregivers completed a Strategies Use Questionnaire (seven and eight items, respectively), developed for the purpose of this study in order to assess the use of coping strategies. The selected strategies map onto the content of the EASE intervention, but formulated so that it can be scored independent of knowledge or experience of EASE and to maintain masking, in order to gauge the degree by which participants adopt strategies underlying the EASE intervention. This mechanisms of action tool was included as a hypothesized mediator of change. Items are scored on a five-item response scale (0 = never; 4 = all of the time). A higher total score (0–28 for adolescents and 0–32 for caregivers), indicate higher degree of use of strategies (α = 0.73 and α = 0.59, respectively). Fidelity was assessed by each pair of facilitators completing a session checklist, developed specifically for the purpose of this study, following each EASE or ETAU session. A sample of approximately 10% of the EASE and ETAU sessions was observed by a trained staff member, to complete a structured observation form to assess fidelity.

#### 2.7.4. Administration

All instruments were translated into simple Arabic understandable to children living in Lebanon, details of translation are detailed elsewhere [10,27]. All instruments were interview administered by trained research assistants, using Kobo electronic data collection software on tablets (https://kobo.humanitarianresponse.info/). Research assistants received a five-day training in basic research skills, interviewing techniques, consent-, adverse events reporting-, and ethics-procedures, and practicing the administration of research instruments.

### 2.8. Analysis

All analyses were conducted in Stata, version 15 [28]. We calculated descriptive statistics (means, standard deviations, percentages) to summarize baseline characteristics of the study sample separately by trial arm for both adolescents and caregivers. We estimated linear mixed effects models to estimate the difference in change from baseline to each follow-up timepoint (endline, 3-month, 12-month follow-up) between EASE and ETAU separately for each outcome. Fixed effects included treatment arm, time (0 = baseline; 1 = endline; 2 = 3-month follow-up; 3 = 12-month follow-up) and interaction terms between treatment arm and time. Random effects included participant ID, family ID (to account for the fact that some adolescents had siblings in the study), and intervention group (EASE). For models with caregiver-reported data, when each observation was unique for a child (e.g., PSC), all observations were included (*n* = 198). When caregivers only reported once regardless of the number of children they had in the study, we included 155 observations (the number of unique caregivers) so that caregivers with multiple children in the study were not duplicated in the model (e.g., K6, Alabama outcomes). For these latter outcome models, random effects included Caregiver ID and intervention group.

For each outcome, we report predicted means at baseline and each follow-up for both groups from the mixed effects model, as well as the estimated difference in mean change in outcome score between groups at each timepoint with 95% confidence intervals. We calculated Cohen’s *d* effect size as the difference in mean change divided by the pooled baseline standard deviation.

We ran post-hoc analyses to assess the response rates for depression and internalizing symptoms (defined as at least 50% reduction in PHQ-9 and score PSC Internalizing subscale compared with baseline). Missing data was assumed to be missing at random and addressed through multiple imputation with chained equations.

We also conducted a series of exploratory post hoc subgroup analyses for the following outcomes: PSC, PSC Internalizing, PSC Externalizing, PHQ, Wellbeing, and Caregiver PSC. For those outcomes, we re-estimated the above-described models separately within strata of the following characteristics: gender (males and females), age (10–12 year old and 13–14 year old), treatment status (completers and non-completers) and symptom severity (top and bottom 50% of baseline scores on PSC, PSC Internalizing, PSC Externalizing, PHQ, Wellbeing, Caregiver PSC, and K6). Models were identical to those in the main analysis except that we only analyzed difference in mean change for one follow-up timepoint (endline).

Sample size calculations took into account that for an individually randomized group-treatment trial it is expected that there will be clustering in the EASE arm due to the group-based delivery of the intervention. The power analyses accounted for this clustering and the potential inflation of outcome variance in the EASE intervention arm, resulting in an allocation ratio of EASE to ETAU arms of 1:1.6 [29]. We used a conservative estimate of theta (ratio of variances) of 1.1 and of the intraclass correlation as 0.13. Assuming a 5% two-tailed significance test and 80% power, it was estimated that data from 445 participants are needed to be available at the 3-month follow-up to detect an effect size of 0.4, allowing for 30% loss to follow up.

### 2.9. Ethics

This study is reported as per the Consolidated Standards of Reporting Trials (CONSORT) guideline (S1 Consort Checklist). Ethical approval for the conduct of this study was obtained from St Joseph University (USJ.201724) and the World Health Organization Ethical Review Committee (ERC.0003000). We followed a two-staged process to obtain informed written consent from caregivers and assent from the young adolescents. First, to participate in the screening; second, for those meeting inclusion criteria to participate in the trial. Witnessed oral consent was accepted for illiterate participants.

## 3. Results

**Fig. 1.**
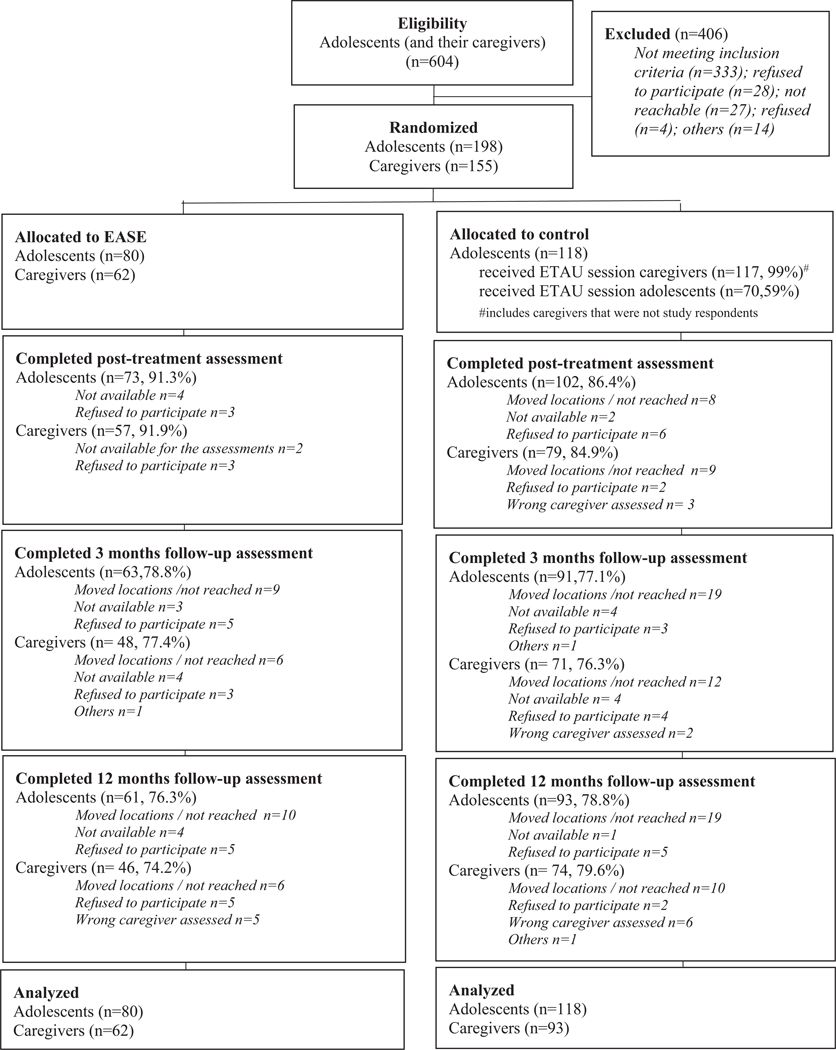
CONSORT flow diagram.

Study enrollment started on 19 June 2019 and the final 12-months follow-up assessment was done on 8 March 2021. From 604 adolescents that were screened, 198 (33%) met inclusion criteria, attended baseline and were randomly allocated to EASE (*n* = 80) and ETAU (*n* = 118). The retention rates were within the margin that was projected, 88.4% for adolescents at endline, at both 3- and 12-months follow up this was 77.8%. For caregivers, the retention rates were similar, 87.7% at endline, 76.1% at 3-months, and 78.8% at 12-months follow-up. See Fig. 1 for the full overview of participant recruitment, retention and reasons for drop-out. The unbalanced sample size in each arm is the result of the randomization process following a 1:1.6 allocation ratio.

Sample characteristics are summarized in Table 1 (adolescents) and Table 2 (caregivers). Adolescents were 51% male and the average age was 11.8 (SD = 1.3). Ninety-seven percent (*n* = 192) were born in Syria and the mean number of trauma types experienced was 6.9 (SD = 3.9). The most frequently reported were: having been in danger during flight (71%); lack of food and water (68%); and witnessing serious accident, fire or explosion (62%). The majority of caregivers were mothers (83.2%; *n* = 129) and the average caregiver age was 38.4 (SD = 8.0). There were no meaningful differences in baseline demographic characteristics between EASE and ETAU groups.

Adolescents allocated to the treatment arm were divided over 11 EASE groups, and caregivers over 8 groups. Adolescents attended an average of 4.6 sessions, with 70.0% (*n* = 56) attending 5 sessions or more (a priori established as ‘completers’). Further, 13.8% did not attend any of the EASE sessions. Caregivers attended on average 1.5 sessions, and 48% (*n* = 30) attended at least 2 sessions. Further, 12% of all EASE sessions (adolescent and caregivers combined) were supervisor-observed for assessment of intervention fidelity. For adolescent and caregiver sessions combined, 96% of fidelity items were assessed to be satisfactory (62% done well, 34% done partially). For adolescent sessions alone the results were similar (95% overall, with 57% done well, and 38% done partially).

The primary and secondary outcomes for adolescents and caregivers are provided in Tables 3 and 4, respectively. For the primary outcome, PSC (adolescent report), there were significant reductions in mean score from baseline to both endline and 3-month follow-up within both groups; by 12 months, mean scores had rebounded close to the baseline level in both groups. A similar trend, though less pronounced, is seen on the PHQ. There was no statistically significant difference in mean change between groups at 3-months follow-up, the primary endpoint (−0.53, 95% CI: −3.4, 2.3; *p* = .71) or any other timepoint. Further, for EASE and ETAU we see a response rate of 23.8 (95% CI: 13.1, 34.4) and 21.5 (95% CI: 12.8, 30.1) on PHQ, and 35.7 (95% CI: 22.3, 49.0) and 37.5 (95% CI: 26.3, 48.7), respectively on PSC internalizing problems; these between-group differences were not statistically significant (risk ratio 1.11 [95% 0.44, 1.78], *p* = .74; and 0.95 [95% 0.50, 1.4], *p* = .94, respectively). There were also no significant differences in mean change between groups for any other outcome (adolescent or caregiver).

All sub-group analyses are included in Supplemental File 1; in the main text the results on the PSC and PHQ are presented because of the aforementioned within-group changes on these outcomes. Table 5 presents outcomes among adolescents in the top 50% of baseline PSC scores and Table 6 presents outcomes among adolescents in the top 50% of PHQ scores. Among adolescents with the top 50% of PHQ scores at baseline, the ETAU group had a significantly greater reduction in PHQ scores from baseline to endline compared to the EASE group (difference in mean change = 2.7, 95% CI: 0.1, 5.3; *p* = .04; d = 0.59). Similarly, among adolescents in the top 50% of PHQ baseline scores, PSC Internalizing scores had a significantly greater reduction among ETAU than EASE (difference in mean change 1.0, 95% CI: 0.08, 1.9; *p* = .03; *d* = 0.56). And reversely, among adolescents with the bottom 50% of PHQ scores at baseline, the EASE group had a significantly greater reduction in PHQ scores compared to ETAU (difference in mean change −2.7, 95% CI: −5.0, −0.42; *p* = .02; *d* = 0.93), and greater reduction in PSC Internalizing scores (difference in mean change −0.98, 95% CI: −2.0, 0.02; *p* = .05; *d* = 0.49). During the entire trial period, 17 severe adverse events were reported. The Data Safety Management Committee reviewed and approved the actions that were taken for all of these cases.

Results from the assessment of the degree to which masking to the group allocation was maintained demonstrate that 56.0% of research assistants conducting interviews with adolescents correctly guessed group allocation, and 49.4% for the interviews with caregivers, which is close to chance level. Only one research assistant indicated to be ‘somewhat sure’ about the answer, the rest indicated to have simply guessed. Analyses of the pattern of reported cases to and follow-up by the focal point for reported child protection and adverse events issues (i. e. hotline or case-management referrals), demonstrated 31% among the EASE participants and 32% among the ETAU participants, with slightly higher case-management referrals in the latter group (21% vs 16%).

## 4. Discussion

As a result of COVID-19 and an unprecedented combination of adversities in Lebanon at the time of this research, the trial was terminated prematurely, resulting in an under-powered trial sample of 198 adolescents (44% of the 445 that were targeted). This makes it not possible to draw conclusions on comparative effectiveness of the intervention versus the control group. Still, the sample was large enough to conduct useful exploratory analyses. These did not identify between-group differences on any outcome measure between the experimental and control groups. We observed significant within-group improvements on the primary outcome, psychological distress and internalizing problems, both in the EASE and ETAU groups – sustained at 3 months post intervention and largely lost at 12 months. These improvements appear clinically relevant with a response rate for internalizing problems between 36% and 38% (EASE vs ETAU) and for depression specifically between 24% and 22%, respectively. The change on the primary outcome (but not on any of the other outcomes) is congruent with what was found in the twin trial evaluating EASE in Jordan [30], with the difference that in our study the control condition had similar improvement. Though not powered, post hoc sub-group analyses suggest that the control condition may outperform EASE on psychological distress and internalizing problems for the subgroup with higher distress; and EASE may outperform the control groups on the same outcomes for the subgroup with lower distress. The lack of sustained improvements for internalizing problems at 12 months is consistent with other studies evaluating a similar intervention for Syrian refugee adults in Jordan (Bryant et al., 2022), and can also be explained by the high levels of stress experienced as a result of the continuing and aggravating economic hardship in Lebanon (Miller et al., 2022).

It appears that EASE does have the potential to result in reducing psychological distress, as intended [9] (though no other improvements were shown), however to a similar degree as the single psychoeducation session, which possibly does so even better for those experiencing higher distress at baseline. Explaining this as a regression to the mean is unlikely given that none of the other indicators show a significant change over time. Instead, in case these findings would be confirmed in a fully powered trial using the same design in Lebanon, we hypothesize that (i) the control condition may have been too active as a comparator, (ii) EASE may have been implemented in a way that did not allow it to demonstrate full potential in the Lebanon context, and/or (iii) EASE is not effective.

First, the similar change on the primary outcome reveals the possibility that participation in the single session family psycho-education home visits has resulted in more improvement than expected. Even if the sessions were scripted and time-limited, it still entailed individualized attention in a private setting, compared to the group sessions of EASE. The home-visit might have been particularly beneficial as it allowed for contact with and personal attention to the family, as well as detection of adverse events and dedicated attention to referral options. These home visits may have been perceived as especially caring and supportive. Also, because it concerned home-visits, scheduling was easier allowing for high levels of attendance among control group participants (compared to relatively low attendance among EASE groups). The first hypothesis on the potency of a single home-visit session is supported by evidence from a meta-analyses including 50 trials showing that single session interventions outperform control groups by 58%, especially for anxiety and conduct problems [31]. Also for adolescents with depression and anxiety a single growth mindset session demonstrated effectiveness in reducing internalizing distress [32]. In humanitarian and LMIC contexts where resources are scarce, barriers to accessing mental health care pronounced, and drop-outs common, the potential for scalable single session in-person interventions should be further explored [33].

Second, the hypothesis that EASE did not yield its full potential is based on (i) an average of 2.4 sessions of non-attendance, as well as 13.8% of children receiving nil sessions of EASE, and (ii) not seeing any significant before to after change in the utilization of strategies that map onto the content and hypothesized mechanisms of action of EASE. For an intervention to be effective, one expects a change on the exact strategies that the intervention aims to instill in participants. For example, in a trial in Nepal evaluating the adult-version of EASE, group PM+, a significant increase in use of intervention specific strategies explain up to 33% of the treatment effects [34]. It raises the question whether young adolescents, especially when experiencing severe adversity can genuinely adopt strategies in a group format and apply them to their own life. Equally the intervention may not have been delivered optimally, with 62% of the strategies observed as delivered well. This may also explain the mixed results from trials evaluating psychological interventions for children with depression and anxiety [6].

While interventions for PTSD and conduct problems among adolescents in adversity have shown promising results [6,7], there is still a serious need for interventions addressing depression and anxiety, as these conditions make up most of the burden of disease among adolescents [35]. Based on results from the trial in Jordan, EASE shows potential in reducing emotional distress [30]. As the improvements on emotional distress among the EASE participants in our underpowered study is similar to that of the control group, fully powered trials on the effectiveness of EASE are needed. Furthermore, research should investigate if EASE seems to only result in changes in internalizing problems [30], and no other intended outcomes, and explain why this may be the case. Finally, while unintended, this study raises the question once again about the potential of single session interventions as a (cost-)effective alternative [31]. In addition, our results may speak to the potency of working with whole families through home visits, which is supported by recent review concluding that family-based mental health interventions, including home visits, are potentially effective for refugees [36].

A few limitations need to be reported. First and foremost, the study was underpowered by >50%. As noted, premature termination of the trial was the result of an unprecedented combination of adverse events, including COVID-19 restrictions, dramatic economic downfall, political instability, and violent demonstrations, which made continuation of the study impossible. This may also have influenced the results among the sample that is included in this paper. At the same time, it is important to note that interventions such as EASE have been developed by the WHO especially for situations of adversity and humanitarian crises. Future implementation science studies should investigate how to ensure continuity of services in unstable contexts. Second, the study raises questions about what the optimal control condition is in settings where essentially no treatment as usual exists, and not offering anything is deemed unethical. To account for the potential effect of adding an active component to the control condition the experimental group could be offered the same as the ETAU condition, a strategy that was applied in the above-mentioned evaluation of Group PM+ in Nepal [34]. The downside is that one does not test EASE in isolation, but only in combination with the brief psychoeducation session for example. This dilemma continues to deserve attention, given that meta-analysis have shown that the selection of control condition affects the results of psychotherapy trials for depression [37,38].

### 4.1. Conclusion

In sum, no conclusions can be drawn about the comparative effectiveness of EASE given that the sample was underpowered as a result of early termination. Both EASE and single session psycho-education home visits resulted in meaningful improvements in reducing psychological distress. We did not identify any indications in the data suggesting that EASE was more effective than a single session family intervention in the context of the COVID-19 pandemic and other crises in Lebanon. Fully powered research is needed to evaluate the effectiveness of EASE.

## Supplementary Material

Supplementary Materials

## Figures and Tables

**Table 1:** Baseline adolescent demographic characteristics.

	Total (n = 198)	EASE (*n* = 80)	ETAU (*n* = 118)
	
	N (%) or Mean (SD) [Range]		

Female	97 (49.0)	37 (46.3)	60 (50.9)
Male	101 (51.0)	43 (53.8)	58 (49.1)
Age*	11.8 (1.3)	11.7 (1.3)	11.9 (1.3)
	[10–14]	[10–14]	[10–14]
Country of Birth*			
Lebanon	5 (2.5)	2 (2.5)	3 (2.5)
Syria	192 (97.0)	77 (96.3)	115 (97.5)
Missing	1 (0.5)	1 (1.3)	0 (0.0)
Birth order of child*			
1	56 (28.3)	24 (30.0)	32 (27.1)
2	32 (16.2)	15 (18.8)	17 (14.4)
3	35 (17.7)	12 (15.0)	23 (19.5)
4	26 (13.2)	9 (11.3)	17 (14.4)
5	20 (10.1)	8 (10.0)	12 (10.2)
6+	28 (14.1)	11 (13.8)	17 (14.4)
Missing	1 (0.5)	1 (1.3)	0 (0.0)
Child currently in school*			
Yes, formal education	101 (51.0)	47 (58.8)	54 (45.8)
Yes, informal education	13 (6.6)	6 (7.5)	7 (5.9)
No	83 (41.9)	26 (32.5)	57 (48.3)
Missing	1 (0.5)	1 (1.3)	0 (0.0)
Child ever been in school (among those not currently in school)*			
Yes	64 (77.1)	19 (73.1)	45 (79.0)
No	19 (22.9)	7 (26.9)	12 (21.0)
Child brings income to the family*			
Yes	81 (40.9)	37 (46.3)	44 (37.3)
No	116 (58.6)	42 (52.5)	74 (62.7)
Missing	1 (0.5)	1 (1.3)	0 (0.0)
Child helps with childcare*			
Yes	98 (49.5)	42 (52.5)	56 (47.5)
No	99 (50.0)	37 (46.3)	62 (52.5)
Missing	12 (0.5)	1 (1.3)	0 (0.0)
Participated in other WC/NGO activities in past month			
Yes	12 (6.1)	5 (6.3)	7 (5.9)
No	186 (93.9)	75 (93.7)	111 (94.1)
Mean number of trauma types	6.9 (3.9)	7.1 (4.0)	6.8 (3.9)
experienced	[0–17]	[0–17]	[0–15]

* Denotes caregiver report.

**Table 2:** Baseline caregiver demographic characteristics.

	Total (*n* = 155)	EASE (*n* = 62)	ETAU (*n* = 93)
	
	N (%) or Mean (SD) [Range]		

Age	38.4 (8.0) [18–65]	38.0 (7.4) [18–51]	38.8 (8.2) [18–65)
Caregiver type			
Mother	129 (83.2)	52 (83.9)	77 (82.8)
Father	19 (12.3)	8 (12.9)	11 (8.3)
Other family member	7 (4.5)	2 (3.2)	5 (5.4)
Mother in the household			
Yes	147 (94.8)	58 (93.5)	89 (95.7)
No	8 (5.2)	4 (6.5)	4 (4.3)
Father in the household			
Yes	126 (81.3)	50 (80.7)	76 (81.7)
No	29 (18.7)	12 (19.3)	17 (18.3)
Mother’s education			
No school	52 (33.6)	20 (32.3)	32 (34.4)
Primary school	47 (30.3)	20 (32.3)	27 (29.0)
Middle school	46 (29.7)	18 (29.0)	28 (30.1)
High school	1 (0.7)	0 (0.0)	1 (1.1)
Higher education	0 (0.0)	0 (0.0)	0 (0.0)
Other	4 (2.6)	2 (3.2)	2 (2.2)
Missing	5 (3.2)	2 (3.2)	3 (3.2)
Father’s education			
No school	22 (14.2)	4 (6.5)	18 (19.4)
Primary school	40 (25.8)	20 (30.7)	20 (21.5)
Middle school	61 (39.4)	23 (38.7)	38 (40.9)
High school	1 (0.7)	1 (1.6)	0 (0.0)
Higher education	2 (1.3)	2 (3.2)	0 (0.0)
Other	1 (0.7)	1 (1.6)	0 (0.0)
Missing	28 (18.1)	11 (17.7)	17 (18.3)
Housing type			
Informal settlement	131 (84.5)	53 (85.5)	78 (83.9)
Camp	7 (4.5)	3 (4.8)	4 (4.3)
Rented room	6 (3.9)	1 (1.6)	5 (5.4)
Rented house	9 (5.8)	5 (8.1)	4 (4.3)
Owned property	2 (1.3)	0 (0.0)	2 (2.2)
Income			
<$299	123 (79.3)	49 (79.0)	74 (79.6)
$300–$599	31 (20.0)	13 (21.0)	18 (19.4)
$600–$899	1 (0.7)	0 (0.0)	1 (1.1)
Participated in other WC/NGO activities in past month			
Yes	10 (6.5)	3 (4.8)	7 (7.5)
No	145 (93.5)	59 (95.2)	86 (92.5)

**Table 3:** Predicted means, mean changes, and effect sizes for adolescent primary and secondary outcomes.

		Estimated mean (se)				
					
Outcome	Timepoint	EASE (N = 80)	ETAU (N = 118)	Difference in mean change (95% CI)	*p*-value	Cohen’s d effect size

Child psychological symptoms: Total	Baseline	27.4 (1.3)	26.9 (2.6)			
	Endline	22.0 (1.3)	22.5 (2.6)	−0.90 (3.6, 1.8)	0.52	0.11
	3-months	21.6 (1.4)	21.7 (2.6)	−0.53 (−3.4, 2.3)	0.71	0.07
	12-months	26.0 (1.4)	25.6 (2.6)	−0.01 (−2.9, 2.9)	0.99	0.001
Child psychological symptoms: Internalizing	Baseline	5.3 (0.3)	5.3 (0.5)			
	Endline	3.8 (0.3)	3.8 (0.5)	0.01 (−0.7, 0.7)	0.97	0.005
	3-months	4.0 (0.3)	4.1 (0.5)	−0.10 (−0.8, 0.6)	0.78	0.05
	12-months	4.4 (0.3)	4.7 (0.5)	−0.30 (−1.1, 0.4)	0.42	0.16
Child psychological symptoms: Attention	Baseline	5.9 (0.3)	5.9 (0.5)			
	Endline	4.2 (0.3)	4.4 (0.5)	−0.3 (−1.1, 0.4)	0.40	0.19
	3-months	4.6 (0.3)	4.5 (0.5)	0.07 (−0.7, 0.8)	0.86	0.04
	12-months	5.1 (0.3)	4.9 (0.5)	0.2 (−0.6, 1.0)	0.60	0.12
Child psychological symptoms: Externalizing	Baseline	3.6 (0.2)	3.7 (0.2)			
	Endline	3.1 (0.3)	2.9 (0.2)	0.3 (−0.4, 1.0)	0.45	0.14
	3-months	3.3 (0.3)	3.3 (0.2)	0.05 (−0.7, 0.8)	0.88	0.03
	12-months	3.6 (0.3)	3.4 (0.2)	0.3 (−0.5, 1.1)	0.48	0.15
PHQ	Baseline	9.5 (0.8)	9.6 (1.4)			
	Endline	8.3 (0.8)	8.3 (1.4)	0.10 (−1.7, 1.9)	0.92	0.02
	3-months	7.4 (0.9)	8.3 (1.4)	−0.8 (−2.8, 1.1)	0.39	0.13
	12-months	9.7 (0.8)	10.7 (1.4)	0.9 (−2.8, 1.0)	0.35	0.14
WEBWBS	Baseline	44.7 (1.2)	44.3 (1.0)			
	Endline	45.5 (1.2)	44.0 (1.0)	1.1 (−2.3, 4.6)	0.52	0.10
	3-months	44.6 (1.2)	43.8 (1.1)	0.4 (−3.2, 3.9)	0.84	0.03
	12-months	42.7 (1.3)	41.3 (1.1)	1.1 (−2.4, 4.6)	0.55	0.10
CRIES	Baseline	26.1 (2.2)	26.3 (4.0)			
	Endline	26.7 (2.2)	24.8 (4.0)	2.0 (−3.1, 7.2)	0.44	0.12
	3-months	24.9 (2.3)	25.0 (4.1)	0.2 (−5.0, 5.3)	0.95	0.01
	12-months	28.2 (2.4)	27.5 (4.1)	1.0 (−4.4, 6.3)	0.73	0.06
Functioning	Baseline	8.5 (0.9)	8.8 (1.7)			
	Endline	8.0 (0.9)	8.1 (1.7)	0.2 (−2.2, 2.6)	0.85	0.03
	3-months	8.0 (0.9)	8.3 (1.7)	0.1 (−2.5, 2.6)	0.97	0.01
	12-months	8.5 (1.0)	9.2 (1.7)	0.4 (−2.9, 2.1)	0.75	0.06
Use of intervention strategies	Baseline	15.0 (0.6)	14.6 (0.5)			
	Endline	15.2 (0.6)	14.7 (0.5)	0.1 (−2.1, 2.2)	0.96	0.01
	3-months	14.6 (0.7)	13.8 (0.6)	0.3 (−1.8, 2.5)	0.75	0.06
	12-months	14.5 (0.7)	14.6 (0.6)	−0.6 (−2.7, 1.6)	0.60	0.10

Means, SEs, difference in mean change are based on coefficients and combination of coefficients from mixed effects model following multiple imputation.

Cohen’s d effect size was calculated by dividing the predicted difference in mean change from the mixed effects model by the pooled baseline standard deviation.

Model included fixed effects of arm, time and arm X time interaction, and random effects of pt_code, family ID, and EASE group.

**Table 4:** Predicted means, mean changes, and effect sizes for caregiver outcomes.

		Estimated mean (se)				
					
Outcome	Timepoint	EASE (N = 80)	ETAU (N = 118)	Difference in mean change (95% CI)	p-value	Cohen’s d effect size

Caregiver PSC: Total	Baseline	28.5 (1.4)	27.5 (2.6)			
	Endline	27.2 (1.4)	27.6 (2.6)	−1.4 (−4.3, 1.5)	0.36	0.14
	3-months	25.4 (1.4)	26.2 (2.6)	−1.8 (−4.8, 1.1)	0.22	0.01
	12-months	30.2 (1.5)	28.6 (2.6)	0.6 (−2.6, 3.8)	0.71	0.06
Caregiver PSC: Internalizing	Baseline	5.1 (0.3)	5.0 (0.4)			
	Endline	4.8 (0.3)	5.0 (0.4)	−0.3 (−1.0, 0.4)	0.41	0.13
	3-months	5.1 (0.3)	5.1 (0.4)	−0.1 (−0.9, 0.6)	0.73	0.05
	12-months	5.7 (0.3)	5.4 (0.4)	0.2 (−0.6, 1.0)	0.65	0.08
Caregiver PSC: Attention	Baseline	5.6 (0.3)	5.4 (0.5)			
	Endline	5.6 (0.3)	5.4 (0.5)	0.01 (−0.7, 0.7)	0.98	0.005
	3-months	4.9 (0.3)	4.9 (0.5)	−0.2 (−1.0, 0.5)	0.56	0.10
	12-months	5.7 (0.3)	5.3 (0.5)	0.3 (−0.5, 1.1)	0.45	0.14
Caregiver PSC: Externalizing	Baseline	4.6 (0.4)	4.3 (0.8)			
	Endline	4.2 (0.4)	4.1 (0.8)	−0.2 (−1.0, 0.5)	0.54	0.09
	3-months	4.1 (0.4)	4.1 (0.8)	−0.4 (−1.2, 0.4)	0.33	0.14
	12-months	4.9 (0.4)	4.3 (0.8)	0.2 (−0.6, 1.1)	0.60	0.08
K6	Baseline	22.6 (0.7)	23.0 (0.5)			
	Endline	21.9 (0.7)	22.3 (0.6)	−0.01 (−1.9, 1.9)	0.99	<0.001
	3-months	23.0 (0.7)	23.2 (0.6)	0.2 (−1.7, 2.1)	0.83	0.04
	12-months	22.9 (0.7)	23.2 (0.6)	0.1 (−1.9, 2.2)	0.89	0.03
Alabama: Parental Involvement	Baseline	34.2 (1.0)	34.3 (1.0)			
	Endline	32.9 (1.0)	34.2 (1.1)	−1.2 (−4.0, 1.7)	0.42	0.17
	3-months	30.7 (1.0)	31.7 (1.0)	−1.0 (−3.9, 2.0)	0.52	0.13
	12-months	32.3 (1.0)	32.1 (1.1)	0.2 (−2.6, 3.0)	0.90	0.03
Alabama: Positive Parenting	Baseline	24.9 (0.6)	24.7 (0.5)			
	Endline	23.4 (0.6)	23.4 (0.5)	−0.2 (−2.0, 1.6)	0.83	0.05
	3-months	22.0 (0.6)	21.5 (0.5)	0.2 (−1.7, 2.1)	0.83	0.05
	12-months	23.4 (0.6)	23.7 (0.5)	−0.6 (−2.5, 1.3)	0.54	0.14
Alabama: Poor Monitoring	Baseline	16.3 (0.7)	16.4 (0.5)			
	Endline	16.4 (0.7)	14.6 (0.6)	1.9 (−0.7, 3.8)	0.06	0.35
	3-months	15.1 (0.7)	15.4 (0.6)	−0.1 (−2.2, 2.0)	0.92	0.02
	12-months	15.1 (0.7)	15.3 (0.6)	−0.1 (−2.1, 1.9)	0.94	0.01
Alabama: Inconsistent Discipline	Baseline	16.7 (0.5)	17.3 (0.4)			
	Endline	16.8 (0.5)	16.7 (0.4)	0.8 (−0.8, 2.4)	0.34	0.19
	3-months	16.5 (0.6)	16.8 (0.5)	0.3 (−1.6, 2.1)	0.79	0.06
	12-months	17.0 (0.6)	17.5 (0.5)	0.1 (−1.7, 1.8)	0.93	0.02
Alabama: Corporal Punishment	Baseline	7.7 (0.3)	7.9 (0.3)			
	Endline	7.9 (0.4)	7.9 (0.3)	0.3 (−0.7, 1.3)	0.57	0.09
	3-months	7.7 (0.4)	7.8 (0.3)	0.2 (−0.8, 1.2)	0.70	0.06
	12-months	7.8 (0.4)	7.5 (0.3)	0.6 (−0.5, 1.6)	0.29	0.18
Use of intervention strategies	Baseline	19.3 (0.7)	19.4 (1.1)			
	Endline	18.9 (0.7)	19.6 (1.1)	−0.5 (−2.6, 1.6)	0.66	0.09
	3-months	18.1 (0.8)	18.4 (1.1)	−0.1 (−2.3, 2.0)	0.92	0.02
	12-months	18.6 (0.8)	19.2 (1.1)	−0.4 (−2.6, 1.7)	0.70	0.08

Means, SEs, difference in mean change are based on coefficients and combination of coefficients from mixed effects model following multiple imputation.

Cohen’s d effect size was calculated by dividing the predicted difference in mean change from the mixed effects model by the pooled baseline standard deviation.

Model included fixed effects of arm, time and arm X time interaction, and random effects of pt_code, family ID, and EASE group. For all PSC outcomes, which refer to caregiver reports of the child’s symptoms, we included N = 198 observations including caregivers who provided reports on multiple children. For K6, Alabama, and Coping outcomes, which refer to the caregiver’s own symptoms and signs, we only included each caregiver once (N = 155 total; EASE N = 62; ETAU N = 93)

**Table 5:** Predicted means, mean changes, and effect sizes for adolescent outcomes among adolescents with top 50% PSC scores.

		Estimated mean (se)				
					
Outcome	Timepoint	EASE (*N* = 43)	ETAU (*N* = 59)	Difference in mean change (95% CI)	p-value	Cohen’s d effect size

Child psychological symptoms: Total	Baseline	33.3 (1.4)	33.4 (1.2)			
	Endline	27.3 (1.5)	26.6 (1.3)	0.8 (−3.2, 4.8)	0.70	0.13
Child psychological symptoms: Internalizing	Baseline	6.1 (0.3)	6.1 (0.3)			
	Endline	4.8 (0.3)	4.4 (0.3)	0.4 (−0.6, 1.3)	0.42	0.21
Child psychological symptoms: Externalizing	Baseline	4.2 (0.4)	3.9 (0.3)			
	Endline	3.6 (0.4)	3.1 (0.3)	0.2 (−0.8, 1.3)	0.67	0.10
PHQ	Baseline	13.0 (1.0)	13.5 (0.8)			
	Endline	12.0 (1.0)	10.2 (0.9)	2.3 (−0.3, 4.9)	0.08	0.40

Means, SEs, difference in mean change are based on coefficients and combination of coefficients from mixed effects model following multiple imputation.

Cohen’s d effect size was calculated by dividing the predicted difference in mean change from the mixed effects model by the pooled baseline standard deviation.

Model included fixed effects of arm, time and arm X time interaction, and random effects of pt_code, family ID, and EASE group.

**Table 6:** Predicted means, mean changes, and effect sizes for adolescent outcomes among adolescents with top 50% PHQ scores.

		Estimated mean (se)				
					
Outcome	Timepoint	EASE (*N* = 40)	ETAU (*N* = 61)	Difference in mean change (95% CI)	p-value	Cohen’s d effect size

Child psychological symptoms: Total	Baseline	32.0 (1.5)	31.7 (29.3)			
	Endline	27.6 (1.5)	25.9 (1.3)	1.5 (−2.5, 5.5)	0.46	0.21
Child psychological symptoms: Internalizing	Baseline	5.6 (0.3)	6.0 (0.3)			
	Endline	5.0 (0.3)	4.4 (0.3)	1.0 (0.1, 1.9)	0.03	0.56
Child psychological symptoms: Externalizing	Baseline	3.8 (0.4)	3.7 (0.3)			
	Endline	3.5 (0.4)	3.1 (0.3)	0.4 (−0.7, 1.5)	0.48	0.19
PHQ	Baseline	14.7 (1.1)	15.0 (2.1)			
	Endline	12.6 (1.1)	10.2 (2.1)	2.7 (0.1, 5.3)	0.04	0.59

Means, SEs, difference in mean change are based on coefficients and combination of coefficients from mixed effects model following multiple imputation. Cohen’s d effect size was calculated by dividing the predicted difference in mean change from the mixed effects model by the pooled baseline standard deviation. Model included fixed effects of arm, time and arm X time interaction, and random effects of pt_code, family ID, and EASE group.

## Data Availability

Data and materials will be shared upon request to the corresponding author.
