# Supplementary material for: Evaluation of the Early Adolescent Skills for Emotions (EASE) intervention in Lebanon: A randomized controlled trial
Source: Compr Psychiatry. Author manuscript; Available in PMC 2024 Nov 1. (PMC11214135; doi:10.1016/j.comppsych.2023.152424)
Supplement: Supplementary Materials [file NIHMS1998492-supplement-Supplementary_Materials.docx]

Supplementary File 1: Results sub-group analyses

## Child Psychological Symptoms (PSC) 4.1a Child Psychological Symptoms (PSC): by Gender

| **Table 4.1ai Predicted means, mean changes, and effect sizes for Child PSC outcome with imputed data among MALES** | | | | | | | | | | | | |
| --- | --- | --- | --- | --- | --- | --- | --- | --- | --- | --- | --- | --- |
|  |  | **EASE (N=43)** | | |  | **PYA (N=58)** | | |  | **Between group treatment effect** | | |
|  |  | *Mean*  *(95% CI)^a^* |  | *Mean change from baseline*  *(95% CI)*  *p* |  | *Mean*  *(95% CI)^a^* |  | *Mean change from baseline*  *(95% CI)*  *p* |  | *Mean difference in change*  *(95% CI)^b^*  *p* |  | *Cohen’s d^c^* |
| T0 |  | 28.9  (26.0, 31.8) |  | - |  | 26.1  (23.6, 28.6) |  | - |  | - |  | - |
| T1 |  | 22.3  (19.4, 25.2) |  | -6.6  (-9.3, -3.8)  **<.0001** |  | 21.0  (18.4, 23.6) |  | -5.1  (-7.5, -2.7)  **<.0001** |  | -1.5  (-5.2, 2.2)  .44 |  | 0.17 |
| ^a^Means, SDs, difference in mean change are based on coefficients and combination of coefficients from mixed effects model following multiple imputation.  ^b^Negative mean difference in change in this table means that the *decrease* in PSC score was larger in EASE than PYA  ^c^ Cohen’s d effect size was calculated by dividing the predicted difference in mean change from the mixed effects model by the pooled baseline SD (8.7).  ^*^Model included fixed effects of arm, time and arm X time interaction, and random effects of pt_code, family ID, and Ease group.  T0=Baseline, T1=Endline | | | | | | | | | | | | |

| **Table 4.1aii Predicted means, mean changes, and effect sizes for Child PSC outcome with imputed data among FEMALES** | | | | | | | | | | | | |
| --- | --- | --- | --- | --- | --- | --- | --- | --- | --- | --- | --- | --- |
|  |  | **EASE (N=37)** | | |  | **PYA (N=60)** | | |  | **Between group treatment effect** | | |
|  |  | *Mean*  *(95% CI)^a^* |  | *Mean change from baseline*  *(95% CI)*  *p* |  | *Mean*  *(95% CI)^a^* |  | *Mean change from baseline*  *(95% CI)*  *p* |  | *Mean difference in change*  *(95% CI)^b^*  *p* |  | *Cohen’s d^c^* |
| T0 |  | 25.8  (21.8, 29.8) |  | - |  | 28.2  (22.3, 34.0) |  | - |  | - |  | - |
| T1 |  | 21.8  (17.8, 26.0) |  | -3.9  (-6.9, -0.9)  **.01** |  | 24.3  (18.4, 30.3) |  | -3.8  (-6.3, -1.3)  **<.01** |  | -0.11  (-4.1, 3.8)  .96 |  | 0.01 |
| ^a^Means, SDs, difference in mean change are based on coefficients and combination of coefficients from mixed effects model following multiple imputation.  ^b^Negative mean difference in change in this table means that the *decrease* in PSC score was larger in EASE than PYA  ^c^ Cohen’s d effect size was calculated by dividing the predicted difference in mean change from the mixed effects model by the pooled baseline SD (7.5).  ^*^Model included fixed effects of arm, time and arm X time interaction, and random effects of pt_code, family ID, and Ease group.  T0=Baseline, T1=Endline | | | | | | | | | | | | |

### 4.1b Child Psychological Symptoms (PSC): by Age

| **Table 4.1bi Predicted means, mean changes, and effect sizes for Child PSC outcome with imputed data among 10-12 year olds** | | | | | | | | | | | | |
| --- | --- | --- | --- | --- | --- | --- | --- | --- | --- | --- | --- | --- |
|  |  | **EASE (N=56)** | | |  | **PYA (N=77)** | | |  | **Between group treatment effect** | | |
|  |  | *Mean*  *(95% CI)^a^* |  | *Mean change from baseline*  *(95% CI)*  *p* |  | *Mean*  *(95% CI)^a^* |  | *Mean change from baseline*  *(95% CI)*  *p* |  | *Mean difference in change*  *(95% CI)^b^*  *p* |  | *Cohen’s d^c^* |
| T0 |  | 26.1  (23.7, 29.6) |  | - |  | 26.0  (20.3, 31.6) |  | - |  | - |  | - |
| T1 |  | 20.3  (17.3, 23.3) |  | -6.3  (-8.7, -3.9)  **<.0001** |  | 21.8  (16.1, 27.4) |  | -4.2  (-6.3, -2.2)  **<.0001** |  | -2.1  (-5.3, 1.1)  .20 |  | 0.26 |
| ^a^Means, SDs, difference in mean change are based on coefficients and combination of coefficients from mixed effects model following multiple imputation.  ^b^Negative mean difference in change in this table means that the *decrease* in PSC score was larger in EASE than PYA  ^c^ Cohen’s d effect size was calculated by dividing the predicted difference in mean change from the mixed effects model by the pooled baseline SD (8.0).  ^*^Model included fixed effects of arm, time and arm X time interaction, and random effects of pt_code, family ID, and Ease group.  T0=Baseline, T1=Endline | | | | | | | | | | | | |

| **Table 4.1bii Predicted means, mean changes, and effect sizes for Child PSC outcome with imputed data among 13-14 year olds** | | | | | | | | | | | | |
| --- | --- | --- | --- | --- | --- | --- | --- | --- | --- | --- | --- | --- |
|  |  | **EASE (N=24)** | | |  | **PYA (N=41)** | | |  | **Between group treatment effect** | | |
|  |  | *Mean*  *(95% CI)^a^* |  | *Mean change from baseline*  *(95% CI)*  *p* |  | *Mean*  *(95% CI)^a^* |  | *Mean change from baseline*  *(95% CI)*  *p* |  | *Mean difference in change*  *(95% CI)^b^*  *p* |  | *Cohen’s d^c^* |
| T0 |  | 28.9  (24.7, 33.1) |  | - |  | 28.7  (25.5, 31.9) |  | - |  | - |  | - |
| T1 |  | 25.8  (21.5, 30.1) |  | -3.1  (-7.0, 0.82)  .12 |  | 23.9  (20.6, 27.2) |  | -4.8  (-7.9, -1.8)  **<.01** |  | 1.7  (-3.2, 6.7)  .49 |  | 0.20 |
| ^a^Means, SDs, difference in mean change are based on coefficients and combination of coefficients from mixed effects model following multiple imputation.  ^b^Positive mean difference in change in this table means that the *decrease* in PSC score was larger in PYA than EASE  ^c^ Cohen’s d effect size was calculated by dividing the predicted difference in mean change from the mixed effects model by the pooled baseline SD (8.3).  ^*^Model included fixed effects of arm, time and arm X time interaction, and random effects of pt_code, family ID, and Ease group.  T0=Baseline, T1=Endline | | | | | | | | | | | | |

### 4.1c Child Psychological Symptoms (PSC): by PSC Severity

| **Table 4.1ci Predicted means, mean changes, and effect sizes for Child PSC outcome with imputed data among lower 50% PSC Scores** | | | | | | | | | | | | |
| --- | --- | --- | --- | --- | --- | --- | --- | --- | --- | --- | --- | --- |
|  |  | **EASE (N=37)** | | |  | **PYA (N=59)** | | |  | **Between group treatment effect** | | |
|  |  | *Mean*  *(95% CI)^a^* |  | *Mean change from baseline*  *(95% CI)*  *p* |  | *Mean*  *(95% CI)^a^* |  | *Mean change from baseline*  *(95% CI)*  *p* |  | *Mean difference in change*  *(95% CI)^b^*  *p* |  | *Cohen’s d^c^* |
| T0 |  | 20.6  (18.2, 23.0) |  | - |  | 20.9  (18.9, 22.8) |  | - |  | - |  | - |
| T1 |  | 16.0  (13.5, 18.5) |  | -4.6  (-7.3, 1.9)  **<.01** |  | 18.8  (16.8, 20.8) |  | -2.1  (-4.2, 0.01)  .05 |  | -2.5  (-6.0, 0.94)  .15 |  | 0.61 |
| ^a^Means, SDs, difference in mean change are based on coefficients and combination of coefficients from mixed effects model following multiple imputation.  ^b^Negative mean difference in change in this table means that the *decrease* in PSC score was larger in EASE than PYA  ^c^ Cohen’s d effect size was calculated by dividing the predicted difference in mean change from the mixed effects model by the pooled baseline SD (4.1).  ^*^Model included fixed effects of arm, time and arm X time interaction, and random effects of pt_code, family ID, and Ease group.  T0=Baseline, T1=Endline | | | | | | | | | | | | |

| **Table 4.1cii Predicted means, mean changes, and effect sizes for Child PSC outcome with imputed data among upper 50% PSC Scores** | | | | | | | | | | | | |
| --- | --- | --- | --- | --- | --- | --- | --- | --- | --- | --- | --- | --- |
|  |  | **EASE (N=43)** | | |  | **PYA (N=59)** | | |  | **Between group treatment effect** | | |
|  |  | *Mean*  *(95% CI)^a^* |  | *Mean change from baseline*  *(95% CI)*  *p* |  | *Mean*  *(95% CI)^a^* |  | *Mean change from baseline*  *(95% CI)*  *p* |  | *Mean difference in change*  *(95% CI)^b^*  *p* |  | *Cohen’s d^c^* |
| T0 |  | 33.3  (30.5, 36.1) |  | - |  | 33.4  (30.9, 35.8) |  | - |  | - |  | - |
| T1 |  | 27.3  (24.5, 30.2) |  | -6.0  (-8.9, -3.0)  **<.0001** |  | 26.6  (24.1, 29.2) |  | -6.8  (-9.4, -4.1)  **<.0001** |  | 0.80  (-3.2, 4.8)  .70 |  | 0.13 |
| ^a^Means, SDs, difference in mean change are based on coefficients and combination of coefficients from mixed effects model following multiple imputation.  ^b^Positive mean difference in change in this table means that the *decrease* in PSC score was larger in PYA than EASE  ^c^ Cohen’s d effect size was calculated by dividing the predicted difference in mean change from the mixed effects model by the pooled baseline SD (6.0).  ^*^Model included fixed effects of arm, time and arm X time interaction, and random effects of pt_code, family ID, and Ease group.  T0=Baseline, T1=Endline | | | | | | | | | | | | |

### 4.1d Child Psychological Symptoms (PSC): by PSC Internalizing Severity

| **Table 4.1di Predicted means, mean changes, and effect sizes for Child PSC outcome with imputed data among lower 50% PSC Internalizing Scores** | | | | | | | | | | | | |
| --- | --- | --- | --- | --- | --- | --- | --- | --- | --- | --- | --- | --- |
|  |  | **EASE (N=43)** | | |  | **PYA (N=66)** | | |  | **Between group treatment effect** | | |
|  |  | *Mean*  *(95% CI)^a^* |  | *Mean change from baseline*  *(95% CI)*  *p* |  | *Mean*  *(95% CI)^a^* |  | *Mean change from baseline*  *(95% CI)*  *p* |  | *Mean difference in change*  *(95% CI)^b^*  *p* |  | *Cohen’s d^c^* |
| T0 |  | 24.6  (21.9, 27.3) |  | - |  | 23.5  (21.3, 25.8) |  | - |  | - |  | - |
| T1 |  | 20.9  (18.1, 23.7) |  | -3.7  (-6.4, -0.99)  **<.01** |  | 20.7  (18.4, 23.0) |  | -2.9  (-5.1, -0.76)  **<.01** |  | -0.80  (-4.3, 2.7)  .66 |  | 0.12 |
| ^a^Means, SDs, difference in mean change are based on coefficients and combination of coefficients from mixed effects model following multiple imputation.  ^b^Negative mean difference in change in this table means that the *decrease* in PSC score was larger in EASE than PYA  ^c^ Cohen’s d effect size was calculated by dividing the predicted difference in mean change from the mixed effects model by the pooled baseline SD (6.8).  ^*^Model included fixed effects of arm, time and arm X time interaction, and random effects of pt_code, family ID, and Ease group.  T0=Baseline, T1=Endline | | | | | | | | | | | | |

| **Table 4.1dii Predicted means, mean changes, and effect sizes for Child PSC outcome with imputed data among upper 50% PSC Internalizing Scores** | | | | | | | | | | | | |
| --- | --- | --- | --- | --- | --- | --- | --- | --- | --- | --- | --- | --- |
|  |  | **EASE (N=37)** | | |  | **PYA (N=52)** | | |  | **Between group treatment effect** | | |
|  |  | *Mean*  *(95% CI)^a^* |  | *Mean change from baseline*  *(95% CI)*  *p* |  | *Mean*  *(95% CI)^a^* |  | *Mean change from baseline*  *(95% CI)*  *p* |  | *Mean difference in change*  *(95% CI)^b^*  *p* |  | *Cohen’s d^c^* |
| T0 |  | 30.3  (26.9, 33.8) |  | - |  | 31.3  (26.8, 35.9) |  | - |  | - |  | - |
| T1 |  | 23.1  (19.6, 26.6) |  | -7.2  (-10.4, -4.1)  **<.0001** |  | 24.9  (20.3, 29.6) |  | -6.4  (-9.1, -3.6)  **<.0001** |  | -0.86  (-5.0, 3.3)  .69 |  | 0.11 |
| ^a^Means, SDs, difference in mean change are based on coefficients and combination of coefficients from mixed effects model following multiple imputation.  ^b^Negative mean difference in change in this table means that the *decrease* in PSC score was larger in EASE than PYA  ^c^ Cohen’s d effect size was calculated by dividing the predicted difference in mean change from the mixed effects model by the pooled baseline SD (8.0).  ^*^Model included fixed effects of arm, time and arm X time interaction, and random effects of pt_code, family ID, and Ease group.  T0=Baseline, T1=Endline | | | | | | | | | | | | |

### 4.1e Child Psychological Symptoms (PSC): by PSC Externalizing Severity

| **Table 4.1ei Predicted means, mean changes, and effect sizes for Child PSC outcome with imputed data among lower 50% PSC Externalizing Scores** | | | | | | | | | | | | |
| --- | --- | --- | --- | --- | --- | --- | --- | --- | --- | --- | --- | --- |
|  |  | **EASE (N=39)** | | |  | **PYA (N=56)** | | |  | **Between group treatment effect** | | |
|  |  | *Mean*  *(95% CI)^a^* |  | *Mean change from baseline*  *(95% CI)*  *p* |  | *Mean*  *(95% CI)^a^* |  | *Mean change from baseline*  *(95% CI)*  *p* |  | *Mean difference in change*  *(95% CI)^b^*  *p* |  | *Cohen’s d^c^* |
| T0 |  | 23.8  (20.8, 26.8) |  | - |  | 25.5  (20.8, 30.2) |  | - |  | - |  | - |
| T1 |  | 19.7  (16.6, 22.8) |  | -4.1  (-6.7, -1.5)  **<.01** |  | 22.2  (17.4, 26.9) |  | -3.3  (-5.6, -1.2)  **<.01** |  | -0.78  (-4.2, 2.7)  .66 |  | 0.12 |
| ^a^Means, SDs, difference in mean change are based on coefficients and combination of coefficients from mixed effects model following multiple imputation.  ^b^Negative mean difference in change in this table means that the *decrease* in PSC score was larger in EASE than PYA  ^c^ Cohen’s d effect size was calculated by dividing the predicted difference in mean change from the mixed effects model by the pooled baseline SD (6.6).  ^*^Model included fixed effects of arm, time and arm X time interaction, and random effects of pt_code, family ID, and Ease group.  T0=Baseline, T1=Endline | | | | | | | | | | | | |

| **Table 4.1eii Predicted means, mean changes, and effect sizes for Child PSC outcome with imputed data among upper 50% PSC Externalizing Scores** | | | | | | | | | | | | |
| --- | --- | --- | --- | --- | --- | --- | --- | --- | --- | --- | --- | --- |
|  |  | **EASE (N=41)** | | |  | **PYA (N=62)** | | |  | **Between group treatment effect** | | |
|  |  | *Mean*  *(95% CI)^a^* |  | *Mean change from baseline*  *(95% CI)*  *p* |  | *Mean*  *(95% CI)^a^* |  | *Mean change from baseline*  *(95% CI)*  *p* |  | *Mean difference in change*  *(95% CI)^b^*  *p* |  | *Cohen’s d^c^* |
| T0 |  | 30.5  (27.2, 33.7) |  | - |  | 28.6  (25.8, 31.3) |  | - |  | - |  | - |
| T1 |  | 24.0  (20.7, 27.3) |  | -6.5  (-9.6, -3.3)  **<.0001** |  | 23.2  (20.4, 26.0) |  | -5.4  (-8.0, -2.9)  **<.0001** |  | -1.1  (-5.1, 3.0)  .61 |  | 0.12 |
| ^a^Means, SDs, difference in mean change are based on coefficients and combination of coefficients from mixed effects model following multiple imputation.  ^b^Negative mean difference in change in this table means that the *decrease* in PSC score was larger in EASE than PYA  ^c^ Cohen’s d effect size was calculated by dividing the predicted difference in mean change from the mixed effects model by the pooled baseline SD (8.9).  ^*^Model included fixed effects of arm, time and arm X time interaction, and random effects of pt_code, family ID, and Ease group.  T0=Baseline, T1=Endline | | | | | | | | | | | | |

### 4.1f Child Psychological Symptoms (PSC): by PHQ Severity

| **Table 4.1fi Predicted means, mean changes, and effect sizes for Child PSC outcome with imputed data among lower 50% PHQ Scores** | | | | | | | | | | | | |
| --- | --- | --- | --- | --- | --- | --- | --- | --- | --- | --- | --- | --- |
|  |  | **EASE (N=40)** | | |  | **PYA (N=57)** | | |  | **Between group treatment effect** | | |
|  |  | *Mean*  *(95% CI)^a^* |  | *Mean change from baseline*  *(95% CI)*  *p* |  | *Mean*  *(95% CI)^a^* |  | *Mean change from baseline*  *(95% CI)*  *p* |  | *Mean difference in change*  *(95% CI)^b^*  *p* |  | *Cohen’s d^c^* |
| T0 |  | 22.9  (20.3, 25.4) |  | - |  | 22.3  (19.6, 25.0) |  | - |  | - |  | - |
| T1 |  | 16.5  (13.9, 19.1) |  | -6.3  (-9.0, -3.7)  **<.0001** |  | 19.4  (16.6, 22.1) |  | -2.9  (-5.2, -0.68)  **.01** |  | -3.4  (-7.0, 0.12)  .06 |  | 0.57 |
| ^a^Means, SDs, difference in mean change are based on coefficients and combination of coefficients from mixed effects model following multiple imputation.  ^b^Negative mean difference in change in this table means that the *decrease* in PSC score was larger in EASE than PYA  ^c^ Cohen’s d effect size was calculated by dividing the predicted difference in mean change from the mixed effects model by the pooled baseline SD (6.0).  ^*^Model included fixed effects of arm, time and arm X time interaction, and random effects of pt_code, family ID, and Ease group.  T0=Baseline, T1=Endline | | | | | | | | | | | | |

| **Table 4.1fii Predicted means, mean changes, and effect sizes for Child PSC outcome with imputed data among upper 50% PHQ Scores** | | | | | | | | | | | | |
| --- | --- | --- | --- | --- | --- | --- | --- | --- | --- | --- | --- | --- |
|  |  | **EASE (N=40)** | | |  | **PYA (N=61)** | | |  | **Between group treatment effect** | | |
|  |  | *Mean*  *(95% CI)^a^* |  | *Mean change from baseline*  *(95% CI)*  *p* |  | *Mean*  *(95% CI)^a^* |  | *Mean change from baseline*  *(95% CI)*  *p* |  | *Mean difference in change*  *(95% CI)^b^*  *p* |  | *Cohen’s d^c^* |
| T0 |  | 32.0  (29.0, 34.9) |  | - |  | 31.7  (29.3, 34.1) |  | - |  | - |  | - |
| T1 |  | 27.6  (24.7, 30.6) |  | -4.3  (-7.4, -1.2)  **<.01** |  | 25.9  (23.4, 28.4) |  | -5.8  (-8.4, -3.3)  **<.0001** |  | 1.5  (-2.5, 5.5)  .46 |  | 0.21 |
| ^a^Means, SDs, difference in mean change are based on coefficients and combination of coefficients from mixed effects model following multiple imputation.  ^b^Positive mean difference in change in this table means that the *decrease* in PSC score was larger in PYA than EASE  ^c^ Cohen’s d effect size was calculated by dividing the predicted difference in mean change from the mixed effects model by the pooled baseline SD (7.2).  ^*^Model included fixed effects of arm, time and arm X time interaction, and random effects of pt_code, family ID, and Ease group.  T0=Baseline, T1=Endline | | | | | | | | | | | | |

### 4.1g Child Psychological Symptoms (PSC): by Child Wellbeing Severity

| **Table 4.1gi Predicted means, mean changes, and effect sizes for Child PSC outcome with imputed data among lower 50% Child Wellbeing Scores** | | | | | | | | | | | | |
| --- | --- | --- | --- | --- | --- | --- | --- | --- | --- | --- | --- | --- |
|  |  | **EASE (N=36)** | | |  | **PYA (N=58)** | | |  | **Between group treatment effect** | | |
|  |  | *Mean*  *(95% CI)^a^* |  | *Mean change from baseline*  *(95% CI)*  *p* |  | *Mean*  *(95% CI)^a^* |  | *Mean change from baseline*  *(95% CI)*  *p* |  | *Mean difference in change*  *(95% CI)^b^*  *p* |  | *Cohen’s d^c^* |
| T0 |  | 30.6  (27.3, 33.9) |  | - |  | 28.9  (26.3, 31.5) |  | - |  | - |  | - |
| T1 |  | 26.5  (23.1, 29.8) |  | -4.1  (-7.3, -0.91)  **.01** |  | 24.9  (22.2, 27.5) |  | -4.0  (-6.5, -1.5)  **<.01** |  | -0.07  (-4.1, 4.0)  .97 |  | 0.01 |
| ^a^Means, SDs, difference in mean change are based on coefficients and combination of coefficients from mixed effects model following multiple imputation.  ^b^Negative mean difference in change in this table means that the *decrease* in PSC score was larger in EASE than PYA  ^c^ Cohen’s d effect size was calculated by dividing the predicted difference in mean change from the mixed effects model by the pooled baseline SD (8.5).  ^*^Model included fixed effects of arm, time and arm X time interaction, and random effects of pt_code, family ID, and Ease group.  T0=Baseline, T1=Endline | | | | | | | | | | | | |

| **Table 4.1gii Predicted means, mean changes, and effect sizes for Child PSC outcome with imputed data among upper 50% Child Wellbeing Scores** | | | | | | | | | | | | |
| --- | --- | --- | --- | --- | --- | --- | --- | --- | --- | --- | --- | --- |
|  |  | **EASE (N=44)** | | |  | **PYA (N=60)** | | |  | **Between group treatment effect** | | |
|  |  | *Mean*  *(95% CI)^a^* |  | *Mean change from baseline*  *(95% CI)*  *p* |  | *Mean*  *(95% CI)^a^* |  | *Mean change from baseline*  *(95% CI)*  *p* |  | *Mean difference in change*  *(95% CI)^b^*  *p* |  | *Cohen’s d^c^* |
| T0 |  | 24.4  (21.1, 27.7) |  | - |  | 25.3  (18.2, 32.3) |  | - |  | - |  | - |
| T1 |  | 18.1  (14.8, 21.4) |  | -6.3  (-9.0, -3.7)  **<.0001** |  | 20.4  (13.3, 27.6) |  | -4.8  (-7.2, -2.5)  **<.0001** |  | -1.5  (-5.1, 2.1)  .41 |  | 0.21 |
| ^a^Means, SDs, difference in mean change are based on coefficients and combination of coefficients from mixed effects model following multiple imputation.  ^b^Negative mean difference in change in this table means that the *decrease* in PSC score was larger in EASE than PYA  ^c^ Cohen’s d effect size was calculated by dividing the predicted difference in mean change from the mixed effects model by the pooled baseline SD (7.2).  ^*^Model included fixed effects of arm, time and arm X time interaction, and random effects of pt_code, family ID, and Ease group.  T0=Baseline, T1=Endline | | | | | | | | | | | | |

### 4.1h Child Psychological Symptoms (PSC): by Caregiver PSC Severity

| **Table 4.1hi Predicted means, mean changes, and effect sizes for Child PSC outcome with imputed data among lower 50% Caregiver PSC Scores** | | | | | | | | | | | | |
| --- | --- | --- | --- | --- | --- | --- | --- | --- | --- | --- | --- | --- |
|  |  | **EASE (N=36)** | | |  | **PYA (N=59)** | | |  | **Between group treatment effect** | | |
|  |  | *Mean*  *(95% CI)^a^* |  | *Mean change from baseline*  *(95% CI)*  *p* |  | *Mean*  *(95% CI)^a^* |  | *Mean change from baseline*  *(95% CI)*  *p* |  | *Mean difference in change*  *(95% CI)^b^*  *p* |  | *Cohen’s d^c^* |
| T0 |  | 23.5  (20.0, 27.0) |  | - |  | 24.5  (17.9, 31.1) |  | - |  | - |  | - |
| T1 |  | 18.1  (14.5, 21.6) |  | -5.4  (-8.4, -2.4)  **<.001** |  | 20.3  (13.6, 26.9) |  | -4.2  (-6.7, -1.8)  **<.01** |  | -1.2  (-5.2, 2.8)  .55 |  | 0.16 |
| ^a^Means, SDs, difference in mean change are based on coefficients and combination of coefficients from mixed effects model following multiple imputation.  ^b^Negative mean difference in change in this table means that the *decrease* in PSC score was larger in EASE than PYA  ^c^ Cohen’s d effect size was calculated by dividing the predicted difference in mean change from the mixed effects model by the pooled baseline SD (7.5).  ^*^Model included fixed effects of arm, time and arm X time interaction, and random effects of pt_code, family ID, and Ease group.  T0=Baseline, T1=Endline | | | | | | | | | | | | |

| **Table 4.1hii Predicted means, mean changes, and effect sizes for Child PSC outcome with imputed data among upper 50% Caregiver PSC Scores** | | | | | | | | | | | | |
| --- | --- | --- | --- | --- | --- | --- | --- | --- | --- | --- | --- | --- |
|  |  | **EASE (N=44)** | | |  | **PYA (N=59)** | | |  | **Between group treatment effect** | | |
|  |  | *Mean*  *(95% CI)^a^* |  | *Mean change from baseline*  *(95% CI)*  *p* |  | *Mean*  *(95% CI)^a^* |  | *Mean change from baseline*  *(95% CI)*  *p* |  | *Mean difference in change*  *(95% CI)^b^*  *p* |  | *Cohen’s d^c^* |
| T0 |  | 30.9  (27.9, 33.8) |  | - |  | 29.8  (27.1, 32.4) |  | - |  | - |  | - |
| T1 |  | 25.6  (22.6. 28.6) |  | -5.3  (-8.0, -2.5)  **<.0001** |  | 25.1  (22.4, 27.8) |  | -4.6  (-7.1, -2.2)  **<.0001** |  | -0.62  (-4.3, 3.0)  .74 |  | 0.08 |
| ^a^Means, SDs, difference in mean change are based on coefficients and combination of coefficients from mixed effects model following multiple imputation.  ^b^Negative mean difference in change in this table means that the *decrease* in PSC score was larger in EASE than PYA  ^c^ Cohen’s d effect size was calculated by dividing the predicted difference in mean change from the mixed effects model by the pooled baseline SD (7.7).  ^*^Model included fixed effects of arm, time and arm X time interaction, and random effects of pt_code, family ID, and Ease group.  T0=Baseline, T1=Endline | | | | | | | | | | | | |

### 4.1i Child Psychological Symptoms (PSC): by Caregiver K6 Severity

| **Table 4.1ii Predicted means, mean changes, and effect sizes for Child PSC outcome with imputed data among lower 50% Caregiver K6 Scores** | | | | | | | | | | | | |
| --- | --- | --- | --- | --- | --- | --- | --- | --- | --- | --- | --- | --- |
|  |  | **EASE (N=45)** | | |  | **PYA (N=48)** | | |  | **Between group treatment effect** | | |
|  |  | *Mean*  *(95% CI)^a^* |  | *Mean change from baseline*  *(95% CI)*  *p* |  | *Mean*  *(95% CI)^a^* |  | *Mean change from baseline*  *(95% CI)*  *p* |  | *Mean difference in change*  *(95% CI)^b^*  *p* |  | *Cohen’s d^c^* |
| T0 |  | 27.0  (23.8, 30.2) |  | - |  | 26.0  (21.3, 30.8) |  | - |  | - |  | - |
| T1 |  | 22.4  (19.2, 25.6) |  | -4.6  (7.2, -2.0)  **<.0001** |  | 19.9  (15.1, 24.8) |  | -6.1  (-8.7, -3.5)  **<.0001** |  | 1.5  (-2.2, 5.2)  .42 |  | 0.17 |
| ^a^Means, SDs, difference in mean change are based on coefficients and combination of coefficients from mixed effects model following multiple imputation.  ^b^Positive mean difference in change in this table means that the *decrease* in PSC score was larger in PYA than EASE  ^c^ Cohen’s d effect size was calculated by dividing the predicted difference in mean change from the mixed effects model by the pooled baseline SD (8.7).  ^*^Model included fixed effects of arm, time and arm X time interaction, and random effects of pt_code, family ID, and Ease group.  T0=Baseline, T1=Endline | | | | | | | | | | | | |

| **Table 4.1iii Predicted means, mean changes, and effect sizes for Child PSC outcome with imputed data among upper 50% Caregiver K6 Scores** | | | | | | | | | | | | |
| --- | --- | --- | --- | --- | --- | --- | --- | --- | --- | --- | --- | --- |
|  |  | **EASE (N=35)** | | |  | **PYA (N=70)** | | |  | **Between group treatment effect** | | |
|  |  | *Mean*  *(95% CI)^a^* |  | *Mean change from baseline*  *(95% CI)*  *p* |  | *Mean*  *(95% CI)^a^* |  | *Mean change from baseline*  *(95% CI)*  *p* |  | *Mean difference in change*  *(95% CI)^b^*  *p* |  | *Cohen’s d^c^* |
| T0 |  | 27.8  (24.5, 31.0) |  | - |  | 27.8  (25.0, 30.5) |  | - |  | - |  | - |
| T1 |  | 21.5  (18.1, 24.8) |  | -6.3  (-9.5, -3.1)  **<.0001** |  | 24.5  (21.7, 27.2) |  | -3.3  (-5.5, -1.0)  **<.01** |  | -3.1  (-6.9, 0.93)  .14 |  | 0.41 |
| ^a^Means, SDs, difference in mean change are based on coefficients and combination of coefficients from mixed effects model following multiple imputation.  ^b^Negative mean difference in change in this table means that the *decrease* in PSC score was larger in EASE than PYA  ^c^ Cohen’s d effect size was calculated by dividing the predicted difference in mean change from the mixed effects model by the pooled baseline SD (7.6).  ^*^Model included fixed effects of arm, time and arm X time interaction, and random effects of pt_code, family ID, and Ease group.  T0=Baseline, T1=Endline | | | | | | | | | | | | |

### 4.1j Child Psychological Symptoms (PSC): Treatment Completers

| **Table 4.1ji Predicted means, mean changes, and effect sizes for Child PSC outcome with imputed data among treatment completers** | | | | | | | | | | | | |
| --- | --- | --- | --- | --- | --- | --- | --- | --- | --- | --- | --- | --- |
|  |  | **EASE (N=56)** | | |  | **PYA (N=117)** | | |  | **Between group treatment effect** | | |
|  |  | *Mean*  *(95% CI)^a^* |  | *Mean change from baseline*  *(95% CI)*  *p* |  | *Mean*  *(95% CI)^a^* |  | *Mean change from baseline*  *(95% CI)*  *p* |  | *Mean difference in change*  *(95% CI)^b^*  *p* |  | *Cohen’s d^c^* |
| T0 |  | 28.6  (26.0, 31.1) |  | - |  | 27.1  (25.2, 28.9) |  | - |  | - |  | - |
| T1 |  | 23.0  (20.4, 25.5) |  | -5.6  (-8.0, -3.2)  **<.0001** |  | 22.6  (20.7, 24.5) |  | -4.4  (-6.2, -2.7)  **<.0001** |  | -1.2  (-4.1, 1.8)  .44 |  | 0.15 |
| ^a^Means, SDs, difference in mean change are based on coefficients and combination of coefficients from mixed effects model following multiple imputation.  ^b^Negative mean difference in change in this table means that the *decrease* in PSC score was larger in EASE than PYA  ^c^ Cohen’s d effect size was calculated by dividing the predicted difference in mean change from the mixed effects model by the pooled baseline SD (7.8).  ^*^Model included fixed effects of arm, time and arm X time interaction, and random effects of pt_code, family ID, and Ease group.  T0=Baseline, T1=Endline | | | | | | | | | | | | |

## Child Psychological Symptoms (PSC): Internalizing Subscale

### 4.3a Child Psychological Symptoms (PSC): Internalizing Subscale: by Gender

| **Table 4.3ai Predicted means, mean changes, and effect sizes for Child PSC Internalizing outcome with imputed data among MALES** | | | | | | | | | | | | |
| --- | --- | --- | --- | --- | --- | --- | --- | --- | --- | --- | --- | --- |
|  |  | **EASE (N=43)** | | |  | **PYA (N=58)** | | |  | **Between group treatment effect** | | |
|  |  | *Mean*  *(95% CI)^a^* |  | *Mean change from baseline*  *(95% CI)*  *p* |  | *Mean*  *(95% CI)^a^* |  | *Mean change from baseline*  *(95% CI)*  *p* |  | *Mean difference in change*  *(95% CI)^b^*  *p* |  | *Cohen’s d^c^* |
| T0 |  | 5.7  (5.0, 6.3) |  | - |  | 4.9  (4.4, 5.5) |  | - |  | - |  | - |
| T1 |  | 3.5  (2.9, 4.2) |  | -2.1  (-2.8, -1.4)  **<.0001** |  | 3.4  (2.9, 4.0) |  | -1.5  (-2.1, -0.88)  **<.0001** |  | -0.61  (-1.5, 0.33)  .20 |  | 0.34 |
| ^a^Means, SDs, difference in mean change are based on coefficients and combination of coefficients from mixed effects model following multiple imputation.  ^b^Negative mean difference in change in this table means that the *decrease* in PSC score was larger in EASE than PYA  ^c^ Cohen’s d effect size was calculated by dividing the predicted difference in mean change from the mixed effects model by the pooled baseline SD (1.8).  ^*^Model included fixed effects of arm, time and arm X time interaction, and random effects of pt_code, family ID, and Ease group.  T0=Baseline, T1=Endline | | | | | | | | | | | | |

| **Table 4.3aii Predicted means, mean changes, and effect sizes for Child PSC Internalizing outcome with imputed data among FEMALES** | | | | | | | | | | | | |
| --- | --- | --- | --- | --- | --- | --- | --- | --- | --- | --- | --- | --- |
|  |  | **EASE (N=37)** | | |  | **PYA (N=60)** | | |  | **Between group treatment effect** | | |
|  |  | *Mean*  *(95% CI)^a^* |  | *Mean change from baseline*  *(95% CI)*  *p* |  | *Mean*  *(95% CI)^a^* |  | *Mean change from baseline*  *(95% CI)*  *p* |  | *Mean difference in change*  *(95% CI)^b^*  *p* |  | *Cohen’s d^c^* |
| T0 |  | 5.0  (4.1, 5.9) |  | - |  | 5.8  (4.3, 7.2) |  | - |  | - |  | - |
| T1 |  | 4.1  (3.2, 5.0) |  | -0.88  (-1.7, -0.11)  **.03** |  | 4.2  (2.7, 5.6) |  | -1.6  (-2.2, -0.98)  **<.0001** |  | 0.72  (-0.27, 1.7)  .15 |  | 0.34 |
| ^a^Means, SDs, difference in mean change are based on coefficients and combination of coefficients from mixed effects model following multiple imputation.  ^b^Positive mean difference in change in this table means that the *decrease* in PSC score was larger in PYA than EASE  ^c^ Cohen’s d effect size was calculated by dividing the predicted difference in mean change from the mixed effects model by the pooled baseline SD (2.1).  ^*^Model included fixed effects of arm, time and arm X time interaction, and random effects of pt_code, family ID, and Ease group.  T0=Baseline, T1=Endline | | | | | | | | | | | | |

### 4.3b Child Psychological Symptoms (PSC): Internalizing Subscale: by Age

| **Table 4.3bi Predicted means, mean changes, and effect sizes for Child PSC Internalizing outcome with imputed data among 10-12 year olds** | | | | | | | | | | | | |
| --- | --- | --- | --- | --- | --- | --- | --- | --- | --- | --- | --- | --- |
|  |  | **EASE (N=56)** | | |  | **PYA (N=77)** | | |  | **Between group treatment effect** | | |
|  |  | *Mean*  *(95% CI)^a^* |  | *Mean change from baseline*  *(95% CI)*  *p* |  | *Mean*  *(95% CI)^a^* |  | *Mean change from baseline*  *(95% CI)*  *p* |  | *Mean difference in change*  *(95% CI)^b^*  *p* |  | *Cohen’s d^c^* |
| T0 |  | 5.2  (4.7, 5.8) |  | - |  | 5.1  (4.6, 5.6) |  | - |  | - |  | - |
| T1 |  | 3.4  (2.9, 4.0) |  | -1.8  (-2.4, -1.2)  **<.0001** |  | 3.7  (3.2, 4.2) |  | -1.4  (-2.0, -0.88)  **<.0001** |  | -0.38  (-1.2, 0.45)  .37 |  | 0.2 |
| ^a^Means, SDs, difference in mean change are based on coefficients and combination of coefficients from mixed effects model following multiple imputation.  ^b^Negative mean difference in change in this table means that the *decrease* in PSC score was larger in EASE than PYA  ^c^ Cohen’s d effect size was calculated by dividing the predicted difference in mean change from the mixed effects model by the pooled baseline SD (1.9).  ^*^Model included fixed effects of arm, time and arm X time interaction, and random effects of pt_code, family ID, and Ease group.  T0=Baseline, T1=Endline | | | | | | | | | | | | |

| **Table 4.3bii Predicted means, mean changes, and effect sizes for Child PSC Internalizing outcome with imputed data among 13-14 year olds** | | | | | | | | | | | | |
| --- | --- | --- | --- | --- | --- | --- | --- | --- | --- | --- | --- | --- |
|  |  | **EASE (N=24)** | | |  | **PYA (N=41)** | | |  | **Between group treatment effect** | | |
|  |  | *Mean*  *(95% CI)^a^* |  | *Mean change from baseline*  *(95% CI)*  *p* |  | *Mean*  *(95% CI)^a^* |  | *Mean change from baseline*  *(95% CI)*  *p* |  | *Mean difference in change*  *(95% CI)^b^*  *p* |  | *Cohen’s d^c^* |
| T0 |  | 5.6  (4.5, 6.7) |  | - |  | 5.7  (4.0, 7.4) |  | - |  | - |  | - |
| T1 |  | 4.6  (3.5, 5.8) |  | -0.95  (-1.9, 0.05)  .06 |  | 3.9  (2.2, 5.6) |  | -1.8  (-2.6, -1.0)  **<.0001** |  | 0.86  (-0.39, 2.1)  .18 |  | 0.43 |
| ^a^Means, SDs, difference in mean change are based on coefficients and combination of coefficients from mixed effects model following multiple imputation.  ^b^Positive mean difference in change in this table means that the *decrease* in PSC score was larger in PYA than EASE  ^c^ Cohen’s d effect size was calculated by dividing the predicted difference in mean change from the mixed effects model by the pooled baseline SD (2.0).  ^*^Model included fixed effects of arm, time and arm X time interaction, and random effects of pt_code, family ID, and Ease group.  T0=Baseline, T1=Endline | | | | | | | | | | | | |

### 4.3c Child Psychological Symptoms (PSC): Internalizing Subscale: by PSC Severity

| **Table 4.3ci Predicted means, mean changes, and effect sizes for Child PSC Internalizing outcome with imputed data among lower 50% PSC Scores** | | | | | | | | | | | | |
| --- | --- | --- | --- | --- | --- | --- | --- | --- | --- | --- | --- | --- |
|  |  | **EASE (N=37)** | | |  | **PYA (N=59)** | | |  | **Between group treatment effect** | | |
|  |  | *Mean*  *(95% CI)^a^* |  | *Mean change from baseline*  *(95% CI)*  *p* |  | *Mean*  *(95% CI)^a^* |  | *Mean change from baseline*  *(95% CI)*  *p* |  | *Mean difference in change*  *(95% CI)^b^*  *p* |  | *Cohen’s d^c^* |
| T0 |  | 4.5  (3.8, 5.1) |  | - |  | 4.6  (4.1, 5.1) |  | - |  | - |  | - |
| T1 |  | 2.7  (2.0, 3.3) |  | -1.8  (-2.6, -1.0)  **<.0001** |  | 3.2  (2.6, 3.7) |  | -1.4  (-2.0, -0.77)  **<.0001** |  | -0.40  (-1.4, 0.59)  .43 |  | 0.22 |
| ^a^Means, SDs, difference in mean change are based on coefficients and combination of coefficients from mixed effects model following multiple imputation.  ^b^Negative mean difference in change in this table means that the *decrease* in PSC score was larger in EASE than PYA  ^c^ Cohen’s d effect size was calculated by dividing the predicted difference in mean change from the mixed effects model by the pooled baseline SD (1.8).  ^*^Model included fixed effects of arm, time and arm X time interaction, and random effects of pt_code, family ID, and Ease group.  T0=Baseline, T1=Endline | | | | | | | | | | | | |

| **Table 4.3cii Predicted means, mean changes, and effect sizes for Child PSC Internalizing outcome with imputed data among upper 50% PSC Scores** | | | | | | | | | | | | |
| --- | --- | --- | --- | --- | --- | --- | --- | --- | --- | --- | --- | --- |
|  |  | **EASE (N=43)** | | |  | **PYA (N=59)** | | |  | **Between group treatment effect** | | |
|  |  | *Mean*  *(95% CI)^a^* |  | *Mean change from baseline*  *(95% CI)*  *p* |  | *Mean*  *(95% CI)^a^* |  | *Mean change from baseline*  *(95% CI)*  *p* |  | *Mean difference in change*  *(95% CI)^b^*  *p* |  | *Cohen’s d^c^* |
| T0 |  | 6.1  (5.5, 6.7) |  | - |  | 6.1  (5.6, 6.7) |  | - |  | - |  | - |
| T1 |  | 4.8  (4.2, 5.4) |  | -1.3  (-2.0, -0.58)  **<.0001** |  | 4.4  (3.8, 5.0) |  | -1.7  (-2.3, -1.1)  **<.0001** |  | 0.39  (-0.56, 1.3)  .42 |  | 0.21 |
| ^a^Means, SDs, difference in mean change are based on coefficients and combination of coefficients from mixed effects model following multiple imputation.  ^b^Positive mean difference in change in this table means that the *decrease* in PSC score was larger in PYA than EASE  ^c^ Cohen’s d effect size was calculated by dividing the predicted difference in mean change from the mixed effects model by the pooled baseline SD (1.8).  ^*^Model included fixed effects of arm, time and arm X time interaction, and random effects of pt_code, family ID, and Ease group.  T0=Baseline, T1=Endline | | | | | | | | | | | | |

### 4.3d Child Psychological Symptoms (PSC): Internalizing Subscale: by PSC Internalizing Severity

| **Table 4.3di Predicted means, mean changes, and effect sizes for Child PSC Internalizing outcome with imputed data among lower 50% PSC Internalizing Scores** | | | | | | | | | | | | |
| --- | --- | --- | --- | --- | --- | --- | --- | --- | --- | --- | --- | --- |
|  |  | **EASE (N=43)** | | |  | **PYA (N=66)** | | |  | **Between group treatment effect** | | |
|  |  | *Mean*  *(95% CI)^a^* |  | *Mean change from baseline*  *(95% CI)*  *p* |  | *Mean*  *(95% CI)^a^* |  | *Mean change from baseline*  *(95% CI)*  *p* |  | *Mean difference in change*  *(95% CI)^b^*  *p* |  | *Cohen’s d^c^* |
| T0 |  | 3.9  (3.3, 4.4) |  | - |  | 3.9  (3.4, 4.4) |  | - |  | - |  | - |
| T1 |  | 3.1  (2.5, 3.7) |  | -0.74  (-1.4, -0.08)  **.03** |  | 3.4  (2.9, 3.9) |  | -0.50  (-1.0, 0.04)  .07 |  | -0.24  (-1.1, 0.61)  .58 |  | 0.22 |
| ^a^Means, SDs, difference in mean change are based on coefficients and combination of coefficients from mixed effects model following multiple imputation.  ^b^Negative mean difference in change in this table means that the *decrease* in PSC score was larger in EASE than PYA  ^c^ Cohen’s d effect size was calculated by dividing the predicted difference in mean change from the mixed effects model by the pooled baseline SD (1.1).  ^*^Model included fixed effects of arm, time and arm X time interaction, and random effects of pt_code, family ID, and Ease group.  T0=Baseline, T1=Endline | | | | | | | | | | | | |

| **Table 4.3dii Predicted means, mean changes, and effect sizes for Child PSC Internalizing outcome with imputed data among upper 50% PSC Internalizing Scores** | | | | | | | | | | | | |
| --- | --- | --- | --- | --- | --- | --- | --- | --- | --- | --- | --- | --- |
|  |  | **EASE (N=37)** | | |  | **PYA (N=52)** | | |  | **Between group treatment effect** | | |
|  |  | *Mean*  *(95% CI)^a^* |  | *Mean change from baseline*  *(95% CI)*  *p* |  | *Mean*  *(95% CI)^a^* |  | *Mean change from baseline*  *(95% CI)*  *p* |  | *Mean difference in change*  *(95% CI)^b^*  *p* |  | *Cohen’s d^c^* |
| T0 |  | 7.0  (6.3, 7.7) |  | - |  | 7.2  (6.3, 8.0) |  | - |  | - |  | - |
| T1 |  | 4.6  (3.8, 5.3) |  | -2.5  (-3.2, -1.7)  **<.0001** |  | 4.3  (3.4, 5.1) |  | -2.9  (-3.6, -2.2)  **<.0001** |  | 0.42  (-0.61, 1.5)  .42 |  | 0.38 |
| ^a^Means, SDs, difference in mean change are based on coefficients and combination of coefficients from mixed effects model following multiple imputation.  ^b^Positive mean difference in change in this table means that the *decrease* in PSC score was larger in PYA than EASE  ^c^ Cohen’s d effect size was calculated by dividing the predicted difference in mean change from the mixed effects model by the pooled baseline SD (2.1).  ^*^Model included fixed effects of arm, time and arm X time interaction, and random effects of pt_code, family ID, and Ease group.  T0=Baseline, T1=Endline | | | | | | | | | | | | |

### 4.3e Child Psychological Symptoms (PSC): Internalizing Subscale: by PSC Externalizing Severity

| **Table 4.3ei Predicted means, mean changes, and effect sizes for Child PSC Internalizing outcome with imputed data among lower 50% PSC Externalizing Scores** | | | | | | | | | | | | |
| --- | --- | --- | --- | --- | --- | --- | --- | --- | --- | --- | --- | --- |
|  |  | **EASE (N=39)** | | |  | **PYA (N=56)** | | |  | **Between group treatment effect** | | |
|  |  | *Mean*  *(95% CI)^a^* |  | *Mean change from baseline*  *(95% CI)*  *p* |  | *Mean*  *(95% CI)^a^* |  | *Mean change from baseline*  *(95% CI)*  *p* |  | *Mean difference in change*  *(95% CI)^b^*  *p* |  | *Cohen’s d^c^* |
| T0 |  | 5.1  (4.4, 5.9) |  | - |  | 5.6  (4.4, 6.8) |  | - |  | - |  | - |
| T1 |  | 3.6  (2.8, 4.3) |  | -1.6  (-2.4, -0.81)  **<.0001** |  | 3.9  (2.8, 5.1) |  | -1.7  (-2.3, -1.0)  **<.0001** |  | 0.08  (-0.92, 1.1)  .87 |  | 0.04 |
| ^a^Means, SDs, difference in mean change are based on coefficients and combination of coefficients from mixed effects model following multiple imputation.  ^b^Negative mean difference in change in this table means that the *decrease* in PSC score was larger in EASE than PYA  ^c^ Cohen’s d effect size was calculated by dividing the predicted difference in mean change from the mixed effects model by the pooled baseline SD (1.9).  ^*^Model included fixed effects of arm, time and arm X time interaction, and random effects of pt_code, family ID, and Ease group.  T0=Baseline, T1=Endline | | | | | | | | | | | | |

| **Table 4.3eii Predicted means, mean changes, and effect sizes for Child PSC outcome with imputed data among upper 50% PSC Externalizing Scores** | | | | | | | | | | | | |
| --- | --- | --- | --- | --- | --- | --- | --- | --- | --- | --- | --- | --- |
|  |  | **EASE (N=41)** | | |  | **PYA (N=62)** | | |  | **Between group treatment effect** | | |
|  |  | *Mean*  *(95% CI)^a^* |  | *Mean change from baseline*  *(95% CI)*  *p* |  | *Mean*  *(95% CI)^a^* |  | *Mean change from baseline*  *(95% CI)*  *p* |  | *Mean difference in change*  *(95% CI)^b^*  *p* |  | *Cohen’s d^c^* |
| T0 |  | 5.5  (4.8, 6.1) |  | - |  | 5.1  (4.5, 5.7) |  | - |  | - |  | - |
| T1 |  | 4.0  (3.3, 4.6) |  | -1.5  (-2.2, -0.77)  **<.0001** |  | 3.6  (3.1, 4.2) |  | -1.5  (-2.1, -0.84)  **<.0001** |  | -0.05  (-1.0, 0.90)  .92 |  | 0.03 |
| ^a^Means, SDs, difference in mean change are based on coefficients and combination of coefficients from mixed effects model following multiple imputation.  ^b^Negative mean difference in change in this table means that the *decrease* in PSC score was larger in EASE than PYA  ^c^ Cohen’s d effect size was calculated by dividing the predicted difference in mean change from the mixed effects model by the pooled baseline SD (2.0).  ^*^Model included fixed effects of arm, time and arm X time interaction, and random effects of pt_code, family ID, and Ease group.  T0=Baseline, T1=Endline | | | | | | | | | | | | |

### 4.3f Child Psychological Symptoms (PSC): Internalizing Subscale: by PHQ Severity

| **Table 4.3fi Predicted means, mean changes, and effect sizes for Child PSC Internalizing outcome with imputed data among lower 50% PHQ Scores** | | | | | | | | | | | | |
| --- | --- | --- | --- | --- | --- | --- | --- | --- | --- | --- | --- | --- |
|  |  | **EASE (N=40)** | | |  | **PYA (N=57)** | | |  | **Between group treatment effect** | | |
|  |  | *Mean*  *(95% CI)^a^* |  | *Mean change from baseline*  *(95% CI)*  *p* |  | *Mean*  *(95% CI)^a^* |  | *Mean change from baseline*  *(95% CI)*  *p* |  | *Mean difference in change*  *(95% CI)^b^*  *p* |  | *Cohen’s d^c^* |
| T0 |  | 5.1  (4.5, 5.7) |  | - |  | 4.7  (4.2, 5.2) |  | - |  | - |  | - |
| T1 |  | 2.6  (2.0, 3.2) |  | -2.5  (-3.3, -1.8)  **<.0001** |  | 3.1  (2.6, 3.7) |  | -1.5  (-2.2, -0.89)  **<.0001** |  | -0.98  (-2.0, 0.02)  .05 |  | 0.49 |
| ^a^Means, SDs, difference in mean change are based on coefficients and combination of coefficients from mixed effects model following multiple imputation.  ^b^Negative mean difference in change in this table means that the *decrease* in PSC score was larger in EASE than PYA  ^c^ Cohen’s d effect size was calculated by dividing the predicted difference in mean change from the mixed effects model by the pooled baseline SD (2.0).  ^*^Model included fixed effects of arm, time and arm X time interaction, and random effects of pt_code, family ID, and Ease group.  T0=Baseline, T1=Endline | | | | | | | | | | | | |

| **Table 4.3fii Predicted means, mean changes, and effect sizes for Child PSC Internalizing outcome with imputed data among upper 50% PHQ Scores** | | | | | | | | | | | | |
| --- | --- | --- | --- | --- | --- | --- | --- | --- | --- | --- | --- | --- |
|  |  | **EASE (N=40)** | | |  | **PYA (N=61)** | | |  | **Between group treatment effect** | | |
|  |  | *Mean*  *(95% CI)^a^* |  | *Mean change from baseline*  *(95% CI)*  *p* |  | *Mean*  *(95% CI)^a^* |  | *Mean change from baseline*  *(95% CI)*  *p* |  | *Mean difference in change*  *(95% CI)^b^*  *p* |  | *Cohen’s d^c^* |
| T0 |  | 5.6  (4.9, 6.2) |  | - |  | 6.0  (5.3, 6.6) |  | - |  | - |  | - |
| T1 |  | 5.0  (4.4, 5.7) |  | -0.56  (-1.3, 0.16)  .13 |  | 4.4  (3.8, 5.1) |  | -1.6  (-2.2, -0.97)  **<.0001** |  | 1.0  (0.08, 1.9)  **.03** |  | 0.56 |
| ^a^Means, SDs, difference in mean change are based on coefficients and combination of coefficients from mixed effects model following multiple imputation.  ^b^Positive mean difference in change in this table means that the *decrease* in PSC score was larger in PYA than EASE  ^c^ Cohen’s d effect size was calculated by dividing the predicted difference in mean change from the mixed effects model by the pooled baseline SD (1.8).  ^*^Model included fixed effects of arm, time and arm X time interaction, and random effects of pt_code, family ID, and Ease group.  T0=Baseline, T1=Endline | | | | | | | | | | | | |

### 4.3g Child Psychological Symptoms (PSC): Internalizing Subscale: by Child Wellbeing Severity

| **Table 4.3gi Predicted means, mean changes, and effect sizes for Child PSC Internalizing outcome with imputed data among lower 50% Child Wellbeing Scores** | | | | | | | | | | | | |
| --- | --- | --- | --- | --- | --- | --- | --- | --- | --- | --- | --- | --- |
|  |  | **EASE (N=36)** | | |  | **PYA (N=58)** | | |  | **Between group treatment effect** | | |
|  |  | *Mean*  *(95% CI)^a^* |  | *Mean change from baseline*  *(95% CI)*  *p* |  | *Mean*  *(95% CI)^a^* |  | *Mean change from baseline*  *(95% CI)*  *p* |  | *Mean difference in change*  *(95% CI)^b^*  *p* |  | *Cohen’s d^c^* |
| T0 |  | 5.7  (4.9, 6.4) |  | - |  | 5.6  (5.0, 6.2) |  | - |  | - |  | - |
| T1 |  | 4.6  (3.9, 5.4) |  | -1.1  (-1.9, -0.23)  **.01** |  | 4.0  (3.5, 4.6) |  | -1.6  (-2.2, -0.92)  **<.0001** |  | 0.51  (-0.52, 1.5)  .33 |  | 0.27 |
| ^a^Means, SDs, difference in mean change are based on coefficients and combination of coefficients from mixed effects model following multiple imputation.  ^b^Positive mean difference in change in this table means that the *decrease* in PSC score was larger in PYA than EASE  ^c^ Cohen’s d effect size was calculated by dividing the predicted difference in mean change from the mixed effects model by the pooled baseline SD (1.9).  ^*^Model included fixed effects of arm, time and arm X time interaction, and random effects of pt_code, family ID, and Ease group.  T0=Baseline, T1=Endline | | | | | | | | | | | | |

| **Table 4.3gii Predicted means, mean changes, and effect sizes for Child PSC Internalizing outcome with imputed data among upper 50% Child Wellbeing Scores** | | | | | | | | | | | | |
| --- | --- | --- | --- | --- | --- | --- | --- | --- | --- | --- | --- | --- |
|  |  | **EASE (N=44)** | | |  | **PYA (N=60)** | | |  | **Between group treatment effect** | | |
|  |  | *Mean*  *(95% CI)^a^* |  | *Mean change from baseline*  *(95% CI)*  *p* |  | *Mean*  *(95% CI)^a^* |  | *Mean change from baseline*  *(95% CI)*  *p* |  | *Mean difference in change*  *(95% CI)^b^*  *p* |  | *Cohen’s d^c^* |
| T0 |  | 5.0  (4.3, 5.7) |  | - |  | 5.1  (3.9, 6.2) |  | - |  | - |  | - |
| T1 |  | 3.1  (2.4, 3.8) |  | -1.9  (-2.6, -1.2)  **<.0001** |  | 3.5  (2.3, 4.7) |  | -1.5  (-2.2, -0.93)  **<.0001** |  | -0.40  (-1.3, 0.52)  .40 |  | 0.20 |
| ^a^Means, SDs, difference in mean change are based on coefficients and combination of coefficients from mixed effects model following multiple imputation.  ^b^Negative mean difference in change in this table means that the *decrease* in PSC score was larger in EASE than PYA  ^c^ Cohen’s d effect size was calculated by dividing the predicted difference in mean change from the mixed effects model by the pooled baseline SD (2.0).  ^*^Model included fixed effects of arm, time and arm X time interaction, and random effects of pt_code, family ID, and Ease group.  T0=Baseline, T1=Endline | | | | | | | | | | | | |

### 4.3h Child Psychological Symptoms (PSC): Internalizing Subscale: by Caregiver PSC Severity

| **Table 4.3hi Predicted means, mean changes, and effect sizes for Child PSC Internalizing outcome with imputed data among lower 50% Caregiver PSC Scores** | | | | | | | | | | | | |
| --- | --- | --- | --- | --- | --- | --- | --- | --- | --- | --- | --- | --- |
|  |  | **EASE (N=36)** | | |  | **PYA (N=59)** | | |  | **Between group treatment effect** | | |
|  |  | *Mean*  *(95% CI)^a^* |  | *Mean change from baseline*  *(95% CI)*  *p* |  | *Mean*  *(95% CI)^a^* |  | *Mean change from baseline*  *(95% CI)*  *p* |  | *Mean difference in change*  *(95% CI)^b^*  *p* |  | *Cohen’s d^c^* |
| T0 |  | 5.1  (4.4, 5.7) |  | - |  | 5.0  (4.3, 5.7) |  | - |  | - |  | - |
| T1 |  | 3.5  (2.8, 4.2) |  | -1.6  (-2.4, -0.74)  **<.0001** |  | 3.3  (2.6, 4.0) |  | -1.7  (-2.3, -1.1)  **<.0001** |  | 0.14  (-0.90, 1.2)  .79 |  | 0.07 |
| ^a^Means, SDs, difference in mean change are based on coefficients and combination of coefficients from mixed effects model following multiple imputation.  ^b^Positive mean difference in change in this table means that the *decrease* in PSC score was larger in PYA than EASE  ^c^ Cohen’s d effect size was calculated by dividing the predicted difference in mean change from the mixed effects model by the pooled baseline SD (2.0).  ^*^Model included fixed effects of arm, time and arm X time interaction, and random effects of pt_code, family ID, and Ease group.  T0=Baseline, T1=Endline | | | | | | | | | | | | |

| **Table 4.3hii Predicted means, mean changes, and effect sizes for Child PSC Internalizing outcome with imputed data among upper 50% Caregiver PSC Scores** | | | | | | | | | | | | |
| --- | --- | --- | --- | --- | --- | --- | --- | --- | --- | --- | --- | --- |
|  |  | **EASE (N=44)** | | |  | **PYA (N=59)** | | |  | **Between group treatment effect** | | |
|  |  | *Mean*  *(95% CI)^a^* |  | *Mean change from baseline*  *(95% CI)*  *p* |  | *Mean*  *(95% CI)^a^* |  | *Mean change from baseline*  *(95% CI)*  *p* |  | *Mean difference in change*  *(95% CI)^b^*  *p* |  | *Cohen’s d^c^* |
| T0 |  | 5.6  (5.0, 6.3) |  | - |  | 5.7  (5.1, 6.2) |  | - |  | - |  | - |
| T1 |  | 4.1  (3.4, 4.7) |  | -1.5  (-2.2, -0.84)  **<.0001** |  | 4.2  (3.7, 4.8) |  | -1.4  (-2.0, -0.81)  **<.0001** |  | -0.12  (-1.0, 0.79)  .80 |  | 0.06 |
| ^a^Means, SDs, difference in mean change are based on coefficients and combination of coefficients from mixed effects model following multiple imputation.  ^b^Negative mean difference in change in this table means that the *decrease* in PSC score was larger in EASE than PYA  ^c^ Cohen’s d effect size was calculated by dividing the predicted difference in mean change from the mixed effects model by the pooled baseline SD (1.9).  ^*^Model included fixed effects of arm, time and arm X time interaction, and random effects of pt_code, family ID, and Ease group.  T0=Baseline, T1=Endline | | | | | | | | | | | | |

### 4.3i Child Psychological Symptoms (PSC): Internalizing Subscale: by Caregiver K6 Severity

| **Table 4.3ii Predicted means, mean changes, and effect sizes for Child PSC Internalizing outcome with imputed data among lower 50% Caregiver K6 Scores** | | | | | | | | | | | | |
| --- | --- | --- | --- | --- | --- | --- | --- | --- | --- | --- | --- | --- |
|  |  | **EASE (N=45)** | | |  | **PYA (N=48)** | | |  | **Between group treatment effect** | | |
|  |  | *Mean*  *(95% CI)^a^* |  | *Mean change from baseline*  *(95% CI)*  *p* |  | *Mean*  *(95% CI)^a^* |  | *Mean change from baseline*  *(95% CI)*  *p* |  | *Mean difference in change*  *(95% CI)^b^*  *p* |  | *Cohen’s d^c^* |
| T0 |  | 5.3  (4.6, 6.0) |  | - |  | 5.2  (4.2, 6.2) |  | - |  | - |  | - |
| T1 |  | 4.1  (3.3, 4.8) |  | -1.3  (-2.0, -0.48)  **<.01** |  | 3.4  (2.3, 4.4) |  | -1.8  (-2.6, -1.1)  **<.0001** |  | 0.63  (-0.40, 1.7)  .23 |  | 0.30 |
| ^a^Means, SDs, difference in mean change are based on coefficients and combination of coefficients from mixed effects model following multiple imputation.  ^b^Positive mean difference in change in this table means that the *decrease* in PSC score was larger in PYA than EASE  ^c^ Cohen’s d effect size was calculated by dividing the predicted difference in mean change from the mixed effects model by the pooled baseline SD (2.1).  ^*^Model included fixed effects of arm, time and arm X time interaction, and random effects of pt_code, family ID, and Ease group.  T0=Baseline, T1=Endline | | | | | | | | | | | | |

| **Table 4.3iii Predicted means, mean changes, and effect sizes for Child PSC Internalizing outcome with imputed data among upper 50% Caregiver K6 Scores** | | | | | | | | | | | | |
| --- | --- | --- | --- | --- | --- | --- | --- | --- | --- | --- | --- | --- |
|  |  | **EASE (N=35)** | | |  | **PYA (N=70)** | | |  | **Between group treatment effect** | | |
|  |  | *Mean*  *(95% CI)^a^* |  | *Mean change from baseline*  *(95% CI)*  *p* |  | *Mean*  *(95% CI)^a^* |  | *Mean change from baseline*  *(95% CI)*  *p* |  | *Mean difference in change*  *(95% CI)^b^*  *p* |  | *Cohen’s d^c^* |
| T0 |  | 5.4  (4.7, 6.1) |  | - |  | 5.4  (4.9, 5.9) |  | - |  | - |  | - |
| T1 |  | 3.5  (2.7, 4.2) |  | -2.0  (-2.8, -1.2)  **<.0001** |  | 4.0  (3.5, 4.6) |  | -1.4  (-1.9, -0.81)  **<.0001** |  | -0.61  (-1.6, 0.35)  .21 |  | 0.32 |
| ^a^Means, SDs, difference in mean change are based on coefficients and combination of coefficients from mixed effects model following multiple imputation.  ^b^Negative mean difference in change in this table means that the *decrease* in PSC score was larger in EASE than PYA  ^c^ Cohen’s d effect size was calculated by dividing the predicted difference in mean change from the mixed effects model by the pooled baseline SD (1.9).  ^*^Model included fixed effects of arm, time and arm X time interaction, and random effects of pt_code, family ID, and Ease group.  T0=Baseline, T1=Endline | | | | | | | | | | | | |

### 4.3j Child Psychological Symptoms (PSC): Internalizing Subscale: by Treatment Completer Status

| **Table 4.3ji Predicted means, mean changes, and effect sizes for Child PSC Internalizing outcome with imputed data among treatment completers** | | | | | | | | | | | | |
| --- | --- | --- | --- | --- | --- | --- | --- | --- | --- | --- | --- | --- |
|  |  | **EASE (N=56)** | | |  | **PYA (N=117)** | | |  | **Between group treatment effect** | | |
|  |  | *Mean*  *(95% CI)^a^* |  | *Mean change from baseline*  *(95% CI)*  *p* |  | *Mean*  *(95% CI)^a^* |  | *Mean change from baseline*  *(95% CI)*  *p* |  | *Mean difference in change*  *(95% CI)^b^*  *p* |  | *Cohen’s d^c^* |
| T0 |  | 5.5  (4.9, 6.1) |  | - |  | 5.3  (4.9, 5.7) |  | - |  | - |  | - |
| T1 |  | 3.9  (3.3, 4.4) |  | -1.6  (-2.2, -1.0)  **<.0001** |  | 3.8  (3.4, 4.2) |  | -1.6  (-2.0, -1.1)  **<.0001** |  | -0.07  (-0.83, 0.69)  .87 |  | 0.04 |
| ^a^Means, SDs, difference in mean change are based on coefficients and combination of coefficients from mixed effects model following multiple imputation.  ^b^Negative mean difference in change in this table means that the *decrease* in PSC score was larger in EASE than PYA  ^c^ Cohen’s d effect size was calculated by dividing the predicted difference in mean change from the mixed effects model by the pooled baseline SD (1.9).  ^*^Model included fixed effects of arm, time and arm X time interaction, and random effects of pt_code, family ID, and Ease group.  T0=Baseline, T1=Endline | | | | | | | | | | | | |

## 4.4 Child

## Psychological Symptoms (PSC): Externalizing Subscale

### 4.4a Child Psychological Symptoms (PSC): Externalizing Subscale: by Gender

| **Table 4.4ai Predicted means, mean changes, and effect sizes for Child PSC Externalizing outcome with imputed data among MALES** | | | | | | | | | | | | |
| --- | --- | --- | --- | --- | --- | --- | --- | --- | --- | --- | --- | --- |
|  |  | **EASE (N=43)** | | |  | **PYA (N=58)** | | |  | **Between group treatment effect** | | |
|  |  | *Mean*  *(95% CI)^a^* |  | *Mean change from baseline*  *(95% CI)*  *p* |  | *Mean*  *(95% CI)^a^* |  | *Mean change from baseline*  *(95% CI)*  *p* |  | *Mean difference in change*  *(95% CI)^b^*  *p* |  | *Cohen’s d^c^* |
| T0 |  | 4.2  (3.5, 4.8) |  | - |  | 4.0  (3.4, 4.6) |  | - |  | - |  | - |
| T1 |  | 3.4  (2.8, 4.1) |  | -0.77  (-1.5, -0.01)  **.04** |  | 3.2  (2.6, 3.8) |  | -0.77  (-1.4, -0.11)  **.02** |  | 0.01  (-1.0, 1.0)  .99 |  | 0.01 |
| ^a^Means, SDs, difference in mean change are based on coefficients and combination of coefficients from mixed effects model following multiple imputation.  ^b^Positive mean difference in change in this table means that the *decrease* in PSC score was larger in PYA than EASE  ^c^ Cohen’s d effect size was calculated by dividing the predicted difference in mean change from the mixed effects model by the pooled baseline SD (1.9).  ^*^Model included fixed effects of arm, time and arm X time interaction, and random effects of pt_code, family ID, and Ease group.  T0=Baseline, T1=Endline | | | | | | | | | | | | |

| **Table 4.4aii Predicted means, mean changes, and effect sizes for Child PSC Externalizing outcome with imputed data among FEMALES** | | | | | | | | | | | | |
| --- | --- | --- | --- | --- | --- | --- | --- | --- | --- | --- | --- | --- |
|  |  | **EASE (N=37)** | | |  | **PYA (N=60)** | | |  | **Between group treatment effect** | | |
|  |  | *Mean*  *(95% CI)^a^* |  | *Mean change from baseline*  *(95% CI)*  *p* |  | *Mean*  *(95% CI)^a^* |  | *Mean change from baseline*  *(95% CI)*  *p* |  | *Mean difference in change*  *(95% CI)^b^*  *p* |  | *Cohen’s d^c^* |
| T0 |  | 2.9  (2.2, 3.6) |  | - |  | 3.4  (2.8, 3.9) |  | - |  | - |  | - |
| T1 |  | 2.8  (2.1, 3.5) |  | -0.11  (-0.88, 0.66)  .78 |  | 2.7  (2.1, 3.2) |  | -0.70  (-1.3, -0.10)  **.02** |  | 0.59  (-0.39, 1.6)  .24 |  | 0.31 |
| ^a^Means, SDs, difference in mean change are based on coefficients and combination of coefficients from mixed effects model following multiple imputation.  ^b^Positive mean difference in change in this table means that the *decrease* in PSC score was larger in PYA than EASE  ^c^ Cohen’s d effect size was calculated by dividing the predicted difference in mean change from the mixed effects model by the pooled baseline SD (1.9).  ^*^Model included fixed effects of arm, time and arm X time interaction, and random effects of pt_code, family ID, and Ease group.  T0=Baseline, T1=Endline | | | | | | | | | | | | |

### 4.4b Child Psychological Symptoms (PSC): Externalizing Subscale: by Age

| **Table 4.4bi Predicted means, mean changes, and effect sizes for Child PSC Externalizing outcome with imputed data among 10-12 year olds** | | | | | | | | | | | | |
| --- | --- | --- | --- | --- | --- | --- | --- | --- | --- | --- | --- | --- |
|  |  | **EASE (N=56)** | | |  | **PYA (N=77)** | | |  | **Between group treatment effect** | | |
|  |  | *Mean*  *(95% CI)^a^* |  | *Mean change from baseline*  *(95% CI)*  *p* |  | *Mean*  *(95% CI)^a^* |  | *Mean change from baseline*  *(95% CI)*  *p* |  | *Mean difference in change*  *(95% CI)^b^*  *p* |  | *Cohen’s d^c^* |
| T0 |  | 3.7  (3.1, 4.3) |  | - |  | 3.5  (2.9, 4.1) |  | - |  | - |  | - |
| T1 |  | 3.0  (2.4, 3.6) |  | -0.70  (-1.4, -0.03)  **.04** |  | 2.9  (2.2, 3.5) |  | -0.61  (-1.2, -0.02)  **.04** |  | -0.09  (-0.98, 0.80)  .84 |  | 0.05 |
| ^a^Means, SDs, difference in mean change are based on coefficients and combination of coefficients from mixed effects model following multiple imputation.  ^b^Negative mean difference in change in this table means that the *decrease* in PSC score was larger in EASE than PYA  ^c^ Cohen’s d effect size was calculated by dividing the predicted difference in mean change from the mixed effects model by the pooled baseline SD (1.9).  ^*^Model included fixed effects of arm, time and arm X time interaction, and random effects of pt_code, family ID, and Ease group.  T0=Baseline, T1=Endline | | | | | | | | | | | | |

| **Table 4.4bii Predicted means, mean changes, and effect sizes for Child PSC Externalizing outcome with imputed data among 13-14 year olds** | | | | | | | | | | | | |
| --- | --- | --- | --- | --- | --- | --- | --- | --- | --- | --- | --- | --- |
|  |  | **EASE (N=24)** | | |  | **PYA (N=41)** | | |  | **Between group treatment effect** | | |
|  |  | *Mean*  *(95% CI)^a^* |  | *Mean change from baseline*  *(95% CI)*  *p* |  | *Mean*  *(95% CI)^a^* |  | *Mean change from baseline*  *(95% CI)*  *p* |  | *Mean difference in change*  *(95% CI)^b^*  *p* |  | *Cohen’s d^c^* |
| T0 |  | 3.3  (2.4, 4.2) |  | - |  | 4.0  (3.3, 4.7) |  | - |  | - |  | - |
| T1 |  | 3.4  (2.5, 4.3) |  | 0.1  (-0.81, 1.0)  .83 |  | 3.0  (2.3, 3.8) |  | -0.97  (-1.7, -0.26)  **<.01** |  | 1.1  (-0.09, 2.2)  .07 |  | 0.5 |
| ^a^Means, SDs, difference in mean change are based on coefficients and combination of coefficients from mixed effects model following multiple imputation.  ^b^Positive mean difference in change in this table means that the *decrease* in PSC score was larger in PYA than EASE  ^c^ Cohen’s d effect size was calculated by dividing the predicted difference in mean change from the mixed effects model by the pooled baseline SD (2.2).  ^*^Model included fixed effects of arm, time and arm X time interaction, and random effects of pt_code, family ID, and Ease group.  T0=Baseline, T1=Endline | | | | | | | | | | | | |

### 4.4c Child Psychological Symptoms (PSC): Externalizing Subscale: by PSC Severity

| **Table 4.4ci Predicted means, mean changes, and effect sizes for Child PSC Externalizing outcome with imputed data among lower 50% PSC Scores** | | | | | | | | | | | | |
| --- | --- | --- | --- | --- | --- | --- | --- | --- | --- | --- | --- | --- |
|  |  | **EASE (N=37)** | | |  | **PYA (N=59)** | | |  | **Between group treatment effect** | | |
|  |  | *Mean*  *(95% CI)^a^* |  | *Mean change from baseline*  *(95% CI)*  *p* |  | *Mean*  *(95% CI)^a^* |  | *Mean change from baseline*  *(95% CI)*  *p* |  | *Mean difference in change*  *(95% CI)^b^*  *p* |  | *Cohen’s d^c^* |
| T0 |  | 2.9  (2.3, 3.5) |  | - |  | 3.5  (3.0, 4.0) |  | - |  | - |  | - |
| T1 |  | 2.5  (1.9, 3.2) |  | -0.38  (-1.1, -.33)  **.29** |  | 2.8  (2.3, 3.3) |  | -0.70  (-1.3, -0.14)  **.01** |  | 0.32  (-0.58, 1.2)  .48 |  | 0.19 |
| ^a^Means, SDs, difference in mean change are based on coefficients and combination of coefficients from mixed effects model following multiple imputation.  ^b^Positive mean difference in change in this table means that the *decrease* in PSC score was larger in PYA than EASE  ^c^ Cohen’s d effect size was calculated by dividing the predicted difference in mean change from the mixed effects model by the pooled baseline SD (1.7).  ^*^Model included fixed effects of arm, time and arm X time interaction, and random effects of pt_code, family ID, and Ease group.  T0=Baseline, T1=Endline | | | | | | | | | | | | |

| **Table 4.4cii Predicted means, mean changes, and effect sizes for Child PSC Externalizing outcome with imputed data among upper 50% PSC Scores** | | | | | | | | | | | | |
| --- | --- | --- | --- | --- | --- | --- | --- | --- | --- | --- | --- | --- |
|  |  | **EASE (N=43)** | | |  | **PYA (N=59)** | | |  | **Between group treatment effect** | | |
|  |  | *Mean*  *(95% CI)^a^* |  | *Mean change from baseline*  *(95% CI)*  *p* |  | *Mean*  *(95% CI)^a^* |  | *Mean change from baseline*  *(95% CI)*  *p* |  | *Mean difference in change*  *(95% CI)^b^*  *p* |  | *Cohen’s d^c^* |
| T0 |  | 4.2  (3.5, 4.9) |  | - |  | 3.9  (3.3, 4.5) |  | - |  | - |  | - |
| T1 |  | 3.6  (2.9, 4.4) |  | -0.53  (-1.3, 0.28)  .20 |  | 3.1  (2.5, 3.8) |  | -0.76  (-1.5, -0.06)  **.03** |  | 0.23  (-0.85, 1.3)  .67 |  | 0.10 |
| ^a^Means, SDs, difference in mean change are based on coefficients and combination of coefficients from mixed effects model following multiple imputation.  ^b^Positive mean difference in change in this table means that the *decrease* in PSC score was larger in EASE than PYA  ^c^ Cohen’s d effect size was calculated by dividing the predicted difference in mean change from the mixed effects model by the pooled baseline SD (2.2).  ^*^Model included fixed effects of arm, time and arm X time interaction, and random effects of pt_code, family ID, and Ease group.  T0=Baseline, T1=Endline | | | | | | | | | | | | |

### 4.4d Child Psychological Symptoms (PSC): Externalizing Subscale: by PSC Internalizing Severity

| **Table 4.4di Predicted means, mean changes, and effect sizes for Child PSC Externalizing outcome with imputed data among lower 50% PSC Internalizing Scores** | | | | | | | | | | | | |
| --- | --- | --- | --- | --- | --- | --- | --- | --- | --- | --- | --- | --- |
|  |  | **EASE (N=37)** | | |  | **PYA (N=52)** | | |  | **Between group treatment effect** | | |
|  |  | *Mean*  *(95% CI)^a^* |  | *Mean change from baseline*  *(95% CI)*  *p* |  | *Mean*  *(95% CI)^a^* |  | *Mean change from baseline*  *(95% CI)*  *p* |  | *Mean difference in change*  *(95% CI)^b^*  *p* |  | *Cohen’s d^c^* |
| T0 |  | 3.7  (3.1, 4.3) |  | - |  | 4.0  (3.4, 4.6) |  | - |  | - |  | - |
| T1 |  | 3.5  (2.8, 4.1) |  | -0.22  (-0.94, -.49)  .54 |  | 3.1  (2.5, 3.7) |  | -0.88  (-1.5, -0.30)  **<.01** |  | 0.65  (-0.27, 1.6)  .16 |  | 0.36 |
| ^a^Means, SDs, difference in mean change are based on coefficients and combination of coefficients from mixed effects model following multiple imputation.  ^b^Positive mean difference in change in this table means that the *decrease* in PSC score was larger in PYA than EASE  ^c^ Cohen’s d effect size was calculated by dividing the predicted difference in mean change from the mixed effects model by the pooled baseline SD (1.8).  ^*^Model included fixed effects of arm, time and arm X time interaction, and random effects of pt_code, family ID, and Ease group.  T0=Baseline, T1=Endline | | | | | | | | | | | | |

| **Table 4.4dii Predicted means, mean changes, and effect sizes for Child PSC Externalizing outcome with imputed data among upper 50% PSC Internalizing Scores** | | | | | | | | | | | | |
| --- | --- | --- | --- | --- | --- | --- | --- | --- | --- | --- | --- | --- |
|  |  | **EASE (N=43)** | | |  | **PYA (N=66)** | | |  | **Between group treatment effect** | | |
|  |  | *Mean*  *(95% CI)^a^* |  | *Mean change from baseline*  *(95% CI)*  *p* |  | *Mean*  *(95% CI)^a^* |  | *Mean change from baseline*  *(95% CI)*  *p* |  | *Mean difference in change*  *(95% CI)^b^*  *p* |  | *Cohen’s d^c^* |
| T0 |  | 3.5  (2.7, 4.2) |  | - |  | 3.3  (2.7, 4.0) |  | - |  | - |  | - |
| T1 |  | 2.7  (2.0, 3.4) |  | -0.74  (-1.6, 0.09)  .08 |  | 2.8  (2.1, 3.4) |  | -0.56  (-1.3, 0.17)  .13 |  | -0.18  (-1.3, 0.92)  .74 |  | 0.09 |
| ^a^Means, SDs, difference in mean change are based on coefficients and combination of coefficients from mixed effects model following multiple imputation.  ^b^Negative mean difference in change in this table means that the *decrease* in PSC score was larger in EASE than PYA  ^c^ Cohen’s d effect size was calculated by dividing the predicted difference in mean change from the mixed effects model by the pooled baseline SD (2.1).  ^*^Model included fixed effects of arm, time and arm X time interaction, and random effects of pt_code, family ID, and Ease group.  T0=Baseline, T1=Endline | | | | | | | | | | | | |

### 4.4e Child Psychological Symptoms (PSC): Externalizing Subscale: by PSC Externalizing Severity

| **Table 4.4ei Predicted means, mean changes, and effect sizes for Child PSC Externalizing outcome with imputed data among lower 50% PSC Externalizing Scores** | | | | | | | | | | | | |
| --- | --- | --- | --- | --- | --- | --- | --- | --- | --- | --- | --- | --- |
|  |  | **EASE (N=39)** | | |  | **PYA (N=56)** | | |  | **Between group treatment effect** | | |
|  |  | *Mean*  *(95% CI)^a^* |  | *Mean change from baseline*  *(95% CI)*  *p* |  | *Mean*  *(95% CI)^a^* |  | *Mean change from baseline*  *(95% CI)*  *p* |  | *Mean difference in change*  *(95% CI)^b^*  *p* |  | *Cohen’s d^c^* |
| T0 |  | 1.9  (1.4, 2.5) |  | - |  | 2.1  (1.6, 2.6) |  | - |  | - |  | - |
| T1 |  | 2.3  (1.7, 2.9) |  | 0.38  (-0.36, 1.1)  .31 |  | 2.5  (2.0, 3.0) |  | 0.40  (-0.24, 1.0)  .22 |  | -0.02  (-0.99, 0.95)  .97 |  | 0.02 |
| ^a^Means, SDs, difference in mean change are based on coefficients and combination of coefficients from mixed effects model following multiple imputation.  ^b^Negative mean difference in change in this table means that the *increase* in PSC score was smaller in EASE than PYA  ^c^ Cohen’s d effect size was calculated by dividing the predicted difference in mean change from the mixed effects model by the pooled baseline SD (0.99).  ^*^Model included fixed effects of arm, time and arm X time interaction, and random effects of pt_code, family ID, and Ease group.  T0=Baseline, T1=Endline | | | | | | | | | | | | |

| **Table 4.4eii Predicted means, mean changes, and effect sizes for Child PSC Externalizing outcome with imputed data among upper 50% PSC Externalizing Scores** | | | | | | | | | | | | |
| --- | --- | --- | --- | --- | --- | --- | --- | --- | --- | --- | --- | --- |
|  |  | **EASE (N=41)** | | |  | **PYA (N=62)** | | |  | **Between group treatment effect** | | |
|  |  | *Mean*  *(95% CI)^a^* |  | *Mean change from baseline*  *(95% CI)*  *p* |  | *Mean*  *(95% CI)^a^* |  | *Mean change from baseline*  *(95% CI)*  *p* |  | *Mean difference in change*  *(95% CI)^b^*  *p* |  | *Cohen’s d^c^* |
| T0 |  | 5.1  (4.5, 5.8) |  | - |  | 5.1  (4.6, 5.7) |  | - |  | - |  | - |
| T1 |  | 3.9  (3.2, 4.6) |  | -1.3  (-2.0, -0.53)  **<.01** |  | 3.4  (2.8, 4.0) |  | -1.8  (-2.4, -1.1)  **<.0001** |  | 0.50  (-0.46, 1.5)  .31 |  | 0.36 |
| ^a^Means, SDs, difference in mean change are based on coefficients and combination of coefficients from mixed effects model following multiple imputation.  ^b^Positive mean difference in change in this table means that the *decrease* in PSC score was larger in PYA than EASE  ^c^ Cohen’s d effect size was calculated by dividing the predicted difference in mean change from the mixed effects model by the pooled baseline SD (1.4).  ^*^Model included fixed effects of arm, time and arm X time interaction, and random effects of pt_code, family ID, and Ease group.  T0=Baseline, T1=Endline | | | | | | | | | | | | |

### 4.4f Child Psychological Symptoms (PSC): Externalizing Subscale: by PHQ Severity

| **Table 4.4fi Predicted means, mean changes, and effect sizes for Child PSC Externalizing outcome with imputed data among lower 50% PHQ Scores** | | | | | | | | | | | | |
| --- | --- | --- | --- | --- | --- | --- | --- | --- | --- | --- | --- | --- |
|  |  | **EASE (N=40)** | | |  | **PYA (N=57)** | | |  | **Between group treatment effect** | | |
|  |  | *Mean*  *(95% CI)^a^* |  | *Mean change from baseline*  *(95% CI)*  *p* |  | *Mean*  *(95% CI)^a^* |  | *Mean change from baseline*  *(95% CI)*  *p* |  | *Mean difference in change*  *(95% CI)^b^*  *p* |  | *Cohen’s d^c^* |
| T0 |  | 3.4  (2.8, 4.1) |  | - |  | 3.6  (2.8, 4.5) |  | - |  | - |  | - |
| T1 |  | 2.7  (2.1, 3.4) |  | -0.70  (-1.4, -0.01)  **.04** |  | 2.8  (1.9, 3.6) |  | -0.86  (-1.5, -0.27)  **<.01** |  | 0.17  (-0.74, 1.1)  .72 |  | 0.09 |
| ^a^Means, SDs, difference in mean change are based on coefficients and combination of coefficients from mixed effects model following multiple imputation.  ^b^Positive mean difference in change in this table means that the *decrease* in PSC score was larger in PYA than EASE  ^c^ Cohen’s d effect size was calculated by dividing the predicted difference in mean change from the mixed effects model by the pooled baseline SD (1.9).  ^*^Model included fixed effects of arm, time and arm X time interaction, and random effects of pt_code, family ID, and Ease group.  T0=Baseline, T1=Endline | | | | | | | | | | | | |

| **Table 4.4fii Predicted means, mean changes, and effect sizes for Child PSC Externalizing outcome with imputed data among upper 50% PHQ Scores** | | | | | | | | | | | | |
| --- | --- | --- | --- | --- | --- | --- | --- | --- | --- | --- | --- | --- |
|  |  | **EASE (N=40)** | | |  | **PYA (N=61)** | | |  | **Between group treatment effect** | | |
|  |  | *Mean*  *(95% CI)^a^* |  | *Mean change from baseline*  *(95% CI)*  *p* |  | *Mean*  *(95% CI)^a^* |  | *Mean change from baseline*  *(95% CI)*  *p* |  | *Mean difference in change*  *(95% CI)^b^*  *p* |  | *Cohen’s d^c^* |
| T0 |  | 3.8  (3.0, 4.5) |  | - |  | 3.7  (3.1, 4.3) |  | - |  | - |  | - |
| T1 |  | 3.5  (2.8, 4.3) |  | -0.23  (-1.1, 0.60)  .59 |  | 3.1  (2.5, 3.7) |  | -0.61  (-1.3, 0.07)  .08 |  | 0.39  (-0.69, 1.5)  .48 |  | 0.19 |
| ^a^Means, SDs, difference in mean change are based on coefficients and combination of coefficients from mixed effects model following multiple imputation.  ^b^Positive mean difference in change in this table means that the *decrease* in PSC score was larger in PYA than EASE  ^c^ Cohen’s d effect size was calculated by dividing the predicted difference in mean change from the mixed effects model by the pooled baseline SD (2.1).  ^*^Model included fixed effects of arm, time and arm X time interaction, and random effects of pt_code, family ID, and Ease group.  T0=Baseline, T1=Endline | | | | | | | | | | | | |

### 4.4g Child Psychological Symptoms (PSC): Externalizing Subscale: by Child Wellbeing Severity

| **Table 4.4gi Predicted means, mean changes, and effect sizes for Child PSC Externalizing outcome with imputed data among lower 50% Child Wellbeing Scores** | | | | | | | | | | | | |
| --- | --- | --- | --- | --- | --- | --- | --- | --- | --- | --- | --- | --- |
|  |  | **EASE (N=36)** | | |  | **PYA (N=58)** | | |  | **Between group treatment effect** | | |
|  |  | *Mean*  *(95% CI)^a^* |  | *Mean change from baseline*  *(95% CI)*  *p* |  | *Mean*  *(95% CI)^a^* |  | *Mean change from baseline*  *(95% CI)*  *p* |  | *Mean difference in change*  *(95% CI)^b^*  *p* |  | *Cohen’s d^c^* |
| T0 |  | 3.9  (3.1, 4.6) |  | - |  | 4.0  (3.4, 4.6) |  | - |  | - |  | - |
| T1 |  | 3.6  (2.8, 4.4) |  | -0.25  (-1.1, 0.62)  .57 |  | 3.4  (2.8, 4.0) |  | -0.57  (-1.3, 0.12)  .10 |  | 0.32  (-0.79, 1.4)  .57 |  | 0.15 |
| ^a^Means, SDs, difference in mean change are based on coefficients and combination of coefficients from mixed effects model following multiple imputation.  ^b^Positive mean difference in change in this table means that the *decrease* in PSC score was larger in PYA than EASE  ^c^ Cohen’s d effect size was calculated by dividing the predicted difference in mean change from the mixed effects model by the pooled baseline SD (2.1).  ^*^Model included fixed effects of arm, time and arm X time interaction, and random effects of pt_code, family ID, and Ease group.  T0=Baseline, T1=Endline | | | | | | | | | | | | |

| **Table 4.4gii Predicted means, mean changes, and effect sizes for Child PSC Externalizing outcome with imputed data among upper 50% Child Wellbeing Scores** | | | | | | | | | | | | |
| --- | --- | --- | --- | --- | --- | --- | --- | --- | --- | --- | --- | --- |
|  |  | **EASE (N=44)** | | |  | **PYA (N=60)** | | |  | **Between group treatment effect** | | |
|  |  | *Mean*  *(95% CI)^a^* |  | *Mean change from baseline*  *(95% CI)*  *p* |  | *Mean*  *(95% CI)^a^* |  | *Mean change from baseline*  *(95% CI)*  *p* |  | *Mean difference in change*  *(95% CI)^b^*  *p* |  | *Cohen’s d^c^* |
| T0 |  | 3.4  (2.8, 4.0) |  | - |  | 3.4  (2.9, 3.9) |  | - |  | - |  | - |
| T1 |  | 2.7  (2.1, 3.3) |  | -0.63  (-1.3, 0.05)  .07 |  | 2.5  (2.0, 3.0) |  | -0.89  (-1.5, -0.30)  **<.01** |  | 0.26  (-0.63, 1.2)  .57 |  | 0.14 |
| ^a^Means, SDs, difference in mean change are based on coefficients and combination of coefficients from mixed effects model following multiple imputation.  ^b^Positive mean difference in change in this table means that the *decrease* in PSC score was larger in PYA than EASE  ^c^ Cohen’s d effect size was calculated by dividing the predicted difference in mean change from the mixed effects model by the pooled baseline SD (1.8).  ^*^Model included fixed effects of arm, time and arm X time interaction, and random effects of pt_code, family ID, and Ease group.  T0=Baseline, T1=Endline | | | | | | | | | | | | |

### 4.4h Child Psychological Symptoms (PSC): Externalizing Subscale: by Caregiver PSC Severity

| **Table 4.4hi Predicted means, mean changes, and effect sizes for Child PSC Externalizing outcome with imputed data among lower 50% Caregiver PSC Scores** | | | | | | | | | | | | |
| --- | --- | --- | --- | --- | --- | --- | --- | --- | --- | --- | --- | --- |
|  |  | **EASE (N=36)** | | |  | **PYA (N=59)** | | |  | **Between group treatment effect** | | |
|  |  | *Mean*  *(95% CI)^a^* |  | *Mean change from baseline*  *(95% CI)*  *p* |  | *Mean*  *(95% CI)^a^* |  | *Mean change from baseline*  *(95% CI)*  *p* |  | *Mean difference in change*  *(95% CI)^b^*  *p* |  | *Cohen’s d^c^* |
| T0 |  | 2.7  (2.1, 3.4) |  | - |  | 3.7  (3.2, 4.2) |  | - |  | - |  | - |
| T1 |  | 2.3  (1.6, 3.0) |  | -0.42  (-1.2, 0.36)  .29 |  | 2.9  (2.3, 3.4) |  | -0.86  (-1.5, -0.24)  **<.01** |  | 0.44  (-0.55, 1.4)  .39 |  | 0.24 |
| ^a^Means, SDs, difference in mean change are based on coefficients and combination of coefficients from mixed effects model following multiple imputation.  ^b^Positive mean difference in change in this table means that the *decrease* in PSC score was larger in PYA than EASE  ^c^ Cohen’s d effect size was calculated by dividing the predicted difference in mean change from the mixed effects model by the pooled baseline SD (1.8).  ^*^Model included fixed effects of arm, time and arm X time interaction, and random effects of pt_code, family ID, and Ease group.  T0=Baseline, T1=Endline | | | | | | | | | | | | |

| **Table 4.4hii Predicted means, mean changes, and effect sizes for Child PSC Externalizing outcome with imputed data among upper 50% Caregiver PSC Scores** | | | | | | | | | | | | |
| --- | --- | --- | --- | --- | --- | --- | --- | --- | --- | --- | --- | --- |
|  |  | **EASE (N=44)** | | |  | **PYA (N=59)** | | |  | **Between group treatment effect** | | |
|  |  | *Mean*  *(95% CI)^a^* |  | *Mean change from baseline*  *(95% CI)*  *p* |  | *Mean*  *(95% CI)^a^* |  | *Mean change from baseline*  *(95% CI)*  *p* |  | *Mean difference in change*  *(95% CI)^b^*  *p* |  | *Cohen’s d^c^* |
| T0 |  | 4.3  (3.6, 5.0) |  | - |  | 3.6  (3.1, 4.2) |  | - |  | - |  | - |
| T1 |  | 3.8  (3.1, 4.5) |  | -0.50  (-1.2, 0.25)  .19 |  | 3.0  (2.4, 3.6) |  | -0.61  (-1.3, 0.06)  .08 |  | 0.11  (-0.90, 1.1)  .83 |  | 0.05 |
| ^a^Means, SDs, difference in mean change are based on coefficients and combination of coefficients from mixed effects model following multiple imputation.  ^b^Positive mean difference in change in this table means that the *decrease* in PSC score was larger in PYA than EASE  ^c^ Cohen’s d effect size was calculated by dividing the predicted difference in mean change from the mixed effects model by the pooled baseline SD (2.1).  ^*^Model included fixed effects of arm, time and arm X time interaction, and random effects of pt_code, family ID, and Ease group.  T0=Baseline, T1=Endline | | | | | | | | | | | | |

### 4.4i Child Psychological Symptoms (PSC): Externalizing Subscale: by Caregiver K6 Severity

| **Table 4.4ii Predicted means, mean changes, and effect sizes for Child PSC Externalizing outcome with imputed data among lower 50% Caregiver K6 Scores** | | | | | | | | | | | | |
| --- | --- | --- | --- | --- | --- | --- | --- | --- | --- | --- | --- | --- |
|  |  | **EASE (N=45)** | | |  | **PYA (N=48)** | | |  | **Between group treatment effect** | | |
|  |  | *Mean*  *(95% CI)^a^* |  | *Mean change from baseline*  *(95% CI)*  *p* |  | *Mean*  *(95% CI)^a^* |  | *Mean change from baseline*  *(95% CI)*  *p* |  | *Mean difference in change*  *(95% CI)^b^*  *p* |  | *Cohen’s d^c^* |
| T0 |  | 3.5  (2.8, 4.1) |  | - |  | 3.7  (3.1, 4.3) |  | - |  | - |  | - |
| T1 |  | 3.1  (2.5, 3.8) |  | -0.36  (-1.0, 0.33)  .30 |  | 2.8  (2.1, 3.5) |  | -0.90  (-1.6, -0.18)  **.01** |  | 0.54  (-0.45, 1.5)  .29 |  | 0.28 |
| ^a^Means, SDs, difference in mean change are based on coefficients and combination of coefficients from mixed effects model following multiple imputation.  ^b^Positive mean difference in change in this table means that the *decrease* in PSC score was larger in PYA than EASE  ^c^ Cohen’s d effect size was calculated by dividing the predicted difference in mean change from the mixed effects model by the pooled baseline SD (1.9).  ^*^Model included fixed effects of arm, time and arm X time interaction, and random effects of pt_code, family ID, and Ease group.  T0=Baseline, T1=Endline | | | | | | | | | | | | |

| **Table 4.4iii Predicted means, mean changes, and effect sizes for Child PSC Externalizing outcome with imputed data among upper 50% Caregiver K6 Scores** | | | | | | | | | | | | |
| --- | --- | --- | --- | --- | --- | --- | --- | --- | --- | --- | --- | --- |
|  |  | **EASE (N=35)** | | |  | **PYA (N=70)** | | |  | **Between group treatment effect** | | |
|  |  | *Mean*  *(95% CI)^a^* |  | *Mean change from baseline*  *(95% CI)*  *p* |  | *Mean*  *(95% CI)^a^* |  | *Mean change from baseline*  *(95% CI)*  *p* |  | *Mean difference in change*  *(95% CI)^b^*  *p* |  | *Cohen’s d^c^* |
| T0 |  | 3.7  (3.0, 4.4) |  | - |  | 3.7  (3.1, 4.2) |  | - |  | - |  | - |
| T1 |  | 3.1  (2.4, 3.9) |  | -0.60  (-1.5, 0.26)  .17 |  | 3.0  (2.5, 3.6) |  | -0.62  (-1.2, -0.02)  **.04** |  | 0.03  (-1.0, 1.1)  .95 |  | 0.01 |
| ^a^Means, SDs, difference in mean change are based on coefficients and combination of coefficients from mixed effects model following multiple imputation.  ^b^Positive mean difference in change in this table means that the *decrease* in PSC score was larger in PYA than EASE  ^c^ Cohen’s d effect size was calculated by dividing the predicted difference in mean change from the mixed effects model by the pooled baseline SD (2.1).  ^*^Model included fixed effects of arm, time and arm X time interaction, and random effects of pt_code, family ID, and Ease group.  T0=Baseline, T1=Endline | | | | | | | | | | | | |

### 4.4j Child Psychological Symptoms (PSC): Externalizing Subscale: by Treatment Completer Status

| **Table 4.4ji Predicted means, mean changes, and effect sizes for Child PSC Externalizing outcome with imputed data among treatment completers** | | | | | | | | | | | | |
| --- | --- | --- | --- | --- | --- | --- | --- | --- | --- | --- | --- | --- |
|  |  | **EASE (N=56)** | | |  | **PYA (N=117)** | | |  | **Between group treatment effect** | | |
|  |  | *Mean*  *(95% CI)^a^* |  | *Mean change from baseline*  *(95% CI)*  *p* |  | *Mean*  *(95% CI)^a^* |  | *Mean change from baseline*  *(95% CI)*  *p* |  | *Mean difference in change*  *(95% CI)^b^*  *p* |  | *Cohen’s d^c^* |
| T0 |  | 3.5  (2.9, 4.2) |  | - |  | 3.7  (2.3, 5.0) |  | - |  | - |  | - |
| T1 |  | 3.1  (2.4, 3.8) |  | -0.41  (-1.1, 0.24)  .22 |  | 3.0  (1.6, 4.3) |  | -0.73  (-1.2, -0.27)  **<.01** |  | 0.33  (-0.47, 1.1)  .43 |  | 0.17 |
| ^a^Means, SDs, difference in mean change are based on coefficients and combination of coefficients from mixed effects model following multiple imputation.  ^b^Positive mean difference in change in this table means that the *decrease* in PSC score was larger in PYA than EASE  ^c^ Cohen’s d effect size was calculated by dividing the predicted difference in mean change from the mixed effects model by the pooled baseline SD (2.0).  ^*^Model included fixed effects of arm, time and arm X time interaction, and random effects of pt_code, family ID, and Ease group.  T0=Baseline, T1=Endline | | | | | | | | | | | | |

## Child PHQ

### 4.5a Child PHQ: by Gender

| **Table 4.5ai Predicted means, mean changes, and effect sizes for Child PHQ outcome with imputed data among MALES** | | | | | | | | | | | | |
| --- | --- | --- | --- | --- | --- | --- | --- | --- | --- | --- | --- | --- |
|  |  | **EASE (N=43)** | | |  | **PYA (N=58)** | | |  | **Between group treatment effect** | | |
|  |  | *Mean*  *(95% CI)^a^* |  | *Mean change from baseline*  *(95% CI)*  *p* |  | *Mean*  *(95% CI)^a^* |  | *Mean change from baseline*  *(95% CI)*  *p* |  | *Mean difference in change*  *(95% CI)^b^*  *p* |  | *Cohen’s d^c^* |
| T0 |  | 9.8  (7.9, 11.7) |  | - |  | 8.2  (6.6, 9.9) |  | - |  | - |  | - |
| T1 |  | 8.9  (7.0, 10.9) |  | -0.84  (-2.8, 1.1)  .39 |  | 6.9  (5.3, 8.6) |  | -1.3  (-2.9, 0.38)  .13 |  | 0.43  (-2.1, 3.0)  .74 |  | 0.07 |
| ^a^Means, SDs, difference in mean change are based on coefficients and combination of coefficients from mixed effects model following multiple imputation.  ^b^Positive mean difference in change in this table means that the *decrease* in PHQ score was larger in PYA than EASE  ^c^ Cohen’s d effect size was calculated by dividing the predicted difference in mean change from the mixed effects model by the pooled baseline SD (6.6).  ^*^Model included fixed effects of arm, time and arm X time interaction, and random effects of pt_code, family ID, and Ease group.  T0=Baseline, T1=Endline | | | | | | | | | | | | |

| **Table 4.5aii Predicted means, mean changes, and effect sizes for Child PHQ outcome with imputed data among FEMALES** | | | | | | | | | | | | |
| --- | --- | --- | --- | --- | --- | --- | --- | --- | --- | --- | --- | --- |
|  |  | **EASE (N=37)** | | |  | **PYA (N=60)** | | |  | **Between group treatment effect** | | |
|  |  | *Mean*  *(95% CI)^a^* |  | *Mean change from baseline*  *(95% CI)*  *p* |  | *Mean*  *(95% CI)^a^* |  | *Mean change from baseline*  *(95% CI)*  *p* |  | *Mean difference in change*  *(95% CI)^b^*  *p* |  | *Cohen’s d^c^* |
| T0 |  | 9.1  (6.4, 11.8) |  | - |  | 11.1  (6.9, 15.3) |  | - |  | - |  | - |
| T1 |  | 7.6  (4.9, 1.04) |  | -1.5  (-3.6, 0.60)  .16 |  | 9.9  (5.7, 14.1) |  | -1.2  (-2.9, 0.44)  .15 |  | -0.28  (-3.0, 2.4)  .84 |  | 0.04 |
| ^a^Means, SDs, difference in mean change are based on coefficients and combination of coefficients from mixed effects model following multiple imputation.  ^b^Negative mean difference in change in this table means that the *decrease* in PHQ score was larger in EASE than PYA  ^c^ Cohen’s d effect size was calculated by dividing the predicted difference in mean change from the mixed effects model by the pooled baseline SD (6.6).  ^*^Model included fixed effects of arm, time and arm X time interaction, and random effects of pt_code, family ID, and Ease group.  T0=Baseline, T1=Endline | | | | | | | | | | | | |

### 4.5b Child PHQ: by Age

| **Table 4.5bi Predicted means, mean changes, and effect sizes for Child PHQ outcome with imputed data among 10-12 year olds** | | | | | | | | | | | | |
| --- | --- | --- | --- | --- | --- | --- | --- | --- | --- | --- | --- | --- |
|  |  | **EASE (N=56)** | | |  | **PYA (N=77)** | | |  | **Between group treatment effect** | | |
|  |  | *Mean*  *(95% CI)^a^* |  | *Mean change from baseline*  *(95% CI)*  *p* |  | *Mean*  *(95% CI)^a^* |  | *Mean change from baseline*  *(95% CI)*  *p* |  | *Mean difference in change*  *(95% CI)^b^*  *p* |  | *Cohen’s d^c^* |
| T0 |  | 8.4  (6.8, 10.0) |  | - |  | 9.0  (7.0, 11.1) |  | - |  | - |  | - |
| T1 |  | 7.5  (5.8, 9-1) |  | -0.94  (-2.6, 0.68)  .25 |  | 7.9  (5.9, 10.0) |  | -1.1  (-2.5, 0.29)  .12 |  | 0.17  (-2.0, 2.3)  .88 |  | 0.02 |
| ^a^Means, SDs, difference in mean change are based on coefficients and combination of coefficients from mixed effects model following multiple imputation.  ^b^Positive mean difference in change in this table means that the *decrease* in PHQ score was larger in EASE than PYA  ^c^ Cohen’s d effect size was calculated by dividing the predicted difference in mean change from the mixed effects model by the pooled baseline SD (8.8).  ^*^Model included fixed effects of arm, time and arm X time interaction, and random effects of pt_code, family ID, and Ease group.  T0=Baseline, T1=Endline | | | | | | | | | | | | |

| **Table 4.5bii Predicted means, mean changes, and effect sizes for Child PHQ outcome with imputed data among 13-14 year olds** | | | | | | | | | | | | |
| --- | --- | --- | --- | --- | --- | --- | --- | --- | --- | --- | --- | --- |
|  |  | **EASE (N=24)** | | |  | **PYA (N=41)** | | |  | **Between group treatment effect** | | |
|  |  | *Mean*  *(95% CI)^a^* |  | *Mean change from baseline*  *(95% CI)*  *p* |  | *Mean*  *(95% CI)^a^* |  | *Mean change from baseline*  *(95% CI)*  *p* |  | *Mean difference in change*  *(95% CI)^b^*  *p* |  | *Cohen’s d^c^* |
| T0 |  | 11.3  (7.8, 14.7) |  | - |  | 10.6  (4.9, 16.4) |  | - |  | - |  | - |
| T1 |  | 9.7  (6.1, 13.2) |  | -1.6  (-4.3, 1.1)  .25 |  | 9.1  (3.3, 14.9( |  | **-**1.5  (-3.6, 0.60)  .16 |  | -0.11  (-3.6, 3.3)  .95 |  | 0.02 |
| ^a^Means, SDs, difference in mean change are based on coefficients and combination of coefficients from mixed effects model following multiple imputation.  ^b^Negative mean difference in change in this table means that the *decrease* in PHQ score was larger in EASE than PYA  ^c^ Cohen’s d effect size was calculated by dividing the predicted difference in mean change from the mixed effects model by the pooled baseline SD (7.2).  ^*^Model included fixed effects of arm, time and arm X time interaction, and random effects of pt_code, family ID, and Ease group.  T0=Baseline, T1=Endline | | | | | | | | | | | | |

### 4.5c Child PHQ: by PSC Severity

| **Table 4.5ci Predicted means, mean changes, and effect sizes for Child PHQ outcome with imputed data among lower 50% PSC Scores** | | | | | | | | | | | | |
| --- | --- | --- | --- | --- | --- | --- | --- | --- | --- | --- | --- | --- |
|  |  | **EASE (N=37)** | | |  | **PYA (N=59)** | | |  | **Between group treatment effect** | | |
|  |  | *Mean*  *(95% CI)^a^* |  | *Mean change from baseline*  *(95% CI)*  *p* |  | *Mean*  *(95% CI)^a^* |  | *Mean change from baseline*  *(95% CI)*  *p* |  | *Mean difference in change*  *(95% CI)^b^*  *p* |  | *Cohen’s d^c^* |
| T0 |  | 5.4  (3.8, 7.1) |  | - |  | 5.8  (4.4, 7.1) |  | - |  | - |  | - |
| T1 |  | 4.0  (2.3, 5.8) |  | -1.4  (-3.4, 0.54)  .16 |  | 6.5  (5.2, 7.9) |  | 0.75  (-0.76, 2.3)  .33 |  | -2.2  (-4.6, 0.30)  .09 |  | 0.44 |
| ^a^Means, SDs, difference in mean change are based on coefficients and combination of coefficients from mixed effects model following multiple imputation.  ^b^Negative mean difference in change in this table means that the *decrease* in PHQ score was larger in EASE than PYA  ^c^ Cohen’s d effect size was calculated by dividing the predicted difference in mean change from the mixed effects model by the pooled baseline SD (5.0).  ^*^Model included fixed effects of arm, time and arm X time interaction, and random effects of pt_code, family ID, and Ease group.  T0=Baseline, T1=Endline | | | | | | | | | | | | |

| **Table 4.5cii Predicted means, mean changes, and effect sizes for Child PHQ outcome with imputed data among upper 50% PSC Scores** | | | | | | | | | | | | |
| --- | --- | --- | --- | --- | --- | --- | --- | --- | --- | --- | --- | --- |
|  |  | **EASE (N=43)** | | |  | **PYA (N=59)** | | |  | **Between group treatment effect** | | |
|  |  | *Mean*  *(95% CI)^a^* |  | *Mean change from baseline*  *(95% CI)*  *p* |  | *Mean*  *(95% CI)^a^* |  | *Mean change from baseline*  *(95% CI)*  *p* |  | *Mean difference in change*  *(95% CI)^b^*  *p* |  | *Cohen’s d^c^* |
| T0 |  | 13.0  (11.1, 14.8) |  | - |  | 13.5  (11.8, 15.1) |  | - |  | - |  | - |
| T1 |  | 12.0  (10.1, 14.0) |  | -0.91  (-2.9, 1.1)  .37 |  | 10.2  (8.5, 11.9) |  | -3.2  (-4.9, -1.5)  **<.0001** |  | 2.3  (-0.28, 4.9)  .08 |  | 0.40 |
| ^a^Means, SDs, difference in mean change are based on coefficients and combination of coefficients from mixed effects model following multiple imputation.  ^b^Positive mean difference in change in this table means that the *decrease* in PHQ score was larger in PYA than EASE  ^c^ Cohen’s d effect size was calculated by dividing the predicted difference in mean change from the mixed effects model by the pooled baseline SD (5.8).  ^*^Model included fixed effects of arm, time and arm X time interaction, and random effects of pt_code, family ID, and Ease group.  T0=Baseline, T1=Endline | | | | | | | | | | | | |

### 4.5d Child PHQ: by PSC Internalizing Severity

| **Table 4.5di Predicted means, mean changes, and effect sizes for Child PHQ outcome with imputed data among lower 50% PSC Internalizing Scores** | | | | | | | | | | | | |
| --- | --- | --- | --- | --- | --- | --- | --- | --- | --- | --- | --- | --- |
|  |  | **EASE (N=43)** | | |  | **PYA (N=66)** | | |  | **Between group treatment effect** | | |
|  |  | *Mean*  *(95% CI)^a^* |  | *Mean change from baseline*  *(95% CI)*  *p* |  | *Mean*  *(95% CI)^a^* |  | *Mean change from baseline*  *(95% CI)*  *p* |  | *Mean difference in change*  *(95% CI)^b^*  *p* |  | *Cohen’s d^c^* |
| T0 |  | 8.5  (6.8, 10.2) |  | - |  | 7.3  (5.9, 8.7) |  | - |  | - |  | - |
| T1 |  | 6.5  (4.7, 8.3) |  | -2.0  (-3.9, -0.10)  **.04** |  | 6.9  (5.5, 8.4) |  | -0.35  (-1.9, 1.2)  .65 |  | -1.7  (-4.1, 0.78)  .18 |  | 0.28 |
| ^a^Means, SDs, difference in mean change are based on coefficients and combination of coefficients from mixed effects model following multiple imputation.  ^b^Negative mean difference in change in this table means that the *decrease* in PHQ score was larger in EASE than PYA  ^c^ Cohen’s d effect size was calculated by dividing the predicted difference in mean change from the mixed effects model by the pooled baseline SD (6.0).  ^*^Model included fixed effects of arm, time and arm X time interaction, and random effects of pt_code, family ID, and Ease group.  T0=Baseline, T1=Endline | | | | | | | | | | | | |

| **Table 4.5dii Predicted means, mean changes, and effect sizes for Child PHQ outcome with imputed data among upper 50% PSC Internalizing Scores** | | | | | | | | | | | | |
| --- | --- | --- | --- | --- | --- | --- | --- | --- | --- | --- | --- | --- |
|  |  | **EASE (N=37)** | | |  | **PYA (N=52)** | | |  | **Between group treatment effect** | | |
|  |  | *Mean*  *(95% CI)^a^* |  | *Mean change from baseline*  *(95% CI)*  *p* |  | *Mean*  *(95% CI)^a^* |  | *Mean change from baseline*  *(95% CI)*  *p* |  | *Mean difference in change*  *(95% CI)^b^*  *p* |  | *Cohen’s d^c^* |
| T0 |  | 10.1  (7.7, 12.6) |  | - |  | 12.5  (8.0, 16.9) |  | - |  | - |  | - |
| T1 |  | 10.0  (7.5, 12.5) |  | -0.12  (-2.2, 1.9)  .91 |  | 10.1  (5.6, 14.6) |  | -2.3  (-4.2, -0.54)  **.01** |  | 2.3  (-0.51, 5.0)  .11 |  | 0.34 |
| ^a^Means, SDs, difference in mean change are based on coefficients and combination of coefficients from mixed effects model following multiple imputation.  ^b^Negative mean difference in change in this table means that the *decrease* in PHQ score was larger in EASE than PYA  ^c^ Cohen’s d effect size was calculated by dividing the predicted difference in mean change from the mixed effects model by the pooled baseline SD (6.8).  ^*^Model included fixed effects of arm, time and arm X time interaction, and random effects of pt_code, family ID, and Ease group.  T0=Baseline, T1=Endline | | | | | | | | | | | | |

### 4.5e Child PHQ: by PSC Externalizing Severity

| **Table 4.5ei Predicted means, mean changes, and effect sizes for Child PHQ outcome with imputed data among lower 50% PSC Externalizing Scores** | | | | | | | | | | | | |
| --- | --- | --- | --- | --- | --- | --- | --- | --- | --- | --- | --- | --- |
|  |  | **EASE (N=39)** | | |  | **PYA (N=56)** | | |  | **Between group treatment effect** | | |
|  |  | *Mean*  *(95% CI)^a^* |  | *Mean change from baseline*  *(95% CI)*  *p* |  | *Mean*  *(95% CI)^a^* |  | *Mean change from baseline*  *(95% CI)*  *p* |  | *Mean difference in change*  *(95% CI)^b^*  *p* |  | *Cohen’s d^c^* |
| T0 |  | 8.4  (6.2, 10.5) |  | - |  | 9.9  (7.0, 12.8) |  | - |  | - |  | - |
| T1 |  | 6.6  (4.4, 8.8) |  | -1.8  (-3.8, 0.30)  .09 |  | 8.5  (5.6, 11.5) |  | -1.3  (-3.0, 0.49)  .13 |  | -0.45  (-3.1, 2.2)  .74 |  | 0.07 |
| ^a^Means, SDs, difference in mean change are based on coefficients and combination of coefficients from mixed effects model following multiple imputation.  ^b^Negative mean difference in change in this table means that the *decrease* in PHQ score was larger in EASE than PYA  ^c^ Cohen’s d effect size was calculated by dividing the predicted difference in mean change from the mixed effects model by the pooled baseline SD (6.8).  ^*^Model included fixed effects of arm, time and arm X time interaction, and random effects of pt_code, family ID, and Ease group.  T0=Baseline, T1=Endline | | | | | | | | | | | | |

| **Table 4.5eii Predicted means, mean changes, and effect sizes for Child PHQ outcome with imputed data among upper 50% PSC Externalizing Scores** | | | | | | | | | | | | |
| --- | --- | --- | --- | --- | --- | --- | --- | --- | --- | --- | --- | --- |
|  |  | **EASE (N=41)** | | |  | **PYA (N=62)** | | |  | **Between group treatment effect** | | |
|  |  | *Mean*  *(95% CI)^a^* |  | *Mean change from baseline*  *(95% CI)*  *p* |  | *Mean*  *(95% CI)^a^* |  | *Mean change from baseline*  *(95% CI)*  *p* |  | *Mean difference in change*  *(95% CI)^b^*  *p* |  | *Cohen’s d^c^* |
| T0 |  | 10.3  (8.4, 12.3) |  | - |  | 9.4  (7.8, 11.1) |  | - |  | - |  | - |
| T1 |  | 9.8  (7.8, 11.8) |  | -0.54  (-2.5, 1.4)  .59 |  | 8.3  (6.6, 9.9) |  | -1.2  (-2.7, 0.41)  .15 |  | 0.63  (-1.9, 3.1)  .62 |  | 0.10 |
| ^a^Means, SDs, difference in mean change are based on coefficients and combination of coefficients from mixed effects model following multiple imputation.  ^b^Positive mean difference in change in this table means that the *decrease* in PHQ score was larger in PYA than EASE  ^c^ Cohen’s d effect size was calculated by dividing the predicted difference in mean change from the mixed effects model by the pooled baseline SD (6.6).  ^*^Model included fixed effects of arm, time and arm X time interaction, and random effects of pt_code, family ID, and Ease group.  T0=Baseline, T1=Endline | | | | | | | | | | | | |

### 4.5f Child PHQ: by PHQ Severity

| **Table 4.5fi Predicted means, mean changes, and effect sizes for Child PHQ outcome with imputed data among lower 50% PHQ Scores** | | | | | | | | | | | | |
| --- | --- | --- | --- | --- | --- | --- | --- | --- | --- | --- | --- | --- |
|  |  | **EASE (N=40)** | | |  | **PYA (N=57)** | | |  | **Between group treatment effect** | | |
|  |  | *Mean*  *(95% CI)^a^* |  | *Mean change from baseline*  *(95% CI)*  *p* |  | *Mean*  *(95% CI)^a^* |  | *Mean change from baseline*  *(95% CI)*  *p* |  | *Mean difference in change*  *(95% CI)^b^*  *p* |  | *Cohen’s d^c^* |
| T0 |  | 4.3  (2.9, 5.8) |  | - |  | 4.0  (2.7, 5.3) |  | - |  | - |  | - |
| T1 |  | 4.1  (2.6, 5.7) |  | -0.21  (-2.0, 1.5)  .82 |  | 6.5  (5.2, 7.8) |  | 2.5  (1.1, 3.9)  **<.01** |  | -2.7  (-5.0, -0.42)  **.02** |  | 0.93 |
| ^a^Means, SDs, difference in mean change are based on coefficients and combination of coefficients from mixed effects model following multiple imputation.  ^b^Negative mean difference in change in this table means that the *decrease* in PHQ score was larger in EASE than PYA  ^c^ Cohen’s d effect size was calculated by dividing the predicted difference in mean change from the mixed effects model by the pooled baseline SD (2.9).  ^*^Model included fixed effects of arm, time and arm X time interaction, and random effects of pt_code, family ID, and Ease group.  T0=Baseline, T1=Endline | | | | | | | | | | | | |

| **Table 4.5fii Predicted means, mean changes, and effect sizes for Child PHQ outcome with imputed data among upper 50% PHQ Scores** | | | | | | | | | | | | |
| --- | --- | --- | --- | --- | --- | --- | --- | --- | --- | --- | --- | --- |
|  |  | **EASE (N=40)** | | |  | **PYA (N=61)** | | |  | **Between group treatment effect** | | |
|  |  | *Mean*  *(95% CI)^a^* |  | *Mean change from baseline*  *(95% CI)*  *p* |  | *Mean*  *(95% CI)^a^* |  | *Mean change from baseline*  *(95% CI)*  *p* |  | *Mean difference in change*  *(95% CI)^b^*  *p* |  | *Cohen’s d^c^* |
| T0 |  | 14.7  (12.5, 16.9) |  | - |  | 15.0  (10.9, 19.0) |  | - |  | - |  | - |
| T1 |  | 12.6  (10.4, 14.8) |  | -2.1  (-4.1, -0.06)  **.04** |  | 10.3  (6.2, 14.3) |  | -4.7  (-6.4, -3.1)  **<.0001** |  | 2.7  (0.08, 5.3)  **.04** |  | 0.59 |
| ^a^Means, SDs, difference in mean change are based on coefficients and combination of coefficients from mixed effects model following multiple imputation.  ^b^Positive mean difference in change in this table means that the *decrease* in PHQ score was larger in PYA than EASE  ^c^ Cohen’s d effect size was calculated by dividing the predicted difference in mean change from the mixed effects model by the pooled baseline SD (4.6).  ^*^Model included fixed effects of arm, time and arm X time interaction, and random effects of pt_code, family ID, and Ease group.  T0=Baseline, T1=Endline | | | | | | | | | | | | |

### 4.5g Child PHQ: by Child Wellbeing Severity

| **Table 4.5gi Predicted means, mean changes, and effect sizes for Child PHQ outcome with imputed data among lower 50% Child Wellbeing Scores** | | | | | | | | | | | | |
| --- | --- | --- | --- | --- | --- | --- | --- | --- | --- | --- | --- | --- |
|  |  | **EASE (N=36)** | | |  | **PYA (N=58)** | | |  | **Between group treatment effect** | | |
|  |  | *Mean*  *(95% CI)^a^* |  | *Mean change from baseline*  *(95% CI)*  *p* |  | *Mean*  *(95% CI)^a^* |  | *Mean change from baseline*  *(95% CI)*  *p* |  | *Mean difference in change*  *(95% CI)^b^*  *p* |  | *Cohen’s d^c^* |
| T0 |  | 12.2  (9.9, 14.4) |  | - |  | 11.1  (8.3, 13.8) |  | - |  | - |  | - |
| T1 |  | 11.1  (8.8, 13.4) |  | -1.0  (-3.1, 1.0)  .33 |  | 9.0  (6.2, 11.8) |  | -2.1  (-3.7, -0.45)  **.01** |  | 1.0  (-1.6, 3.7)  .44 |  | 0.14 |
| ^a^Means, SDs, difference in mean change are based on coefficients and combination of coefficients from mixed effects model following multiple imputation.  ^b^Positive mean difference in change in this table means that the *decrease* in PHQ score was larger in PYA than EASE  ^c^ Cohen’s d effect size was calculated by dividing the predicted difference in mean change from the mixed effects model by the pooled baseline SD (7.0).  ^*^Model included fixed effects of arm, time and arm X time interaction, and random effects of pt_code, family ID, and Ease group.  T0=Baseline, T1=Endline | | | | | | | | | | | | |

| **Table 4.5gii Predicted means, mean changes, and effect sizes for Child PHQ outcome with imputed data among upper 50% Child Wellbeing Scores** | | | | | | | | | | | | |
| --- | --- | --- | --- | --- | --- | --- | --- | --- | --- | --- | --- | --- |
|  |  | **EASE (N=44)** | | |  | **PYA (N=60)** | | |  | **Between group treatment effect** | | |
|  |  | *Mean*  *(95% CI)^a^* |  | *Mean change from baseline*  *(95% CI)*  *p* |  | *Mean*  *(95% CI)^a^* |  | *Mean change from baseline*  *(95% CI)*  *p* |  | *Mean difference in change*  *(95% CI)^b^*  *p* |  | *Cohen’s d^c^* |
| T0 |  | 7.2  (5.2, 9.2) |  | - |  | 8.1  (4.4, 11.8) |  | - |  | - |  | - |
| T1 |  | 6.0  (3.9, 8.0) |  | -1.2  (-3.1, 0.69)  .21 |  | 7.7  (4.0, 11.4) |  | -0.44  (-2.1, 1.2)  .61 |  | -0.79  (-3.3, 1.8)  .55 |  | 0.14 |
| ^a^Means, SDs, difference in mean change are based on coefficients and combination of coefficients from mixed effects model following multiple imputation.  ^b^Negative mean difference in change in this table means that the *decrease* in PHQ score was larger in EASE than PYA  ^c^ Cohen’s d effect size was calculated by dividing the predicted difference in mean change from the mixed effects model by the pooled baseline SD (5.8).  ^*^Model included fixed effects of arm, time and arm X time interaction, and random effects of pt_code, family ID, and Ease group.  T0=Baseline, T1=Endline | | | | | | | | | | | | |

### 4.5h Child PHQ: by Caregiver PSC Severity

| **Table 4.5hi Predicted means, mean changes, and effect sizes for Child PHQ outcome with imputed data among lower 50% Caregiver PSC Scores** | | | | | | | | | | | | |
| --- | --- | --- | --- | --- | --- | --- | --- | --- | --- | --- | --- | --- |
|  |  | **EASE (N=36)** | | |  | **PYA (N=59)** | | |  | **Between group treatment effect** | | |
|  |  | *Mean*  *(95% CI)^a^* |  | *Mean change from baseline*  *(95% CI)*  *p* |  | *Mean*  *(95% CI)^a^* |  | *Mean change from baseline*  *(95% CI)*  *p* |  | *Mean difference in change*  *(95% CI)^b^*  *p* |  | *Cohen’s d^c^* |
| T0 |  | 7.4  (5.2, 9.6) |  | - |  | 8.0  (4.9, 11.1) |  | - |  | - |  | - |
| T1 |  | 6.2  (3.9, 8.4) |  | -1.2  (-3.4, 0.91)  .26 |  | 7.3  (4.2, 10.4) |  | -0.76  (-2.4, 0.91)  .37 |  | -0.47  (-3.2, 2.3)  .74 |  | 0.12 |
| ^a^Means, SDs, difference in mean change are based on coefficients and combination of coefficients from mixed effects model following multiple imputation.  ^b^Negative mean difference in change in this table means that the *decrease* in PHQ score was larger in EASE than PYA  ^c^ Cohen’s d effect size was calculated by dividing the predicted difference in mean change from the mixed effects model by the pooled baseline SD (6.1).  ^*^Model included fixed effects of arm, time and arm X time interaction, and random effects of pt_code, family ID, and Ease group.  T0=Baseline, T1=Endline | | | | | | | | | | | | |

| **Table 4.5hii Predicted means, mean changes, and effect sizes for Child PHQ outcome with imputed data among upper 50% Caregiver PSC Scores** | | | | | | | | | | | | |
| --- | --- | --- | --- | --- | --- | --- | --- | --- | --- | --- | --- | --- |
|  |  | **EASE (N=44)** | | |  | **PYA (N=59)** | | |  | **Between group treatment effect** | | |
|  |  | *Mean*  *(95% CI)^a^* |  | *Mean change from baseline*  *(95% CI)*  *p* |  | *Mean*  *(95% CI)^a^* |  | *Mean change from baseline*  *(95% CI)*  *p* |  | *Mean difference in change*  *(95% CI)^b^*  *p* |  | *Cohen’s d^c^* |
| T0 |  | 11.3  (9.4, 13.1) |  | - |  | 11.2  (9.5, 12.8) |  | - |  | - |  | - |
| T1 |  | 10.2  (8.2, 12.1) |  | -1.1  (-3.0, 0.83)  .27 |  | 9.4  (7.7, 11.1) |  | -1.7  (-3.4, -0.09)  **.04** |  | 0.66  (-1.9, 3.2)  .61 |  | 0.10 |
| ^a^Means, SDs, difference in mean change are based on coefficients and combination of coefficients from mixed effects model following multiple imputation.  ^b^Positive mean difference in change in this table means that the *decrease* in PHQ score was larger in PYA than EASE  ^c^ Cohen’s d effect size was calculated by dividing the predicted difference in mean change from the mixed effects model by the pooled baseline SD (6.7).  ^*^Model included fixed effects of arm, time and arm X time interaction, and random effects of pt_code, family ID, and Ease group.  T0=Baseline, T1=Endline | | | | | | | | | | | | |

### 4.5i Child PHQ: by Caregiver K6 Severity

| **Table 4.5ii Predicted means, mean changes, and effect sizes for Child PHQ outcome with imputed data among lower 50% Caregiver PSC Scores** | | | | | | | | | | | | |
| --- | --- | --- | --- | --- | --- | --- | --- | --- | --- | --- | --- | --- |
|  |  | **EASE (N=45)** | | |  | **PYA (N=48)** | | |  | **Between group treatment effect** | | |
|  |  | *Mean*  *(95% CI)^a^* |  | *Mean change from baseline*  *(95% CI)*  *p* |  | *Mean*  *(95% CI)^a^* |  | *Mean change from baseline*  *(95% CI)*  *p* |  | *Mean difference in change*  *(95% CI)^b^*  *p* |  | *Cohen’s d^c^* |
| T0 |  | 9.1  (7.1, 11.1) |  | - |  | 8.3  (5.9, 10.8) |  | - |  | - |  | - |
| T1 |  | 8.3  (6.3, 10.4) |  | -0.76  (-2.7, 1.2)  .44 |  | 7.0  (4.4, 9.5) |  | -1.4  (-3.2, 0.44)  .14 |  | 0.64  (-2.0, 3.3)  .64 |  | 0.10 |
| ^a^Means, SDs, difference in mean change are based on coefficients and combination of coefficients from mixed effects model following multiple imputation.  ^b^Positive mean difference in change in this table means that the *decrease* in PHQ score was larger in PYA than EASE  ^c^ Cohen’s d effect size was calculated by dividing the predicted difference in mean change from the mixed effects model by the pooled baseline SD (6.6).  ^*^Model included fixed effects of arm, time and arm X time interaction, and random effects of pt_code, family ID, and Ease group.  T0=Baseline, T1=Endline | | | | | | | | | | | | |

| **Table 4.5iii Predicted means, mean changes, and effect sizes for Child PHQ outcome with imputed data among upper 50% Caregiver PSC Scores** | | | | | | | | | | | | |
| --- | --- | --- | --- | --- | --- | --- | --- | --- | --- | --- | --- | --- |
|  |  | **EASE (N=35)** | | |  | **PYA (N=70)** | | |  | **Between group treatment effect** | | |
|  |  | *Mean*  *(95% CI)^a^* |  | *Mean change from baseline*  *(95% CI)*  *p* |  | *Mean*  *(95% CI)^a^* |  | *Mean change from baseline*  *(95% CI)*  *p* |  | *Mean difference in change*  *(95% CI)^b^*  *p* |  | *Cohen’s d^c^* |
| T0 |  | 10.0  (7.4, 12.6) |  | - |  | 10.5  (5.3, 15.6) |  | - |  | - |  | - |
| T1 |  | 8.4  (5.7, 11.1) |  | -1.7  (-3.9, 0.56)  .14 |  | 9.3  (4.2, 14.5) |  | -1.1  (-2.6, 0.36)  .14 |  | -0.51  (-3.2, 2.2)  .71 |  | 0.08 |
| ^a^Means, SDs, difference in mean change are based on coefficients and combination of coefficients from mixed effects model following multiple imputation.  ^b^Negative mean difference in change in this table means that the *decrease* in PHQ score was larger in EASE than PYA  ^c^ Cohen’s d effect size was calculated by dividing the predicted difference in mean change from the mixed effects model by the pooled baseline SD (6.7).  ^*^Model included fixed effects of arm, time and arm X time interaction, and random effects of pt_code, family ID, and Ease group.  T0=Baseline, T1=Endline | | | | | | | | | | | | |

### 4.5j Child PHQ: by Treatment Completer Status

| **Table 4.5ji Predicted means, mean changes, and effect sizes for Child PHQ outcome with imputed data among treatment completers** | | | | | | | | | | | | |
| --- | --- | --- | --- | --- | --- | --- | --- | --- | --- | --- | --- | --- |
|  |  | **EASE (N=56)** | | |  | **PYA (N=117)** | | |  | **Between group treatment effect** | | |
|  |  | *Mean*  *(95% CI)^a^* |  | *Mean change from baseline*  *(95% CI)*  *p* |  | *Mean*  *(95% CI)^a^* |  | *Mean change from baseline*  *(95% CI)*  *p* |  | *Mean difference in change*  *(95% CI)^b^*  *p* |  | *Cohen’s d^c^* |
| T0 |  | 10.0  (8.3, 11.7) |  | - |  | 9.6  (8.4, 10.9) |  | - |  | - |  | - |
| T1 |  | 8.8  (7.2, 10.5) |  | -1.2  (-2.9, 0.52)  .17 |  | 8.4  (7.1, 9.6) |  | -1.3  (-2.5, -0.07)  **.04** |  | 0.09  (-1.0, 1.1)  .93 |  | 0.01 |
| ^a^Means, SDs, difference in mean change are based on coefficients and combination of coefficients from mixed effects model following multiple imputation.  ^b^Positive mean difference in change in this table means that the *decrease* in PHQ score was larger in PYA than EASE  ^c^ Cohen’s d effect size was calculated by dividing the predicted difference in mean change from the mixed effects model by the pooled baseline SD (6.5).  ^*^Model included fixed effects of arm, time and arm X time interaction, and random effects of pt_code, family ID, and Ease group.  T0=Baseline, T1=Endline | | | | | | | | | | | | |

## Child Wellbeing

### 4.6a Child Wellbeing: by Gender

| **Table 4.6aii Predicted means, mean changes, and effect sizes for Child Wellbeing outcome with imputed data among MALES** | | | | | | | | | | | | |
| --- | --- | --- | --- | --- | --- | --- | --- | --- | --- | --- | --- | --- |
|  |  | **EASE (N=37)** | | |  | **PYA (N=60)** | | |  | **Between group treatment effect** | | |
|  |  | *Mean*  *(95% CI)^a^* |  | *Mean change from baseline*  *(95% CI)*  *p* |  | *Mean*  *(95% CI)^a^* |  | *Mean change from baseline*  *(95% CI)*  *p* |  | *Mean difference in change*  *(95% CI)^b^*  *p* |  | *Cohen’s d^c^* |
| T0 |  | 43.9  (40.8, 47.0) |  | - |  | 44.7  (42.0, 47.4) |  | - |  | - |  | - |
| T1 |  | 45.2  (42.0, 48.4) |  | 1.4  (-2.3, 5.0)  .46 |  | 43.3  (40.5, 46.1) |  | -1.4  (-4.6, 1.8)  .41 |  | 2.7  (-2.1, 7.5)  .27 |  | 0.24 |
| ^a^Means, SDs, difference in mean change are based on coefficients and combination of coefficients from mixed effects model following multiple imputation.  ^b^Positive mean difference in change in this table means that the *increase* in Wellbeing score was larger in EASE than PYA  ^c^ Cohen’s d effect size was calculated by dividing the predicted difference in mean change from the mixed effects model by the pooled baseline SD (11.1).  ^*^Model included fixed effects of arm, time and arm X time interaction, and random effects of pt_code, family ID, and Ease group.  T0=Baseline, T1=Endline | | | | | | | | | | | | |

| **Table 4.6ai Predicted means, mean changes, and effect sizes for Child PSC outcome with imputed data among FEMALES** | | | | | | | | | | | | |
| --- | --- | --- | --- | --- | --- | --- | --- | --- | --- | --- | --- | --- |
|  |  | **EASE (N=43)** | | |  | **PYA (N=58)** | | |  | **Between group treatment effect** | | |
|  |  | *Mean*  *(95% CI)^a^* |  | *Mean change from baseline*  *(95% CI)*  *p* |  | *Mean*  *(95% CI)^a^* |  | *Mean change from baseline*  *(95% CI)*  *p* |  | *Mean difference in change*  *(95% CI)^b^*  *p* |  | *Cohen’s d^c^* |
| T0 |  | 45.6  (42.2, 4.1) |  | - |  | 43.9  (41.2, 46.6) |  | - |  | - |  | - |
| T1 |  | 45.9  (42.4, 49.5) |  | 0.29  (-3.7, 4.3)  .89 |  | 44.7  (41.9, 47.5) |  | 0.78  (-2.4, 3.9)  .63 |  | -0.49  (-5.6, 4.6)  .85 |  | 0.04 |
| ^a^Means, SDs, difference in mean change are based on coefficients and combination of coefficients from mixed effects model following multiple imputation.  ^b^Negative mean difference in change in this table means that the *increase* in Wellbeing score was smaller in EASE than PYA  ^c^ Cohen’s d effect size was calculated by dividing the predicted difference in mean change from the mixed effects model by the pooled baseline SD (11.5).  ^*^Model included fixed effects of arm, time and arm X time interaction, and random effects of pt_code, family ID, and Ease group.  T0=Baseline, T1=Endline | | | | | | | | | | | | |

### 4.6b Child Wellbeing: by Age

| **Table 4.6bi Predicted means, mean changes, and effect sizes for Child Wellbeing outcome with imputed data among 10-12 year olds** | | | | | | | | | | | | |
| --- | --- | --- | --- | --- | --- | --- | --- | --- | --- | --- | --- | --- |
|  |  | **EASE (N=56)** | | |  | **PYA (N=77)** | | |  | **Between group treatment effect** | | |
|  |  | *Mean*  *(95% CI)^a^* |  | *Mean change from baseline*  *(95% CI)*  *p* |  | *Mean*  *(95% CI)^a^* |  | *Mean change from baseline*  *(95% CI)*  *p* |  | *Mean difference in change*  *(95% CI)^b^*  *p* |  | *Cohen’s d^c^* |
| T0 |  | 45.4  (42.7, 48.1) |  | - |  | 46.0  (43.7, 48.3) |  | - |  | - |  | - |
| T1 |  | 46.0  (43.2, 48.7) |  | 0.52  (-2.7, 3.8)  .76 |  | 45.6  (43.2, 48.0) |  | -0.46  (-3.2, 2.3)  .75 |  | 0.97  (-3.3, 5.2)  .65 |  | 0.08 |
| ^a^Means, SDs, difference in mean change are based on coefficients and combination of coefficients from mixed effects model following multiple imputation.  ^b^Positive mean difference in change in this table means that the *increase* in Wellbeing score was larger in EASE than PYA  ^c^ Cohen’s d effect size was calculated by dividing the predicted difference in mean change from the mixed effects model by the pooled baseline SD (11.6).  ^*^Model included fixed effects of arm, time and arm X time interaction, and random effects of pt_code, family ID, and Ease group.  T0=Baseline, T1=Endline | | | | | | | | | | | | |

| **Table 4.6bii Predicted means, mean changes, and effect sizes for Child Wellbeing outcome with imputed data among 13-14 year olds** | | | | | | | | | | | | |
| --- | --- | --- | --- | --- | --- | --- | --- | --- | --- | --- | --- | --- |
|  |  | **EASE (N=24)** | | |  | **PYA (N=41)** | | |  | **Between group treatment effect** | | |
|  |  | *Mean*  *(95% CI)^a^* |  | *Mean change from baseline*  *(95% CI)*  *p* |  | *Mean*  *(95% CI)^a^* |  | *Mean change from baseline*  *(95% CI)*  *p* |  | *Mean difference in change*  *(95% CI)^b^*  *p* |  | *Cohen’s d^c^* |
| T0 |  | 42.9  (38.6, 47.2) |  | - |  | 41.0  (37.7, 44.3) |  | - |  | - |  | - |
| T1 |  | 44.6  (40.2, 49.0) |  | 1.7  (-3.1, 6.5)  .49 |  | 41.1  (37.6, 44.6) |  | 0.07  (-3.7, 3.8)  .97 |  | 1.6  (-4.5, 7.7)  .61 |  | 0.16 |
| ^a^Means, SDs, difference in mean change are based on coefficients and combination of coefficients from mixed effects model following multiple imputation.  ^b^Positive mean difference in change in this table means that the *increase* in Wellbeing score was larger in EASE than PYA  ^c^ Cohen’s d effect size was calculated by dividing the predicted difference in mean change from the mixed effects model by the pooled baseline SD (10.2).  ^*^Model included fixed effects of arm, time and arm X time interaction, and random effects of pt_code, family ID, and Ease group.  T0=Baseline, T1=Endline | | | | | | | | | | | | |

### 4.6c Child Wellbeing: by PSC Severity

| **Table 4.6ci Predicted means, mean changes, and effect sizes for Child Wellbeing outcome with imputed data among lower 50% PSC Scores** | | | | | | | | | | | | |
| --- | --- | --- | --- | --- | --- | --- | --- | --- | --- | --- | --- | --- |
|  |  | **EASE (N=37)** | | |  | **PYA (N=59)** | | |  | **Between group treatment effect** | | |
|  |  | *Mean*  *(95% CI)^a^* |  | *Mean change from baseline*  *(95% CI)*  *p* |  | *Mean*  *(95% CI)^a^* |  | *Mean change from baseline*  *(95% CI)*  *p* |  | *Mean difference in change*  *(95% CI)^b^*  *p* |  | *Cohen’s d^c^* |
| T0 |  | 47.7  (44.4, 51.0) |  | - |  | 45.8  (43.2, 48.4) |  | - |  | - |  | - |
| T1 |  | 48.8  (45.4, 52.2) |  | 1.1  (-2.9, 5.1)  .59 |  | 45.2  (42.6, 47.9) |  | -0.57  (-3.7, 2.5)  .72 |  | 1.7  (-3.4, 6.8)  .52 |  | 0.16 |
| ^a^Means, SDs, difference in mean change are based on coefficients and combination of coefficients from mixed effects model following multiple imputation.  ^b^Positive mean difference in change in this table means that the *increase* in Wellbeing score was larger in EASE than PYA  ^c^ Cohen’s d effect size was calculated by dividing the predicted difference in mean change from the mixed effects model by the pooled baseline SD (10.4).  ^*^Model included fixed effects of arm, time and arm X time interaction, and random effects of pt_code, family ID, and Ease group.  T0=Baseline, T1=Endline | | | | | | | | | | | | |

| **Table 4.6cii Predicted means, mean changes, and effect sizes for Child Wellbeing outcome with imputed data among upper 50% PSC Scores** | | | | | | | | | | | | |
| --- | --- | --- | --- | --- | --- | --- | --- | --- | --- | --- | --- | --- |
|  |  | **EASE (N=43)** | | |  | **PYA (N=59)** | | |  | **Between group treatment effect** | | |
|  |  | *Mean*  *(95% CI)^a^* |  | *Mean change from baseline*  *(95% CI)*  *p* |  | *Mean*  *(95% CI)^a^* |  | *Mean change from baseline*  *(95% CI)*  *p* |  | *Mean difference in change*  *(95% CI)^b^*  *p* |  | *Cohen’s d^c^* |
| T0 |  | 42.1  (38.9, 45.2) |  | - |  | 42.8  (40.1, 45.5) |  | - |  | - |  | - |
| T1 |  | 42.7  (39.5, 45.9) |  | 0.67  (-3.0, 4.3)  .72 |  | 42.8  (40.0, 45.7) |  | 0.02  (-3.2, 3.2)  .99 |  | 0.64  (-4.2, 5.5)  .80 |  | 0.05 |
| ^a^Means, SDs, difference in mean change are based on coefficients and combination of coefficients from mixed effects model following multiple imputation.  ^b^Positive mean difference in change in this table means that the *increase* in Wellbeing score was larger in EASE than PYA  ^c^ Cohen’s d effect size was calculated by dividing the predicted difference in mean change from the mixed effects model by the pooled baseline SD (11.8).  ^*^Model included fixed effects of arm, time and arm X time interaction, and random effects of pt_code, family ID, and Ease group.  T0=Baseline, T1=Endline | | | | | | | | | | | | |

### 4.6d Child Wellbeing: by PSC Internalizing Severity

| **Table 4.6di Predicted means, mean changes, and effect sizes for Child Wellbeing outcome with imputed data among lower 50% PSC Internalizing Scores** | | | | | | | | | | | | |
| --- | --- | --- | --- | --- | --- | --- | --- | --- | --- | --- | --- | --- |
|  |  | **EASE (N=43)** | | |  | **PYA (N=66)** | | |  | **Between group treatment effect** | | |
|  |  | *Mean*  *(95% CI)^a^* |  | *Mean change from baseline*  *(95% CI)*  *p* |  | *Mean*  *(95% CI)^a^* |  | *Mean change from baseline*  *(95% CI)*  *p* |  | *Mean difference in change*  *(95% CI)^b^*  *p* |  | *Cohen’s d^c^* |
| T0 |  | 45.7  (42.6, 48.8) |  | - |  | 45.2  (42.7, 47.7) |  | - |  | - |  | - |
| T1 |  | 46.9  (43.6, 50.2) |  | 1.2  (-2.5, 4.9)  .52 |  | 47.1  (44.5, 49.7) |  | 1.9  (-1.0, 4.8)  .20 |  | -0.69  (-5.3, 4.0)  .77 |  | 0.06 |
| ^a^Means, SDs, difference in mean change are based on coefficients and combination of coefficients from mixed effects model following multiple imputation.  ^b^Negative mean difference in change in this table means that the *increase* in Wellbeing score was larger in PYA than EASE  ^c^ Cohen’s d effect size was calculated by dividing the predicted difference in mean change from the mixed effects model by the pooled baseline SD (11.3).  ^*^Model included fixed effects of arm, time and arm X time interaction, and random effects of pt_code, family ID, and Ease group.  T0=Baseline, T1=Endline | | | | | | | | | | | | |

| **Table 4.6dii Predicted means, mean changes, and effect sizes for Child Wellbeing outcome with imputed data among upper 50% PSC Internalizing Scores** | | | | | | | | | | | | |
| --- | --- | --- | --- | --- | --- | --- | --- | --- | --- | --- | --- | --- |
|  |  | **EASE (N=37)** | | |  | **PYA (N=52)** | | |  | **Between group treatment effect** | | |
|  |  | *Mean*  *(95% CI)^a^* |  | *Mean change from baseline*  *(95% CI)*  *p* |  | *Mean*  *(95% CI)^a^* |  | *Mean change from baseline*  *(95% CI)*  *p* |  | *Mean difference in change*  *(95% CI)^b^*  *p* |  | *Cohen’s d^c^* |
| T0 |  | 43.5  (40.1, 46.9) |  | - |  | 43.1  (40.3, 46.0) |  | - |  | - |  | - |
| T1 |  | 44.0  (40.6, 47.4) |  | 0.48  (-3.5, 4.4)  .81 |  | 40.1  (37.1, 43.1) |  | -3.0  (-6.4, 0.39)  .08 |  | 3.5  (-1.7, 8.7)  .19 |  | 0.31 |
| ^a^Means, SDs, difference in mean change are based on coefficients and combination of coefficients from mixed effects model following multiple imputation.  ^b^Positive mean difference in change in this table means that the *increase* in Wellbeing score was larger in EASE than PYA  ^c^ Cohen’s d effect size was calculated by dividing the predicted difference in mean change from the mixed effects model by the pooled baseline SD (11.3).  ^*^Model included fixed effects of arm, time and arm X time interaction, and random effects of pt_code, family ID, and Ease group.  T0=Baseline, T1=Endline | | | | | | | | | | | | |

### 4.6e Child Wellbeing: by PSC Externalizing Severity

| **Table 4.6ei Predicted means, mean changes, and effect sizes for Child Wellbeing outcome with imputed data among lower 50% PSC Externalizing Scores** | | | | | | | | | | | | |
| --- | --- | --- | --- | --- | --- | --- | --- | --- | --- | --- | --- | --- |
|  |  | **EASE (N=39)** | | |  | **PYA (N=56)** | | |  | **Between group treatment effect** | | |
|  |  | *Mean*  *(95% CI)^a^* |  | *Mean change from baseline*  *(95% CI)*  *p* |  | *Mean*  *(95% CI)^a^* |  | *Mean change from baseline*  *(95% CI)*  *p* |  | *Mean difference in change*  *(95% CI)^b^*  *p* |  | *Cohen’s d^c^* |
| T0 |  | 45.7  (42.3, 49.0) |  | - |  | 45.1  (42.2, 47.9) |  | - |  | - |  | - |
| T1 |  | 48.0  (44.5, 51.6) |  | 2.4  (-1.7, 6.5)  .26 |  | 44.2  (41.3, 47.0) |  | -0.91  (-4.2, 2.4)  .59 |  | 3.3  (-2.0, 8.5)  .22 |  | 0.31 |
| ^a^Means, SDs, difference in mean change are based on coefficients and combination of coefficients from mixed effects model following multiple imputation.  ^b^Positive mean difference in change in this table means that the *increase* in Wellbeing score was larger in EASE than PYA  ^c^ Cohen’s d effect size was calculated by dividing the predicted difference in mean change from the mixed effects model by the pooled baseline SD (10.8).  ^*^Model included fixed effects of arm, time and arm X time interaction, and random effects of pt_code, family ID, and Ease group.  T0=Baseline, T1=Endline | | | | | | | | | | | | |

| **Table 4.6eii Predicted means, mean changes, and effect sizes for Child Wellbeing outcome with imputed data among upper 50% PSC Externalizing Scores** | | | | | | | | | | | | |
| --- | --- | --- | --- | --- | --- | --- | --- | --- | --- | --- | --- | --- |
|  |  | **EASE (N=41)** | | |  | **PYA (N=62)** | | |  | **Between group treatment effect** | | |
|  |  | *Mean*  *(95% CI)^a^* |  | *Mean change from baseline*  *(95% CI)*  *p* |  | *Mean*  *(95% CI)^a^* |  | *Mean change from baseline*  *(95% CI)*  *p* |  | *Mean difference in change*  *(95% CI)^b^*  *p* |  | *Cohen’s d^c^* |
| T0 |  | 43.8  (40.6, 46.9) |  | - |  | 43.6  (41.0, 46.1) |  | - |  | - |  | - |
| T1 |  | 43.2  (40.0, 46.4) |  | -0.57  (-4.1, 3.0)  .76 |  | 43.9  (41.2, 46.6) |  | 0.30  (-2.6, 3.2)  .84 |  | -0.87  (-5.5, 3.8)  .72 |  | 0.07 |
| ^a^Means, SDs, difference in mean change are based on coefficients and combination of coefficients from mixed effects model following multiple imputation.  ^b^Negative mean difference in change in this table means that the *increase* in Wellbeing score was larger in PYA than EASE  ^c^ Cohen’s d effect size was calculated by dividing the predicted difference in mean change from the mixed effects model by the pooled baseline SD (11.8).  ^*^Model included fixed effects of arm, time and arm X time interaction, and random effects of pt_code, family ID, and Ease group.  T0=Baseline, T1=Endline | | | | | | | | | | | | |

### 4.6f Child Wellbeing: by PHQ Severity

| **Table 4.6fi Predicted means, mean changes, and effect sizes for Child Wellbeing outcome with imputed data among lower 50% PHQ Scores** | | | | | | | | | | | | |
| --- | --- | --- | --- | --- | --- | --- | --- | --- | --- | --- | --- | --- |
|  |  | **EASE (N=40)** | | |  | **PYA (N=57)** | | |  | **Between group treatment effect** | | |
|  |  | *Mean*  *(95% CI)^a^* |  | *Mean change from baseline*  *(95% CI)*  *p* |  | *Mean*  *(95% CI)^a^* |  | *Mean change from baseline*  *(95% CI)*  *p* |  | *Mean difference in change*  *(95% CI)^b^*  *p* |  | *Cohen’s d^c^* |
| T0 |  | 49.1  (46.1, 52.2) |  | - |  | 46.4  (43.8, 48.9) |  | - |  | - |  | - |
| T1 |  | 47.6  (44.4, 50.9) |  | -1.5  (-5.2, 2.2)  .43 |  | 46.1  (43.4, 48.8) |  | -0.26  (-3.4, 2.8)  .87 |  | -1.2  (-6.0, 3.5)  .61 |  | 0.11 |
| ^a^Means, SDs, difference in mean change are based on coefficients and combination of coefficients from mixed effects model following multiple imputation.  ^b^Negative mean difference in change in this table means that the *decrease* in Wellbeing score was larger in EASE than PYA  ^c^ Cohen’s d effect size was calculated by dividing the predicted difference in mean change from the mixed effects model by the pooled baseline SD (10.5).  ^*^Model included fixed effects of arm, time and arm X time interaction, and random effects of pt_code, family ID, and Ease group.  T0=Baseline, T1=Endline | | | | | | | | | | | | |

| **Table 4.6fii Predicted means, mean changes, and effect sizes for Child Wellbeing outcome with imputed data among upper 50% PHQ Scores** | | | | | | | | | | | | |
| --- | --- | --- | --- | --- | --- | --- | --- | --- | --- | --- | --- | --- |
|  |  | **EASE (N=40)** | | |  | **PYA (N=61)** | | |  | **Between group treatment effect** | | |
|  |  | *Mean*  *(95% CI)^a^* |  | *Mean change from baseline*  *(95% CI)*  *p* |  | *Mean*  *(95% CI)^a^* |  | *Mean change from baseline*  *(95% CI)*  *p* |  | *Mean difference in change*  *(95% CI)^b^*  *p* |  | *Cohen’s d^c^* |
| T0 |  | 40.2  (36.9, 43.5) |  | - |  | 42.4  (39.7, 45.0) |  | - |  | - |  | - |
| T1 |  | 43.5  (40.1, 46.8) |  | 3.2  (-0.63, 7.1)  .10 |  | 42.1  (39.3, 44.8) |  | -0.28  (-3.4, 2.9)  .86 |  | 3.5  (-1.5, 8.5)  .17 |  | 0.31 |
| ^a^Means, SDs, difference in mean change are based on coefficients and combination of coefficients from mixed effects model following multiple imputation.  ^b^Positive mean difference in change in this table means that the *increase* in Wellbeing score was larger in EASE than PYA  ^c^ Cohen’s d effect size was calculated by dividing the predicted difference in mean change from the mixed effects model by the pooled baseline SD (11.3).  ^*^Model included fixed effects of arm, time and arm X time interaction, and random effects of pt_code, family ID, and Ease group.  T0=Baseline, T1=Endline | | | | | | | | | | | | |

### 4.6g Child Wellbeing: by Child Wellbeing Severity

| **Table 4.6gi Predicted means, mean changes, and effect sizes for Child Wellbeing outcome with imputed data among lower 50% Child Wellbeing Scores** | | | | | | | | | | | | |
| --- | --- | --- | --- | --- | --- | --- | --- | --- | --- | --- | --- | --- |
|  |  | **EASE (N=36)** | | |  | **PYA (N=58)** | | |  | **Between group treatment effect** | | |
|  |  | *Mean*  *(95% CI)^a^* |  | *Mean change from baseline*  *(95% CI)*  *p* |  | *Mean*  *(95% CI)^a^* |  | *Mean change from baseline*  *(95% CI)*  *p* |  | *Mean difference in change*  *(95% CI)^b^*  *p* |  | *Cohen’s d^c^* |
| T0 |  | 34.5  (31.5, 37.6) |  | - |  | 35.4  (33.0, 37.8) |  | - |  | - |  | - |
| T1 |  | 40.0  (36.8, 43.1) |  | 5.5  (1.8, 9.1)  **<.01** |  | 40.5  (37.9, 43.0) |  | 5.1  (2.2, 8.0)  **<.0001** |  | 0.38  (-4.2, 5.0)  .87 |  | 0.06 |
| ^a^Means, SDs, difference in mean change are based on coefficients and combination of coefficients from mixed effects model following multiple imputation.  ^b^Positive mean difference in change in this table means that the *increase* in Wellbeing score was larger in EASE than PYA  ^c^ Cohen’s d effect size was calculated by dividing the predicted difference in mean change from the mixed effects model by the pooled baseline SD (6.9).  ^*^Model included fixed effects of arm, time and arm X time interaction, and random effects of pt_code, family ID, and Ease group.  T0=Baseline, T1=Endline | | | | | | | | | | | | |

| **Table 4.6gii Predicted means, mean changes, and effect sizes for Child Wellbeing outcome with imputed data among upper 50% Child Wellbeing Scores** | | | | | | | | | | | | |
| --- | --- | --- | --- | --- | --- | --- | --- | --- | --- | --- | --- | --- |
|  |  | **EASE (N=44)** | | |  | **PYA (N=60)** | | |  | **Between group treatment effect** | | |
|  |  | *Mean*  *(95% CI)^a^* |  | *Mean change from baseline*  *(95% CI)*  *p* |  | *Mean*  *(95% CI)^a^* |  | *Mean change from baseline*  *(95% CI)*  *p* |  | *Mean difference in change*  *(95% CI)^b^*  *p* |  | *Cohen’s d^c^* |
| T0 |  | 53.0  (50.3, 55.7) |  | - |  | 53.0  (50.7, 55.3) |  | - |  | - |  | - |
| T1 |  | 50.1  (47.4, 52.9) |  | -2.9  (-6.4, 0.61)  .11 |  | 47.5  (45.1, 49.9) |  | -5.4  (-8.5, -2.4)  <.0001 |  | 2.6  (-2.1, 7.2)  .28 |  | 0.37 |
| ^a^Means, SDs, difference in mean change are based on coefficients and combination of coefficients from mixed effects model following multiple imputation.  ^b^Positive mean difference in change in this table means that the *decrease* in Wellbeing score was smaller in EASE than PYA  ^c^ Cohen’s d effect size was calculated by dividing the predicted difference in mean change from the mixed effects model by the pooled baseline SD (7.0).  ^*^Model included fixed effects of arm, time and arm X time interaction, and random effects of pt_code, family ID, and Ease group.  T0=Baseline, T1=Endline | | | | | | | | | | | | |

### 4.6h Child Wellbeing: by Caregiver PSC Severity

| **Table 4.6hi Predicted means, mean changes, and effect sizes for Child Wellbeing outcome with imputed data among lower 50% Caregiver PSC Scores** | | | | | | | | | | | | |
| --- | --- | --- | --- | --- | --- | --- | --- | --- | --- | --- | --- | --- |
|  |  | **EASE (N=36)** | | |  | **PYA (N=59)** | | |  | **Between group treatment effect** | | |
|  |  | *Mean*  *(95% CI)^a^* |  | *Mean change from baseline*  *(95% CI)*  *p* |  | *Mean*  *(95% CI)^a^* |  | *Mean change from baseline*  *(95% CI)*  *p* |  | *Mean difference in change*  *(95% CI)^b^*  *p* |  | *Cohen’s d^c^* |
| T0 |  | 45.9  (42.5, 49.4) |  | - |  | 46.2  (43.5, 48.9) |  | - |  | - |  | - |
| T1 |  | 45.2  (42.4, 48.1) |  | 2.3  (-1.7, 6.4)  .26 |  | 48.3  (44.7, 51.9) |  | -0.98  (-4.2, 2.2)  .55 |  | 3.3  (-1.8, 8.5)  .21 |  | 0.31 |
| ^a^Means, SDs, difference in mean change are based on coefficients and combination of coefficients from mixed effects model following multiple imputation.  ^b^Positive mean difference in change in this table means that the *increase* in Wellbeing score was larger in EASE than PYA  ^c^ Cohen’s d effect size was calculated by dividing the predicted difference in mean change from the mixed effects model by the pooled baseline SD (10.7).  ^*^Model included fixed effects of arm, time and arm X time interaction, and random effects of pt_code, family ID, and Ease group.  T0=Baseline, T1=Endline | | | | | | | | | | | | |

| **Table 4.6hii Predicted means, mean changes, and effect sizes for Child Wellbeing outcome with imputed data among upper 50% Caregiver PSC Scores** | | | | | | | | | | | | |
| --- | --- | --- | --- | --- | --- | --- | --- | --- | --- | --- | --- | --- |
|  |  | **EASE (N=44)** | | |  | **PYA (N=59)** | | |  | **Between group treatment effect** | | |
|  |  | *Mean*  *(95% CI)^a^* |  | *Mean change from baseline*  *(95% CI)*  *p* |  | *Mean*  *(95% CI)^a^* |  | *Mean change from baseline*  *(95% CI)*  *p* |  | *Mean difference in change*  *(95% CI)^b^*  *p* |  | *Cohen’s d^c^* |
| T0 |  | 43.7  (40.6, 46.7) |  | - |  | 42.5  (39.8, 45.1) |  | - |  | - |  | - |
| T1 |  | 43.3  (40.2, 46.4) |  | -0.34  (-3.9, 3.2)  .85 |  | 42.9  (40.1, 45.6) |  | 0.43  (-2.7, 3.6)  .79 |  | -0.77  (-5.5, 4.0)  .75 |  | 0.07 |
| ^a^Means, SDs, difference in mean change are based on coefficients and combination of coefficients from mixed effects model following multiple imputation.  ^b^Negative mean difference in change in this table means that the *increase* in PSC score was larger in PYA than EASE  ^c^ Cohen’s d effect size was calculated by dividing the predicted difference in mean change from the mixed effects model by the pooled baseline SD (11.7).  ^*^Model included fixed effects of arm, time and arm X time interaction, and random effects of pt_code, family ID, and Ease group.  T0=Baseline, T1=Endline | | | | | | | | | | | | |

### 4.6i Child Wellbeing: by Caregiver K6 Severity

| **Table 4.6ii Predicted means, mean changes, and effect sizes for Child Wellbeing outcome with imputed data among lower 50% Caregiver K6 Scores** | | | | | | | | | | | | |
| --- | --- | --- | --- | --- | --- | --- | --- | --- | --- | --- | --- | --- |
|  |  | **EASE (N=)** | | |  | **PYA (N=)** | | |  | **Between group treatment effect** | | |
|  |  | *Mean*  *(95% CI)^a^* |  | *Mean change from baseline*  *(95% CI)*  *p* |  | *Mean*  *(95% CI)^a^* |  | *Mean change from baseline*  *(95% CI)*  *p* |  | *Mean difference in change*  *(95% CI)^b^*  *p* |  | *Cohen’s d^c^* |
| T0 |  | 46.5  (43.3, 50.0) |  | - |  | 45.3  (42.2, 48.3) |  | - |  | - |  | - |
| T1 |  | 45.8  (42.6, 49.0) |  | -0.71  (-4.3, 2.9)  .70 |  | 44.9  (41.7, 48.1 |  | -0.35  (-4.0, 3.3)  .85 |  | -0.36  (-5.4, 4.7)  .89 |  | 0.03 |
| ^a^Means, SDs, difference in mean change are based on coefficients and combination of coefficients from mixed effects model following multiple imputation.  ^b^Negative mean difference in change in this table means that the *decrease* in Wellbeing score was larger in EASE than PYA  ^c^ Cohen’s d effect size was calculated by dividing the predicted difference in mean change from the mixed effects model by the pooled baseline SD (11.2).  ^*^Model included fixed effects of arm, time and arm X time interaction, and random effects of pt_code, family ID, and Ease group.  T0=Baseline, T1=Endline | | | | | | | | | | | | |

| **Table 4.6iii Predicted means, mean changes, and effect sizes for Child Wellbeing outcome with imputed data among upper 50% Caregiver K6 Scores** | | | | | | | | | | | | |
| --- | --- | --- | --- | --- | --- | --- | --- | --- | --- | --- | --- | --- |
|  |  | **EASE (N=45)** | | |  | **PYA (N=48)** | | |  | **Between group treatment effect** | | |
|  |  | *Mean*  *(95% CI)^a^* |  | *Mean change from baseline*  *(95% CI)*  *p* |  | *Mean*  *(95% CI)^a^* |  | *Mean change from baseline*  *(95% CI)*  *p* |  | *Mean difference in change*  *(95% CI)^b^*  *p* |  | *Cohen’s d^c^* |
| T0 |  | 42.3  (38.9, 45.8) |  | - |  | 43.7  (41.3, 46.1) |  | - |  | - |  | - |
| T1 |  | 45.2  (41.6, 48.9) |  | 2.9  (-1.1, 7.0)  .16 |  | 43.5  (41.0, 45.9) |  | -0.22  (-3.0, 2.6)  .88 |  | 3.1  (-1.8, 8.1)  .21 |  | 0.27 |
| ^a^Means, SDs, difference in mean change are based on coefficients and combination of coefficients from mixed effects model following multiple imputation.  ^b^Positive mean difference in change in this table means that the *increase* in Wellbeing score was larger in EASE than PYA  ^c^ Cohen’s d effect size was calculated by dividing the predicted difference in mean change from the mixed effects model by the pooled baseline SD (11.3).  ^*^Model included fixed effects of arm, time and arm X time interaction, and random effects of pt_code, family ID, and Ease group.  T0=Baseline, T1=Endline | | | | | | | | | | | | |

### 4.6j Child Wellbeing: by Treatment Completer Status

| **Table 4.6ji Predicted means, mean changes, and effect sizes for Child Wellbeing outcome with imputed data among treatment completers** | | | | | | | | | | | | |
| --- | --- | --- | --- | --- | --- | --- | --- | --- | --- | --- | --- | --- |
|  |  | **EASE (N=56)** | | |  | **PYA (N=117)** | | |  | **Between group treatment effect** | | |
|  |  | *Mean*  *(95% CI)^a^* |  | *Mean change from baseline*  *(95% CI)*  *p* |  | *Mean*  *(95% CI)^a^* |  | *Mean change from baseline*  *(95% CI)*  *p* |  | *Mean difference in change*  *(95% CI)^b^*  *p* |  | *Cohen’s d^c^* |
| T0 |  | 44.5  (41.8, 47.2) |  | - |  | 44.3  (42.4, 46.2) |  | - |  | - |  | - |
| T1 |  | 46.4  (43.7, 49.2) |  | 1.9  (-1.2, 5.1)  .23 |  | 44.0  (42.0, 45.9) |  | -0.32  (-2.5, 1.9)  .78 |  | 2.2  (-1.6, 6.1)  .25 |  | 0.20 |
| ^a^Means, SDs, difference in mean change are based on coefficients and combination of coefficients from mixed effects model following multiple imputation.  ^b^Positive mean difference in change in this table means that the *decrease* in Wellbeing score was larger in EASE than PYA  ^c^ Cohen’s d effect size was calculated by dividing the predicted difference in mean change from the mixed effects model by the pooled baseline SD (11.2).  ^*^Model included fixed effects of arm, time and arm X time interaction, and random effects of pt_code, family ID, and Ease group.  T0=Baseline, T1=Endline | | | | | | | | | | | | |

## Caregiver report of Child Psychological Symptoms (PSC)

### 4.10a Caregiver PSC: by Gender

| **Table 4.10ai Predicted means, mean changes, and effect sizes for Caregiver PSC outcome with imputed data among MALES (child)** | | | | | | | | | | | | |
| --- | --- | --- | --- | --- | --- | --- | --- | --- | --- | --- | --- | --- |
|  |  | **EASE (N=43)** | | |  | **PYA (N=58)** | | |  | **Between group treatment effect** | | |
|  |  | *Mean*  *(95% CI)^a^* |  | *Mean change from baseline*  *(95% CI)*  *p* |  | *Mean*  *(95% CI)^a^* |  | *Mean change from baseline*  *(95% CI)*  *p* |  | *Mean difference in change*  *(95% CI)^b^*  *p* |  | *Cohen’s d^c^* |
| T0 |  | 31.4  (28.1, 34.7) |  | - |  | 26.3  (23.1, 29.5) |  | - |  | - |  | - |
| T1 |  | 29.0  (25.6, 32.3) |  | -2.5  (-5.3, 0.32)  .08 |  | 28.7  (25.4, 32.1) |  | 2.4  (-0.07, 4.9)  .06 |  | -4.9  (-8.7, -1.2)  **.01** |  | 0.47 |
| ^a^Means, SDs, difference in mean change are based on coefficients and combination of coefficients from mixed effects model following multiple imputation.  ^b^Negative mean difference in change in this table means that the *decrease* in PSC score was larger in EASE than PYA  ^c^ Cohen’s d effect size was calculated by dividing the predicted difference in mean change from the mixed effects model by the pooled baseline SD (10.4).  ^*^Model included fixed effects of arm, time and arm X time interaction, and random effects of pt_code, family ID, and Ease group.  T0=Baseline, T1=Endline | | | | | | | | | | | | |

| **Table 4.10aii Predicted means, mean changes, and effect sizes for Caregiver PSC outcome with imputed data among FEMALES (child)** | | | | | | | | | | | | |
| --- | --- | --- | --- | --- | --- | --- | --- | --- | --- | --- | --- | --- |
|  |  | **EASE (N=37)** | | |  | **PYA (N=60)** | | |  | **Between group treatment effect** | | |
|  |  | *Mean*  *(95% CI)^a^* |  | *Mean change from baseline*  *(95% CI)*  *p* |  | *Mean*  *(95% CI)^a^* |  | *Mean change from baseline*  *(95% CI)*  *p* |  | *Mean difference in change*  *(95% CI)^b^*  *p* |  | *Cohen’s d^c^* |
| T0 |  | 25.3  (22.2, 28.4) |  | - |  | 28.7  (26.1, 31.2 |  | - |  | - |  | - |
| T1 |  | 25.3  (22.1, 28.5) |  | 0.04  (-3.2, 3.3)  .98 |  | 26.4  (23.6, 29.2) |  | -2.2  (-5.0, 0.48)  .11 |  | 2.3  (-2.0, 6.6)  .29 |  | 0.23 |
| ^a^Means, SDs, difference in mean change are based on coefficients and combination of coefficients from mixed effects model following multiple imputation.  ^b^Positive mean difference in change in this table means that the *decrease* in PSC score was larger in PYA than EASE  ^c^ Cohen’s d effect size was calculated by dividing the predicted difference in mean change from the mixed effects model by the pooled baseline SD (9.8).  ^*^Model included fixed effects of arm, time and arm X time interaction, and random effects of pt_code, family ID, and Ease group.  T0=Baseline, T1=Endline | | | | | | | | | | | | |

### 4.10b Caregiver PSC: by Age

| **Table 4.10bi Predicted means, mean changes, and effect sizes for Caregiver PSC outcome with imputed data among 10-12 year olds (child)** | | | | | | | | | | | | |
| --- | --- | --- | --- | --- | --- | --- | --- | --- | --- | --- | --- | --- |
|  |  | **EASE (N=56)** | | |  | **PYA (N=77)** | | |  | **Between group treatment effect** | | |
|  |  | *Mean*  *(95% CI)^a^* |  | *Mean change from baseline*  *(95% CI)*  *p* |  | *Mean*  *(95% CI)^a^* |  | *Mean change from baseline*  *(95% CI)*  *p* |  | *Mean difference in change*  *(95% CI)^b^*  *p* |  | *Cohen’s d^c^* |
| T0 |  | 28.5  (25.1, 31.9) |  | - |  | 27.1  (20.1, 34.2) |  | - |  | - |  | - |
| T1 |  | 27.0  (23.5, 30.4) |  | -1.6  (-4.2, 1.1)  .25 |  | 28.2  (21.2, 35.3) |  | 1.1  (-1.2, 3.4)  .35 |  | -2.7  (-6.2, 0.88)  .14 |  | 0.28 |
| ^a^Means, SDs, difference in mean change are based on coefficients and combination of coefficients from mixed effects model following multiple imputation.  ^b^Negative mean difference in change in this table means that the *decrease* in PSC score was larger in EASE than PYA  ^c^ Cohen’s d effect size was calculated by dividing the predicted difference in mean change from the mixed effects model by the pooled baseline SD (9.8).  ^*^Model included fixed effects of arm, time and arm X time interaction, and random effects of pt_code, family ID, and Ease group.  T0=Baseline, T1=Endline | | | | | | | | | | | | |

| **Table 4.10bii Predicted means, mean changes, and effect sizes for Caregiver PSC outcome with imputed data among 13-14 year olds (child)** | | | | | | | | | | | | |
| --- | --- | --- | --- | --- | --- | --- | --- | --- | --- | --- | --- | --- |
|  |  | **EASE (N=24)** | | |  | **PYA (N=41)** | | |  | **Between group treatment effect** | | |
|  |  | *Mean*  *(95% CI)^a^* |  | *Mean change from baseline*  *(95% CI)*  *p* |  | *Mean*  *(95% CI)^a^* |  | *Mean change from baseline*  *(95% CI)*  *p* |  | *Mean difference in change*  *(95% CI)^b^*  *p* |  | *Cohen’s d^c^* |
| T0 |  | 27.9  (23.8, 32.1) |  | - |  | 28.0  (24.8, 31.2) |  | - |  | - |  | - |
| T1 |  | 27.2  (22.9, 31.5) |  | -0.74  (-4.5, 3.0)  .70 |  | 26.1  (22.7, 29.4) |  | -1.9  (-4.9, 1.0)  .20 |  | 1.2  (-3.7, 6.0)  .64 |  | 0.11 |
| ^a^Means, SDs, difference in mean change are based on coefficients and combination of coefficients from mixed effects model following multiple imputation.  ^b^Positive mean difference in change in this table means that the *decrease* in PSC score was larger in PYA than EASE  ^c^ Cohen’s d effect size was calculated by dividing the predicted difference in mean change from the mixed effects model by the pooled baseline SD (10.8).  ^*^Model included fixed effects of arm, time and arm X time interaction, and random effects of pt_code, family ID, and Ease group.  T0=Baseline, T1=Endline | | | | | | | | | | | | |

### 4.10c Caregiver PSC: by PSC Severity

| **Table 4.10ci Predicted means, mean changes, and effect sizes for Caregiver PSC outcome with imputed data among lower 50% PSC Scores (child)** | | | | | | | | | | | | |
| --- | --- | --- | --- | --- | --- | --- | --- | --- | --- | --- | --- | --- |
|  |  | **EASE (N=37)** | | |  | **PYA (N=59)** | | |  | **Between group treatment effect** | | |
|  |  | *Mean*  *(95% CI)^a^* |  | *Mean change from baseline*  *(95% CI)*  *p* |  | *Mean*  *(95% CI)^a^* |  | *Mean change from baseline*  *(95% CI)*  *p* |  | *Mean difference in change*  *(95% CI)^b^*  *p* |  | *Cohen’s d^c^* |
| T0 |  | 23.7  (20.6, 26.7) |  | - |  | 24.8  (22.3, 27.3) |  | - |  | - |  | - |
| T1 |  | 24.5  (21.9, 27.2) |  | -0.08  (-3.1, 2.9)  .96 |  | 23.6  (20.4, 26.7) |  | -0.29  (-2.7, 2.2)  .82 |  | 0.20  (-3.7, 4.1)  .92 |  | 0.02 |
| ^a^Means, SDs, difference in mean change are based on coefficients and combination of coefficients from mixed effects model following multiple imputation.  ^b^Positive mean difference in change in this table means that the *decrease* in PSC score was larger in PYA than EASE  ^c^ Cohen’s d effect size was calculated by dividing the predicted difference in mean change from the mixed effects model by the pooled baseline SD (8.9).  ^*^Model included fixed effects of arm, time and arm X time interaction, and random effects of pt_code, family ID, and Ease group.  T0=Baseline, T1=Endline | | | | | | | | | | | | |

| **Table 4.10cii Predicted means, mean changes, and effect sizes for Caregiver PSC outcome with imputed data among upper 50% PSC Scores (child)** | | | | | | | | | | | | |
| --- | --- | --- | --- | --- | --- | --- | --- | --- | --- | --- | --- | --- |
|  |  | **EASE (N=43)** | | |  | **PYA (N=59)** | | |  | **Between group treatment effect** | | |
|  |  | *Mean*  *(95% CI)^a^* |  | *Mean change from baseline*  *(95% CI)*  *p* |  | *Mean*  *(95% CI)^a^* |  | *Mean change from baseline*  *(95% CI)*  *p* |  | *Mean difference in change*  *(95% CI)^b^*  *p* |  | *Cohen’s d^c^* |
| T0 |  | 32.7  (29.6, 35.8) |  | - |  | 30.6  (27.8, 33.4) |  | - |  | - |  | - |
| T1 |  | 30.3  (27.2, 33.5) |  | -2.4  (-5.4, 0.68)  .13 |  | 31.0  (28.0, 33.9) |  | 0.39  (-2.3, 3.1)  .78 |  | -2.8  (-6.9, 1.3)  .19 |  | 0.28 |
| ^a^Means, SDs, difference in mean change are based on coefficients and combination of coefficients from mixed effects model following multiple imputation.  ^b^Negative mean difference in change in this table means that the *decrease* in PSC score was larger in EASE than PYA  ^c^ Cohen’s d effect size was calculated by dividing the predicted difference in mean change from the mixed effects model by the pooled baseline SD (10.0).  ^*^Model included fixed effects of arm, time and arm X time interaction, and random effects of pt_code, family ID, and Ease group.  T0=Baseline, T1=Endline | | | | | | | | | | | | |

### 4.10d Caregiver PSC: by PSC Internalizing Severity

| **Table 4.10di Predicted means, mean changes, and effect sizes for Caregiver PSC outcome with imputed data among lower 50% PSC Internalizing Scores (Child)** | | | | | | | | | | | | |
| --- | --- | --- | --- | --- | --- | --- | --- | --- | --- | --- | --- | --- |
|  |  | **EASE (N=43)** | | |  | **PYA (N=66)** | | |  | **Between group treatment effect** | | |
|  |  | *Mean*  *(95% CI)^a^* |  | *Mean change from baseline*  *(95% CI)*  *p* |  | *Mean*  *(95% CI)^a^* |  | *Mean change from baseline*  *(95% CI)*  *p* |  | *Mean difference in change*  *(95% CI)^b^*  *p* |  | *Cohen’s d^c^* |
| T0 |  | 28.9  (25.9, 31.9) |  | - |  | 26.0  (23.4, 28.6) |  | - |  | - |  | - |
| T1 |  | 26.1  (23.0, 29.2) |  | -2.8  (-5.7, 0.09)  .06 |  | 26.8  (24.1, 29.5) |  | 0.84  (-1.6, 3.2)  .50 |  | -3.6  (-7.4, 0.10)  .06 |  | 0.36 |
| ^a^Means, SDs, difference in mean change are based on coefficients and combination of coefficients from mixed effects model following multiple imputation.  ^b^Negative mean difference in change in this table means that the *decrease* in PSC score was larger in EASE than PYA  ^c^ Cohen’s d effect size was calculated by dividing the predicted difference in mean change from the mixed effects model by the pooled baseline SD (9.9).  ^*^Model included fixed effects of arm, time and arm X time interaction, and random effects of pt_code, family ID, and Ease group.  T0=Baseline, T1=Endline | | | | | | | | | | | | |

| **Table 4.10dii Predicted means, mean changes, and effect sizes for Caregiver PSC outcome with imputed data among upper 50% PSC Internalizing Scores (Child)** | | | | | | | | | | | | |
| --- | --- | --- | --- | --- | --- | --- | --- | --- | --- | --- | --- | --- |
|  |  | **EASE (N=37)** | | |  | **PYA (N=52)** | | |  | **Between group treatment effect** | | |
|  |  | *Mean*  *(95% CI)^a^* |  | *Mean change from baseline*  *(95% CI)*  *p* |  | *Mean*  *(95% CI)^a^* |  | *Mean change from baseline*  *(95% CI)*  *p* |  | *Mean difference in change*  *(95% CI)^b^*  *p* |  | *Cohen’s d^c^* |
| T0 |  | 27.6  (23.7, 31.4) |  | - |  | 29.7  (23.0, 36.4) |  | - |  | - |  | - |
| T1 |  | 28.0  (24.1, 31.9) |  | 0.41  (-2.8, 3.7)  .80 |  | 28.7  (21.9, 35.5) |  | -0.95  (-3.9, 2.0)  .52 |  | 1.4  (-3.0, 5.7)  .54 |  | 0.14 |
| ^a^Means, SDs, difference in mean change are based on coefficients and combination of coefficients from mixed effects model following multiple imputation.  ^b^Positive mean difference in change in this table means that the *decrease* in PSC score was larger in PYA than EASE  ^c^ Cohen’s d effect size was calculated by dividing the predicted difference in mean change from the mixed effects model by the pooled baseline SD (10.3).  ^*^Model included fixed effects of arm, time and arm X time interaction, and random effects of pt_code, family ID, and Ease group.  T0=Baseline, T1=Endline | | | | | | | | | | | | |

### 4.10e Caregiver PSC: by PSC Externalizing Severity

| **Table 4.10ei Predicted means, mean changes, and effect sizes for Caregiver PSC outcome with imputed data among lower 50% PSC Externalizing Scores (Child)** | | | | | | | | | | | | |
| --- | --- | --- | --- | --- | --- | --- | --- | --- | --- | --- | --- | --- |
|  |  | **EASE (N=39)** | | |  | **PYA (N=56)** | | |  | **Between group treatment effect** | | |
|  |  | *Mean*  *(95% CI)^a^* |  | *Mean change from baseline*  *(95% CI)*  *p* |  | *Mean*  *(95% CI)^a^* |  | *Mean change from baseline*  *(95% CI)*  *p* |  | *Mean difference in change*  *(95% CI)^b^*  *p* |  | *Cohen’s d^c^* |
| T0 |  | 25.0  (21.8, 28.1) |  | - |  | 27.1  (24.4, 29.9) |  | - |  | - |  | - |
| T1 |  | 24.8  (21.6, 28.1) |  | -0.15  (-3.2, 2.9)  .92 |  | 26.8  (24.0, 30.0) |  | -0.27  (-2.9, 2.3)  .84 |  | 0.12  (-4.0, 4.2)  .95 |  | 0.01 |
| ^a^Means, SDs, difference in mean change are based on coefficients and combination of coefficients from mixed effects model following multiple imputation.  ^b^Positive mean difference in change in this table means that the *decrease* in PSC score was larger in PYA than EASE  ^c^ Cohen’s d effect size was calculated by dividing the predicted difference in mean change from the mixed effects model by the pooled baseline SD (9.7).  ^*^Model included fixed effects of arm, time and arm X time interaction, and random effects of pt_code, family ID, and Ease group.  T0=Baseline, T1=Endline | | | | | | | | | | | | |

| **Table 4.10eii Predicted means, mean changes, and effect sizes for Caregiver PSC outcome with imputed data among upper 50% PSC Externalizing Scores (Child)** | | | | | | | | | | | | |
| --- | --- | --- | --- | --- | --- | --- | --- | --- | --- | --- | --- | --- |
|  |  | **EASE (N=41)** | | |  | **PYA (N=62)** | | |  | **Between group treatment effect** | | |
|  |  | *Mean*  *(95% CI)^a^* |  | *Mean change from baseline*  *(95% CI)*  *p* |  | *Mean*  *(95% CI)^a^* |  | *Mean change from baseline*  *(95% CI)*  *p* |  | *Mean difference in change*  *(95% CI)^b^*  *p* |  | *Cohen’s d^c^* |
| T0 |  | 31.9  (28.7, 35.1) |  | - |  | 27.9  (25.2, 30.7) |  | - |  | - |  | - |
| T1 |  | 29.5  (26.2, 32.7) |  | -2.4  (-5.5, 0.60)  .12 |  | 28.3  (25.3, 31.4) |  | 0.40  (-2.4, 3.2)  .78 |  | -2.8  (-6.9, 1.2)  .17 |  | 0.27 |
| ^a^Means, SDs, difference in mean change are based on coefficients and combination of coefficients from mixed effects model following multiple imputation.  ^b^Negative mean difference in change in this table means that the *decrease* in PSC score was larger in EASE than PYA  ^c^ Cohen’s d effect size was calculated by dividing the predicted difference in mean change from the mixed effects model by the pooled baseline SD (10.2).  ^*^Model included fixed effects of arm, time and arm X time interaction, and random effects of pt_code, family ID, and Ease group.  T0=Baseline, T1=Endline | | | | | | | | | | | | |

### 4.10f Caregiver PSC: by PHQ Severity

| **Table 4.10fi Predicted means, mean changes, and effect sizes for Caregiver PSC outcome with imputed data among lower 50% PHQ Scores (Child)** | | | | | | | | | | | | |
| --- | --- | --- | --- | --- | --- | --- | --- | --- | --- | --- | --- | --- |
|  |  | **EASE (N=40)** | | |  | **PYA (N=57)** | | |  | **Between group treatment effect** | | |
|  |  | *Mean*  *(95% CI)^a^* |  | *Mean change from baseline*  *(95% CI)*  *p* |  | *Mean*  *(95% CI)^a^* |  | *Mean change from baseline*  *(95% CI)*  *p* |  | *Mean difference in change*  *(95% CI)^b^*  *p* |  | *Cohen’s d^c^* |
| T0 |  | 26.3  (23.3, 29.3) |  | - |  | 25.0  (22.4, 27.6) |  | - |  | - |  | - |
| T1 |  | 24.4  (21.3, 27.5) |  | -1.9  (-4.8, 1.1)  .22 |  | 26.7  (23.9, 29.5) |  | 1.7  (-0.94, 4.3)  .21 |  | -3.6  (-7.6, 0.44)  .08 |  | 0.38 |
| ^a^Means, SDs, difference in mean change are based on coefficients and combination of coefficients from mixed effects model following multiple imputation.  ^b^Negative mean difference in change in this table means that the *decrease* in PSC score was larger in EASE than PYA  ^c^ Cohen’s d effect size was calculated by dividing the predicted difference in mean change from the mixed effects model by the pooled baseline SD (9.5).  ^*^Model included fixed effects of arm, time and arm X time interaction, and random effects of pt_code, family ID, and Ease group.  T0=Baseline, T1=Endline | | | | | | | | | | | | |

| **Table 4.10fii Predicted means, mean changes, and effect sizes for Caregiver PSC outcome with imputed data among upper 50% PHQ Scores (Child)** | | | | | | | | | | | | |
| --- | --- | --- | --- | --- | --- | --- | --- | --- | --- | --- | --- | --- |
|  |  | **EASE (N=40)** | | |  | **PYA (N=61)** | | |  | **Between group treatment effect** | | |
|  |  | *Mean*  *(95% CI)^a^* |  | *Mean change from baseline*  *(95% CI)*  *p* |  | *Mean*  *(95% CI)^a^* |  | *Mean change from baseline*  *(95% CI)*  *p* |  | *Mean difference in change*  *(95% CI)^b^*  *p* |  | *Cohen’s d^c^* |
| T0 |  | 30.8  (27.5, 34.0) |  | - |  | 30.3  (27.5, 33.1) |  | - |  | - |  | - |
| T1 |  | 30.0  (26.7, 33.3) |  | -0.75  (-2.9, 2.4)  .64 |  | 28.9  (25.9, 31.8) |  | -1.5  (-4.2, 1.2)  .28 |  | 0.73  (-3.4, 4.9)  .73 |  | 0.07 |
| ^a^Means, SDs, difference in mean change are based on coefficients and combination of coefficients from mixed effects model following multiple imputation.  ^b^Positive mean difference in change in this table means that the *decrease* in PSC score was larger in PYA than EASE  ^c^ Cohen’s d effect size was calculated by dividing the predicted difference in mean change from the mixed effects model by the pooled baseline SD (10.2).  ^*^Model included fixed effects of arm, time and arm X time interaction, and random effects of pt_code, family ID, and Ease group.  T0=Baseline, T1=Endline | | | | | | | | | | | | |

### 4.10g Caregiver PSC: by Child Wellbeing Severity

| **Table 4.10gi Predicted means, mean changes, and effect sizes for Caregiver PSC outcome with imputed data among lower 50% Child Wellbeing Scores** | | | | | | | | | | | | |
| --- | --- | --- | --- | --- | --- | --- | --- | --- | --- | --- | --- | --- |
|  |  | **EASE (N=36)** | | |  | **PYA (N=58)** | | |  | **Between group treatment effect** | | |
|  |  | *Mean*  *(95% CI)^a^* |  | *Mean change from baseline*  *(95% CI)*  *p* |  | *Mean*  *(95% CI)^a^* |  | *Mean change from baseline*  *(95% CI)*  *p* |  | *Mean difference in change*  *(95% CI)^b^*  *p* |  | *Cohen’s d^c^* |
| T0 |  | 30.8  (27.2, 34.5) |  | - |  | 29.3  (26.4, 32.3) |  | - |  | - |  | - |
| T1 |  | 29.3  (25.6, 33.1) |  | -1.5  (-4.9, 2.0)  .40 |  | 27.9  (24.8, 30.9) |  | -1.5  (-4.2, 1.2)  .29 |  | -0.03  (-4.4, 4.4)  .99 |  | 0.01 |
| ^a^Means, SDs, difference in mean change are based on coefficients and combination of coefficients from mixed effects model following multiple imputation.  ^b^Negative mean difference in change in this table means that the *decrease* in PSC score was larger in EASE than PYA  ^c^ Cohen’s d effect size was calculated by dividing the predicted difference in mean change from the mixed effects model by the pooled baseline SD (10.9).  ^*^Model included fixed effects of arm, time and arm X time interaction, and random effects of pt_code, family ID, and Ease group.  T0=Baseline, T1=Endline | | | | | | | | | | | | |

| **Table 4.10gii Predicted means, mean changes, and effect sizes for Caregiver PSC outcome with imputed data among upper 50% Child Wellbeing Scores** | | | | | | | | | | | | |
| --- | --- | --- | --- | --- | --- | --- | --- | --- | --- | --- | --- | --- |
|  |  | **EASE (N=44)** | | |  | **PYA (N=60)** | | |  | **Between group treatment effect** | | |
|  |  | *Mean*  *(95% CI)^a^* |  | *Mean change from baseline*  *(95% CI)*  *p* |  | *Mean*  *(95% CI)^a^* |  | *Mean change from baseline*  *(95% CI)*  *p* |  | *Mean difference in change*  *(95% CI)^b^*  *p* |  | *Cohen’s d^c^* |
| T0 |  | 26.4  (23.6, 29.2) |  | - |  | 25.7  (23.3, 28.2) |  | - |  | - |  | - |
| T1 |  | 25.2  (22.4, 28.0) |  | -1.2  (-3.9, 1.6)  .40 |  | 27.3  (24.5, 30.0) |  | 1.5  (-1.1, 4.1)  .26 |  | -2.7  (-6.5, 1.1)  .17 |  | 0.30 |
| ^a^Means, SDs, difference in mean change are based on coefficients and combination of coefficients from mixed effects model following multiple imputation.  ^b^Negative mean difference in change in this table means that the *decrease* in PSC score was larger in EASE than PYA  ^c^ Cohen’s d effect size was calculated by dividing the predicted difference in mean change from the mixed effects model by the pooled baseline SD (9.0).  ^*^Model included fixed effects of arm, time and arm X time interaction, and random effects of pt_code, family ID, and Ease group.  T0=Baseline, T1=Endline | | | | | | | | | | | | |

### 4.10h Caregiver PSC: by Caregiver PSC Severity

| **Table 4.10hi Predicted means, mean changes, and effect sizes for Caregiver PSC outcome with imputed data among lower 50% Caregiver PSC Scores** | | | | | | | | | | | | |
| --- | --- | --- | --- | --- | --- | --- | --- | --- | --- | --- | --- | --- |
|  |  | **EASE (N=36)** | | |  | **PYA (N=59)** | | |  | **Between group treatment effect** | | |
|  |  | *Mean*  *(95% CI)^a^* |  | *Mean change from baseline*  *(95% CI)*  *p* |  | *Mean*  *(95% CI)^a^* |  | *Mean change from baseline*  *(95% CI)*  *p* |  | *Mean difference in change*  *(95% CI)^b^*  *p* |  | *Cohen’s d^c^* |
| T0 |  | 19.0  (16.2, 21.7) |  | - |  | 19.7  (17.2, 22.2) |  | - |  | - |  | - |
| T1 |  | 20.6  (17.8, 23.5) |  | 1.7  (-1.2, 4.6)  .26 |  | 21.6  (19.1, 24.2) |  | 1.9  (-0.39, 4.2)  .10 |  | -0.24  (-4.0, 3.5)  .90 |  |  |
| ^a^Means, SDs, difference in mean change are based on coefficients and combination of coefficients from mixed effects model following multiple imputation.  ^b^Negative mean difference in change in this table means that the *increase* in PSC score was smaller in EASE than PYA  ^c^ Cohen’s d effect size was calculated by dividing the predicted difference in mean change from the mixed effects model by the pooled baseline SD (5.4).  ^*^Model included fixed effects of arm, time and arm X time interaction, and random effects of pt_code, family ID, and Ease group.  T0=Baseline, T1=Endline | | | | | | | | | | | | |

| **Table 4.10hii Predicted means, mean changes, and effect sizes for Caregiver PSC outcome with imputed data among upper 50% Caregiver PSC Scores** | | | | | | | | | | | | |
| --- | --- | --- | --- | --- | --- | --- | --- | --- | --- | --- | --- | --- |
|  |  | **EASE (N=44)** | | |  | **PYA (N=59)** | | |  | **Between group treatment effect** | | |
|  |  | *Mean*  *(95% CI)^a^* |  | *Mean change from baseline*  *(95% CI)*  *p* |  | *Mean*  *(95% CI)^a^* |  | *Mean change from baseline*  *(95% CI)*  *p* |  | *Mean difference in change*  *(95% CI)^b^*  *p* |  | *Cohen’s d^c^* |
| T0 |  | 36.3  (33.7, 39.0) |  | - |  | 35.4  (33.1, 37.7) |  | - |  | - |  | - |
| T1 |  | 32.6  (29.9, 35.2) |  | -3.8  (-6.7, -0.85)  **.01** |  | 33.6  (31.0, 36.1) |  | -1.8  (-4.5, 0.86)  .18 |  | -1.9  (-5.9, 2.0)  .34 |  | 0.31 |
| ^a^Means, SDs, difference in mean change are based on coefficients and combination of coefficients from mixed effects model following multiple imputation.  ^b^Negative mean difference in change in this table means that the *decrease* in PSC score was larger in EASE than PYA  ^c^ Cohen’s d effect size was calculated by dividing the predicted difference in mean change from the mixed effects model by the pooled baseline SD (6.1).  ^*^Model included fixed effects of arm, time and arm X time interaction, and random effects of pt_code, family ID, and Ease group.  T0=Baseline, T1=Endline | | | | | | | | | | | | |

### 4.10i Caregiver PSC: by Caregiver K6 Severity

| **Table 4.10ii Predicted means, mean changes, and effect sizes for Caregiver PSC outcome with imputed data among lower 50% Caregiver K6 Scores** | | | | | | | | | | | | |
| --- | --- | --- | --- | --- | --- | --- | --- | --- | --- | --- | --- | --- |
|  |  | **EASE (N=45)** | | |  | **PYA (N=48)** | | |  | **Between group treatment effect** | | |
|  |  | *Mean*  *(95% CI)^a^* |  | *Mean change from baseline*  *(95% CI)*  *p* |  | *Mean*  *(95% CI)^a^* |  | *Mean change from baseline*  *(95% CI)*  *p* |  | *Mean difference in change*  *(95% CI)^b^*  *p* |  | *Cohen’s d^c^* |
| T0 |  | 25.5  (21.5, 29.5) |  | - |  | 24.1  (15.4, 32.8) |  | - |  | - |  | - |
| T1 |  | 23.6  (20.0, 27.6) |  | -1.9  (-4.8, 1.0)  .21 |  | 25.4  (16.6, 34.1) |  | 1.3  (-1.8, 4.3)  .42 |  | -3.2  (-7.4, 1.0)  .14 |  | 0.30 |
| ^a^Means, SDs, difference in mean change are based on coefficients and combination of coefficients from mixed effects model following multiple imputation.  ^b^Negative mean difference in change in this table means that the *decrease* in PSC score was larger in EASE than PYA  ^c^ Cohen’s d effect size was calculated by dividing the predicted difference in mean change from the mixed effects model by the pooled baseline SD (10.6).  ^*^Model included fixed effects of arm, time and arm X time interaction, and random effects of pt_code, family ID, and Ease group.  T0=Baseline, T1=Endline | | | | | | | | | | | | |

| **Table 4.10iii Predicted means, mean changes, and effect sizes for Caregiver PSC outcome with imputed data among upper 50% Caregiver K6 Scores** | | | | | | | | | | | | |
| --- | --- | --- | --- | --- | --- | --- | --- | --- | --- | --- | --- | --- |
|  |  | **EASE (N=35)** | | |  | **PYA (N=70)** | | |  | **Between group treatment effect** | | |
|  |  | *Mean*  *(95% CI)^a^* |  | *Mean change from baseline*  *(95% CI)*  *p* |  | *Mean*  *(95% CI)^a^* |  | *Mean change from baseline*  *(95% CI)*  *p* |  | *Mean difference in change*  *(95% CI)^b^*  *p* |  | *Cohen’s d^c^* |
| T0 |  | 32.0  (28.4, 35.5) |  | - |  | 30.3  (26.4, 34.1) |  | - |  | - |  | - |
| T1 |  | 31.4  (27.9, 34.8) |  | -0.59  (-4.1, 2.9)  .74 |  | 29.5  (25.5, 33.4) |  | -0.78  (-3.1, 1.6)  .51 |  | 0.19  (-4.1, 4.5)  .93 |  | 0.02 |
| ^a^Means, SDs, difference in mean change are based on coefficients and combination of coefficients from mixed effects model following multiple imputation.  ^b^Positive mean difference in change in this table means that the *decrease* in PSC score was larger in PYA than EASE  ^c^ Cohen’s d effect size was calculated by dividing the predicted difference in mean change from the mixed effects model by the pooled baseline SD (8.6).  ^*^Model included fixed effects of arm, time and arm X time interaction, and random effects of pt_code, family ID, and Ease group.  T0=Baseline, T1=Endline | | | | | | | | | | | | |

### 4.10j Caregiver PSC: by Treatment Completer Status

| **Table 4.10ji Predicted means, mean changes, and effect sizes for Caregiver PSC outcome with imputed data among treatment completers (child)** | | | | | | | | | | | | |
| --- | --- | --- | --- | --- | --- | --- | --- | --- | --- | --- | --- | --- |
|  |  | **EASE (N=56)** | | |  | **PYA (N=117)** | | |  | **Between group treatment effect** | | |
|  |  | *Mean*  *(95% CI)^a^* |  | *Mean change from baseline*  *(95% CI)*  *p* |  | *Mean*  *(95% CI)^a^* |  | *Mean change from baseline*  *(95% CI)*  *p* |  | *Mean difference in change*  *(95% CI)^b^*  *p* |  | *Cohen’s d^c^* |
| T0 |  | 28.8  (25.2, 32.3) |  | - |  | 27.7  (19.8, 35.5) |  | - |  | - |  | - |
| T1 |  | 28.0  (24.4, 31.6) |  | -0.77  (-3.4, 1.8)  .57 |  | 27.7  (19.8, 35.6) |  | 0.02  (-1.9, 2.0)  .99 |  | -0.78  (-4.1, 2.5)  .64 |  | 0.08 |
| ^a^Means, SDs, difference in mean change are based on coefficients and combination of coefficients from mixed effects model following multiple imputation.  ^b^Negative mean difference in change in this table means that the *decrease* in PSC score was larger in EASE than PYA  ^c^ Cohen’s d effect size was calculated by dividing the predicted difference in mean change from the mixed effects model by the pooled baseline SD (10.0).  ^*^Model included fixed effects of arm, time and arm X time interaction, and random effects of pt_code, family ID, and Ease group.  T0=Baseline, T1=Endline | | | | | | | | | | | | |

##

## Partial Response Rate: Child PHQ

| **Table 4.22a Predicted percent with 50% or greater reduction in Child PHQ symptoms at T1 and risk ratio comparing difference in percentage between EASE and PYA** | | | | | |
| --- | --- | --- | --- | --- | --- |
| **EASE (N=80)** | **PYA (N=118)** | | **Between group treatment effect** | | |
| *Percent*  *(95% CI)^a^* | | |  | *Risk Ratio*  *(95% CI)*  *p* | |
| 23.8  (13.1, 34.4) |  | 21.5  (12.8, 30.1) |  | | 1.11  (0.44, 1.78)  .74 |
| ^a^Percent, 95% Cis and Risk Ratio are based on coefficients and combination of coefficients from GLM with log link following multiple imputation.  ^b^Risk Ratio >1 suggests a larger ‘risk’ of having a 50% or greater reduction in EASE than PYA | | | | | |

| **Table 4.22b Predicted percent with 30-49% reduction in Child PHQ symptoms at T1 and risk ratio comparing difference in percentage between EASE and PYA** | | | | | |
| --- | --- | --- | --- | --- | --- |
| **EASE (N=80)** | **PYA (N=118)** | | **Between group treatment effect** | | |
| *Percent*  *(95% CI)^a^* | | |  | *Risk Ratio*  *(95% CI)*  *p* | |
| 11.3  (3.9, 18.6) |  | 13.2  (6.4, 20.1) |  | | 0.85  (0.14, 1.56)  .70 |
| ^a^Percent, 95% Cis and Risk Ratio are based on coefficients and combination of coefficients from GLM with log link following multiple imputation.  ^b^Risk Ratio <1 suggests a larger ‘risk’ of having a 30-49% reduction in PYA than EASE | | | | | |

##

## Partial Response Rate: Child PSC Internalizing

| **Table 4.23a Predicted percent with 50% or greater reduction in Child PSC Internalizing symptoms at T1 and risk ratio comparing difference in percentage between EASE and PYA** | | | | | |
| --- | --- | --- | --- | --- | --- |
| **EASE (N=80)** | **PYA (N=118)** | | **Between group treatment effect** | | |
| *Percent*  *(95% CI)^a^* | | |  | *Risk Ratio*  *(95% CI)*  *p* | |
| 35.7  (22.3, 49.0) |  | 37.5  (26.3, 48.7) |  | | 0.95  (0.50, 1.40) |
| ^a^Percent, 95% Cis and Risk Ratio are based on coefficients and combination of coefficients from GLM with log link following multiple imputation.  ^b^Risk Ratio <1 suggests a larger ‘risk’ of having a 50% reduction in PYA than EASE | | | | | |

| **Table 4.23b Predicted percent with 30-49% reduction in Child PSC Internalizing symptoms at T1 and risk ratio comparing difference in percentage between EASE and PYA** | | | | | |
| --- | --- | --- | --- | --- | --- |
| **EASE (N=80)** | **PYA (N=118)** | | **Between group treatment effect** | | |
| *Percent*  *(95% CI)^a^* | | |  | *Risk Ratio*  *(95% CI)*  *p* | |
| 13.8  (5.6, 21.9) |  | 13.3  (6.7, 20.0) |  | | 1.03  (0.23, 1.83)  .94 |
| ^a^Percent, 95% Cis and Risk Ratio are based on coefficients and combination of coefficients from GLM with log link following multiple imputation.  ^b^Risk Ratio >1 suggests a larger ‘risk’ of having a 30-49% reduction in EASE than PYA | | | | | |

## Emotional Coping by Treatment Completion Status

| **Table 4.24a Predicted means, mean changes, and effect sizes for Child EmoCop outcome with imputed data among treatment completers** | | | | | | | | | | | | |
| --- | --- | --- | --- | --- | --- | --- | --- | --- | --- | --- | --- | --- |
|  |  | **EASE (N=56)** | | |  | **PYA (N=117)** | | |  | **Between group treatment effect** | | |
|  |  | *Mean*  *(95% CI)^a^* |  | *Mean change from baseline*  *(95% CI)*  *p* |  | *Mean*  *(95% CI)^a^* |  | *Mean change from baseline*  *(95% CI)*  *p* |  | *Mean difference in change*  *(95% CI)^b^*  *p* |  | *Cohen’s d^c^* |
| T0 |  | 16.0  (14.5, 17.4) |  | - |  | 14.6  (13.6, 15.6) |  | - |  | - |  | - |
| T1 |  | 15.6  (14.2, 17.1) |  | -0.37  (-2.2, 1.5)  .70 |  | 14.7  (13.7, 15.8) |  | 0.09  (-1.2, 1.4)  .89 |  | -0.46  (-2.7, 1.8)  .69 |  | 0.08 |
| ^a^Means, SDs, difference in mean change are based on coefficients and combination of coefficients from mixed effects model following multiple imputation.  ^b^Negative mean difference in change in this table means that the *decrease* in EmoCop score was larger in EASE than PYA  ^c^ Cohen’s d effect size was calculated by dividing the predicted difference in mean change from the mixed effects model by the pooled baseline SD (5.7).  ^*^Model included fixed effects of arm, time and arm X time interaction, and random effects of pt_code, family ID, and Ease group.  T0=Baseline, T1=Endline | | | | | | | | | | | | |

| **Table 4.24b Predicted means, mean changes, and effect sizes for Caregiver EmoCop outcome with imputed data among treatment completers** | | | | | | | | | | | | |
| --- | --- | --- | --- | --- | --- | --- | --- | --- | --- | --- | --- | --- |
|  |  | **EASE (N=56)** | | |  | **PYA (N=117)** | | |  | **Between group treatment effect** | | |
|  |  | *Mean*  *(95% CI)^a^* |  | *Mean change from baseline*  *(95% CI)*  *p* |  | *Mean*  *(95% CI)^a^* |  | *Mean change from baseline*  *(95% CI)*  *p* |  | *Mean difference in change*  *(95% CI)^b^*  *p* |  | *Cohen’s d^c^* |
| T0 |  | 18.6  (17.2, 20.0) |  | - |  | 19.6  (18.6, 20.6) |  | - |  | - |  | - |
| T1 |  | 19.2  (17.8, 20.6) |  | 0.58  (-1.0, 2.2)  .48 |  | 19.6  (18.6, 20.6) |  | -0.02  (-1.1, 1.1)  .98 |  | 0.59  (-1.4, 2.6)  .55 |  | 0.11 |
| ^a^Means, SDs, difference in mean change are based on coefficients and combination of coefficients from mixed effects model following multiple imputation.  ^b^Positive mean difference in change in this table means that the *increase* in EmoCop score was larger in EASE than PYA  ^c^ Cohen’s d effect size was calculated by dividing the predicted difference in mean change from the mixed effects model by the pooled baseline SD (5.5).  ^*^Model included fixed effects of arm, time and arm X time interaction, and random effects of pt_code, family ID, and Ease group.  T0=Baseline, T1=Endline | | | | | | | | | | | | |

## Emotional Coping as a Predictor of Child Mental Health

| **Table 4.25a Emotional Coping (Child report at T0) as a predictor of Child PSC at T1 among the EASE Group** | | | | | |
| --- | --- | --- | --- | --- | --- |
| **EmoCop Top 50%**  **(N=46)** | **EmoCop Bottom 50%**  **(N=34)** | | **Between group treatment effect** | | |
| *Mean PSC Score at T1*  *(95% CI)^a^* | | |  | *Beta Coefficient*  *(95% CI)*  *p* | |
| 22.4  (19.3, 25.5) |  | 21.5  (17.8, 25.2) |  | | 0.90  (-3.9, 5.7)  .71 |
| ^a^Predicted means, 95% Cis and beta coefficient are based on coefficients and combination of coefficients from linear regression following multiple imputation.  ^b^Positive Beta coefficient suggests a greater mean PSC score among those with top 50% EmoCop score | | | | | |

| **Table 4.25b Emotional Coping (Child report at T0) as a predictor of Child PSC Internalizing at T1 among the EASE Group** | | | | | |
| --- | --- | --- | --- | --- | --- |
| **EmoCop Top 50%**  **(N=46)** | **EmoCop Bottom 50%**  **(N=34)** | | **Between group treatment effect** | | |
| *Mean PSC Score at T1*  *(95% CI)^a^* | | |  | *Beta Coefficient*  *(95% CI)*  *p* | |
| 3.9  (3.2, 4.6) |  | 3.7  (2.9, 4.5) |  | | 0.17  (-0.92, 1.3)  .75 |
| ^a^Predicted means, 95% Cis and beta coefficient are based on coefficients and combination of coefficients from linear regression following multiple imputation.  ^b^Positive Beta coefficient suggests a greater mean PSC Internalizing score among those with top 50% EmoCop score | | | | | |

| **Table 4.25c Emotional Coping (Child report at T0) as a predictor of Child PHQ Internalizing at T1 among the EASE Group** | | | | | |
| --- | --- | --- | --- | --- | --- |
| **EmoCop Top 50%**  **(N=46)** | **EmoCop Bottom 50%**  **(N=34)** | | **Between group treatment effect** | | |
| *Mean PSC Score at T1*  *(95% CI)^a^* | | |  | *Beta Coefficient*  *(95% CI)*  *p* | |
| 8.4  (6.4, 10.3) |  | 8.2  (5.9, 10.5) |  | | 0.17  (-2.8, 3.2)  .91 |
| ^a^Predicted means, 95% Cis and beta coefficient are based on coefficients and combination of coefficients from linear regression following multiple imputation.  ^b^Positive Beta coefficient suggests a greater mean PHQ score among those with top 50% EmoCop score | | | | | |
